# Microbial transformation of anti-cancer steroid exemestane and cytotoxicity of its metabolites against cancer cell lines

**DOI:** 10.1186/1752-153X-7-57

**Published:** 2013-03-27

**Authors:** Elias Baydoun, Marium Bibi, Muhammad Asif Iqbal, Atia-tul Wahab, Dina Farran, Colon Smith, Samina A Sattar, Atta-ur Rahman, M Iqbal Choudhary

**Affiliations:** 1 American University of Beirut, Beirut, 1107 2020, Lebanon; 2 H. E. J. Research Institute of Chemistry, International Center for Chemical and Biological Sciences, University of Karachi, Karachi, 75270, Pakistan; 3 Dr. Panjwani Center for Molecular Medicine and Drug Research, International Center for Chemical and Biological Sciences, University of Karachi, Karachi, 75270, Pakistan; 4 Department of Biochemistry, Faculty of Science, King Abdulaziz University, Jeddah, 21412, Saudi Arabia

**Keywords:** Steroid, Exemestane, Anti-cancer activity, Cancer cell lines (HeLa, PC3), *Fusarium lini*, *Macrophomina phaseolina*, Microbial transformation

## Abstract

**Background:**

Microbial transformation of steroids has been extensively used for the synthesis of steroidal drugs, that often yield novel analogues, not easy to obtain by chemical synthesis. We report here fungal transformation of a synthetic steroidal drug, exemestane, used for the treatment of breast cancer and function through inhibition of aromatase enzyme.

**Results:**

Microbial transformation of anti-cancer steroid, exemestane (**1**), was investigated by using two filamentous fungi. Incubation of **1** with fungi *Macrophomina phaseolina*, and *Fusarium lini* afforded three new, 11α-hydroxy-6-methylene-androsta-1, 4-diene-3,17-dione (**2**), 16β, 17β-dihydroxy-6-methylene-androsta-1, 4-diene-3-one (**3**), and 17β-hydroxy-6-methylene-androsta-1, 4-diene-3, 16-dione (**4**), and one known metabolites, 17β-hydroxy-6-methylene-androsta-1, 4-diene-3-one (**5**). Their structures were deduced spectroscopically. Compared to **1** (steroidal aromatase inactivator), the transformed metabolites were also evaluated for cytotoxic activity by using a cell viability assay against cancer cell lines (HeLa and PC3). Metabolite **2** was found to be moderately active against both the cell lines.

**Conclusions:**

Biotransformation of exemestane (**1**) provides an efficient method for the synthesis of new analogues of **1**. The metabolites were obtained as a result of reduction of double bond and hydroxylation. The transformed product **2** exhibited a moderate activity against cancer cell lines (HeLa and PC3). These transformed products can be studied for their potential as drug candidates.

## Background

Microbial transformation of steroids has been extensively employed for the synthesis of steroidal drugs, both at laboratory and industrial levels [1-7]. In modern drug discovery process, generation of libraries of bioactive compounds with diverse structures plays an important role [8].

Exemstane (trade name aromasin) is a steroidal irreversible aromatase inhibitor, used for the treatment of breast cancer. Breast cancers have estrogen receptors (ER-positive) and their growth depends on aromatase activity. Therefore, inhibition of aromatase enzyme reduces the estrogen levels and thus slows the growth of breast cancer [9-12].

Interestingly exemestane not only increases the testosterone level and lowers estrogen, but it also increases the levels of insulin-like growth factor (IGF) [10]. The large reduction in estrogen levels combined with a rise in IGF, makes exemestane an effective breast cancer medication [13-15]. Based on the importance of exemestane in the treatment of breast cancer, a number of exemestane derivatives were previously synthesized involving modification of C-6 methylene and reduction of C-17 keto group, and evaluated for their aromatase inhibitory potential [16-19].

During current study, we synthesized new analogues of 1 by biotransformation techniques. Screening experiments showed that *Macrophomina phaseolina* and *Fusarium lini* were able to efficiently transform **1** into several metabolites. Subsequent large scale fermentations produced three new metabolites **2**-**4** along with a known metabolite **5**. The structures of metabolites were unambiguously established through detailed spectral analysis. The microbial transformed metabolites **2** and **4** of exemestane showed a moderate anti-cancer effect against PC3 and/or Hela cancer cell lines. This successful attempt to synthesize new derivatives of an anti-cancer steroid may lead to the discovery of new cancer therapeutic agents.

## Results and discussion

**Figure 1:**
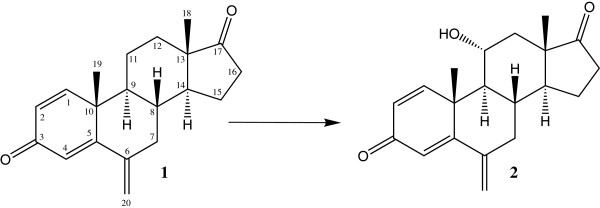
**Biotransformation of exemestane (1) with *Fusarium lini *yielded metabolite 2.**

**Figure 2:**
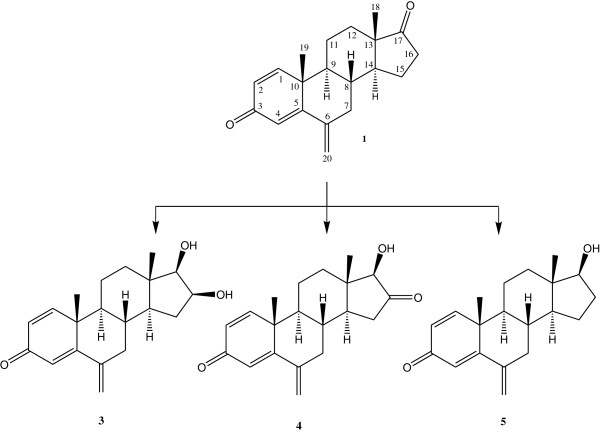
**Biotransformation of exemestane (1) with *Macrophomina phaseolina *yielded metabolites 3-5.**

Four microbial metabolites were generated by the selected fungal strains, i.e. *Macrophomina phaseolina* and *Fusarium lini* (Figures 1 and 2). *M. phaseolina* is previously reported to catalyze the introduction of double bond between C-1 and C-2, hydroxyl groups at C-6, C-15, C-16 and C-17, and carbonyl group at C-17 of the steroidal skeleton [1,20]. *F. lini* is also reported to catalyze the oxidation at C-1, C-2, C-6, and C-11 of steroidal skeleton [21]. The chemical structures of the metabolites **2**-**4** are reported here for the first time along with their NMR data (Tables 1 and 2).

**Table 1:** ^**1**^**H-NMR data of compounds 1–5 in ppm, *J *in Hz**

**COMPOUNDS**					
**Carbon**	**1**^**a**^	**2**^**a**^	**3**^**b**^	**4**^**b**^	**5**^**b**^
**1**	7.21 d (10.0)	7.21 d (10.5)	7.31 d (10.2)	7.34 d (10.5)	7.31 d (10.2)
**2**	6.14 dd (10.5, 2.0)	6.12 dd (10.5, 2.0)	6.21 dd (10.2, 1.8)	6.42, dd (10.5, 2.0)	6.22 dd (10.2, 1.8)
**3**	-	-	-	-	-
**4**	5.99, d (2.0)	6.00 d (2.0)	6.08 d (1.8)	6.11 d (2.0)	6.09 d (1.8)
**5**	-	-	-	-	-
**6**	-	-	-	-	-
**7**	2.69, 1.97 m	2.13, 1.28 m	2.56 d (9.0), 1.87 m	2.57, 1.96 m	2.66, 1.82 m
**8**	2.03 m	1.98 m	1.86 m	1.98 m	1.83 m
**9**	1.34 m	1.62 m	1.35 m	1.48 m	1.05 m
**10**	-	-	-	-	-
**11**	1.92, 1.76 m	4.30 m	1.91, 1.31 m,	1.93, 1.84 m	1.75, 1.83 m
**12**	1.78, 1.28 m	2.13, 1.28 m	1.90, 1.16 m	2.01, 1.45 m	1.13, 1.92 m
**13**	-	-	-	-	-
**14**	1.43 m	1.37 m	0.93 m	1.63 m	1.27 m
**15**	1.99, 1.67 m	1.93, 1.80 m	2.20, 1.30 m	2.29, 1.95 m	1.65, 1.38 m
**16**	2.41, 1.95 m	2.66, 1.95 m	4.07 m	-	2.00, 1.51 m
**17**	-	-	3.30 d (7.5)	3.77 s	3.55 t (8.7)
**18**	0.92 s	0.99 s	0.99 s	0.81 s	0.81 s
**19**	1.19 s	1.18 s	1.18 s	1.21 s	1.17 s
**20**	5.03, 5.01 s	5.04, 5.02 s	5.02 t (1.9)	5.06, 5.04 s	4.99, 5.01 s

^a^ 500 MHz (CD_3_)_2_CO.

^b^ 300 MHz CD_3_OD.

**Table 2:** ^**13**^**C-NMR data of compounds 1–5 in ppm**

**COMPOUNDS**					
**Carbon**	**1**^**a**^	**2**^**b**^	**3**^**c**^	**4**^**d**^	**5**^**d**^
**1**	155.0	154.9	158.1	157.6	158.2
**2**	128.0	128.0	127.8	127.9	127.7
**3**	185.8	185.8	188.7	188.6	188.7
**4**	122.9	122.9	122.7	122.8	122.7
**5**	167.9	167.9	171.6	171.2	171.7
**6**	147.1	147.0	147.7	147.3	147.8
**7**	39.87	32.1	41.4	41.2	41.3
**8**	36.0	39.6	36.6	35.9	37.2
**9**	50.9	48.6	51.9	51.4	51.7
**10**	44.3	44.3	45.6	45.5	45.6
**11**	22.63	71.5	23.0	23.2	23.6
**12**	32.0	32.0	38.1	36.6	37.5
**13**	48.1	48.1	43.7	43.5	44.2
**14**	51.3	50.8	48.2	45.4	51.8
**15**	22.3	22.3	35.9	36.6	24.3
**16**	35.8	39.7	70.6	217.7	30.5
**17**	218.8	218.0	81.7	86.8	82.1
**18**	13.9	14.5	12.5	11.9	11.6
**19**	20.1	20.1	20.1	20.1	20.1
**20**	112.2	112.2	112.6	112.9	112.4

^a^ 125 MHz (CD_3_)_2_CO.

^b^ 150 MHz (CD_3_)_2_CO.

^c^ 125 MHz CD_3_OD.

^d^ 75 MHz CD_3_OD.

The anti-cancer effect of exemestane (**1**) [2] and its synthetic analogues on HeLa and PC3 were determined by using MTT assay. Results obtained from these assays are presented in Table 3.

**Table 3:** ***In vitro *cytotoxicity of compounds 1–5**

**Compound Codes**	**HeLa (Cervical cancer) (IC**_**50**_**± S.D.) μM**	**PC-3 (Prostate cancer) (IC**_**50 **_**±S.D.) μM**
**1**	>50	>50
**2**	16.83±0.96	24.87±0.72
**3**	>50	>50
**4**	37.20 ± 0.88	>50
**5**	>50	>50
**Doxorubicin**	**3.10 ± 0.20**	**0.91 ± 0.12**

**Figure 3:**
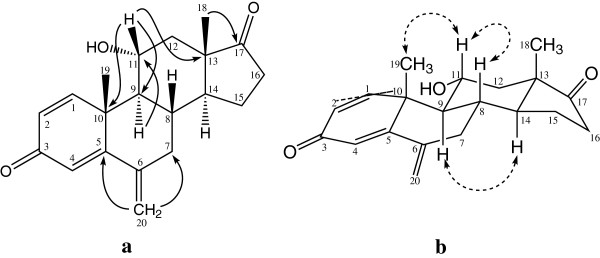
Important HMBC (a) and NOESY (b) correlations in metabolite 2.

The molecular formula C_20_H_24_O_3_ [*M*^*+*^, *m/z* 312] of metabolite **2** was deduced from the HREI-MS (*M*^*+*^*m/z* 312.1705), suggested the addition of an oxygen in substrate **1**. The ^1^H-NMR spectral analysis of **2** (Table 1) displayed a downfield methine signal, as compared to the starting material exemestane (**1**), resonating at δ 4.30 (m, *W*_1/2_ = 15.6 Hz), while its respective carbon signal was at δ 71.5 in ^13^C-NMR spectrum (Table 2). The HMBC spectrum (Figure 3) displayed long-range couplings of the hydroxyl-bearing methine proton (δ 4.30) with C-9 (δ 48.6), C-10 (δ 44.3), and C-13 (δ 48.1), which suggested the position of the hydroxyl-bearing methine at C-11. H-11 also showed COSY cross peaks with H-9 (δ 1.62) and H_2_-12 (δ 1.28, 2.13). The stereochemical assignments were based on NOESY interactions (Figure 3) between H-11 (δ 4.30), H-8 (δ 1.98), and Me-19 (δ 1.18). H-11 was thus deduced as β-oriented. Metabolite **2** was finally identified as 11α-hydroxy-6-methylene-androsta-1,4-diene-3,17-dione.

**Figure 4:**
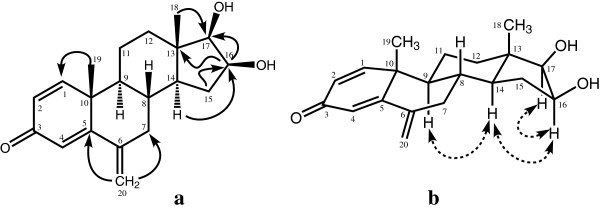
Important HMBC (a) and NOESY (b) correlations in metabolite 3.

Molecular composition of metabolite **3** was deduced to be C_20_H_26_O_3_ from the HREI-MS analysis (*M*^*+*^ = *m/z* 314.1933, calcd 314.1882). The ^1^H-NMR spectra μm (Table 1) of metabolite **3** showed two hydroxyl-bearing methine proton peaks at δ 3.30 (d, *J*_17,16_ = 7.5 Hz, H-17) and 4.07 (m, *W*_*1/2*_ = 20.0 Hz). The ^13^C-NMR spectrum of **3** lacks signal for C-17 carbonyl, whereas new methine carbon at δ 81.7 suggested the reduction of C-17 ketone into C-17 OH. The proton geminal to the –OH group (δ 4.07) was correlated with C-13 (δ 43.7), C-14 (δ 48.2) and C-17 (δ 81.7) in the HMBC spectrum. The methine C-17 (δ 81.7) showed HMBC correlations with H-14 (δ 0.93, m) and H-18 (δ 0.99, s). Based on the above observations, the hydroxyl-bearing methine carbon was identified as C-16. The H-16 (δ 4.07) showed NOESY cross peaks with H-14 (δ 0.93), but no interaction with H-18 (δ 0.99) (Figure 4). Therefore the C-16 proton was assigned to be α-oriented. The metabolite **3** was thus identified as 16β, 17β-dihydroxy-6-methylene-androsta-1, 4-diene-3-one.

**Figure 5:**
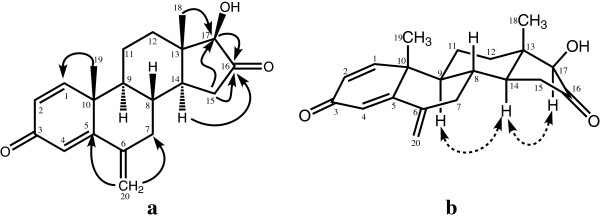
Important HMBC (a) and NOESY (b) correlations in metabolite 4.

Molecular formula C_20_H_24_O_3_ (*M*^*+*^*m/z* 312.1725, calcd 312.1720) was deduced from the HREI-MS of metabolite **4**. A distinct downfield methine proton signal appeared at δ 3.77 (br. s, *W*_1/2_ = 9.3 Hz) in the ^1^H-NMR spectrum of **4**. The ^13^C-NMR spectrum showed a saturated ketone carbon signal at δ 217.7. The rest of the spectrum was distinctly similar to metabolite **2**. The deshielded methine proton was HMBC correlated with this ketonic carbon, while its corresponding methine carbon at δ 86.8 showed the HMBC correlations with H_2_-15 (δ 1.95, 2.29), and C*H*_*3*_-18 (δ 0.81). These interactions, along with appearance of a downfield proton (δ 3.77), indicated that the ketone at C-17 has been reduced into an –OH. Geminal H-17 (δ 3.77) showed NOESY correlations with H-14 (δ 1.63), indicating it to be *axially* (α-) oriented. The saturated ketone carbon (δ 217.7) was place at C-16, based on the above mentioned HMBC correlations (Figure 5). The structure of metabolite **4** was finally identified as 17β-hydroxy-6-methylene-androsta-1, 4-diene-3, 16-dione.

Metabolite **5** has a molecular composition C_20_H_26_O_2_ (HREI-MS, *M*^*+*^*m/z* 298.1730, calcd 298.1733). Based on ^1^H- and ^13^C-NMR spectral data (Tables 1 and 2), compound **5** was identified as 17β-hydroxy-6-methylene-androsta-1, 4-diene-3-one. It has previously been reported as an *in-vitro* cytochrome P_450_-mediated transformed product of exemestane [22].

The cytotoxic effect of the compounds **1-5** against two tumor cell lines, PC-3 (prostate cancer cell) and Hela (cervical cancer cell), was evaluated (Table 3) using the MTT assay. Compound **2** showed a moderate cytotoxicity against both the cancer cell line with IC_50_ = 16.83 ± 0.96 and 24.87 ± 0.72 μM, respectively, as compared to the standard drug, doxorubicin. Compound **4** exhibited a moderate activity against HeLa cell line.

## Conclusion

In conclusion, the biotransformation of exemestane (**1**) with *F. lini* and *M. phaseolina* were investigated for the first time which provided an efficient route towards the synthesis of several new metabolites **2**–**5**. Metabolite **2** was found to be moderately active against both cancer cell lines (HeLa and PC3). The work presented here can be helpful for the study of *in vivo* metabolism of exemestane (**1**), as well as for the discovery of new anticancer drugs

### Experimental

#### Substrate and chemicals

Exemestane (**1**) was purchased from local market as drug (Pfizer Canada Inc., Brand name Aromasin), extracted and further purified by flash chromatography. Thin layer chromatography (TLC) was carried out on silica gel precoated plates (PF_254_; Merck). Column chromatography (CC) was performed by using silica gel (E. Merck, Germany). Optical rotations were measured in methanol with a JASCO P-2000 polarimeter. ^1^H- and ^13^C-NMR spectra were recorded in (CD_3_)_2_CO and CD_3_OD on Bruker Avance spectrometers. The chemical shifts (δ values) are presented in ppm and the coupling constants (*J*) are in Hz. For 1D- and 2D-NMR experiments, standard Bruker pulse sequences were used. UV Spectra (in nm) were recorded in methanol with a Hitachi U-3200 spectrophotometer. Infrared (IR) spectra (in cm^-1^) were recorded with an FT-IR-8900 spectrophotometer. JEOL (Japan) JMS-600H mass spectrometer was used for recording of EI-MS and high-resolution mass spectra (HREI-MS) in *m/z* (rel. %).

The anticancer activity of compounds **2**-**5** was evaluated in 96-well flat-bottomed micro-titer plates [Iwaki, Japan] by using the standard dye MTT (3-[4, 5-dimethylthiazole-2-yl]-2, 5-diphenyl-tetrazolium bromide) [Sigma-Aldrich Chemicals, St. Louis, USA] through colorimetric analysis. For this purpose, the cells were cultured in Minimum Essential Medium (MEM), supplemented with 10% Fetal Calf Serum (FCS), 1 mmol/l sodium pyruvate, 1% (v/v) antibiotic / antimycotic and passaged weekly, using 0.25% trypsin / EDTA [Sigma-Aldrich Chemicals, St. Louis, USA] in tissue culture flasks T-75 [Iwaki, Japan]. Absorbance was taken at 540 nm wavelength by using microplate reader (Spectra Max plus, Molecular Devices, USA) using software SoftMax Pro 340 [Molecular Devices, CA, USA]. Dimethyl sulfoxide (DMSO) and doxorubicin (standard inhibitor) were purchased from Sigma-Aldrich Chemicals, [St. Louis, USA].

#### Microorganisms and culture medium

The fungi were purchased from the Northern Regional Research Laboratories (NRRL), or obtained as gift from the Karachi University Culture Collection (KUCC).

*Fusarium lini* (NRRL 2204), and *Macrophomina phaseolina* (KUCC 730) were grown in a culture medium prepared by mixing glucose (40.0 g), glycerol (40.0 mL), peptone (20.0 g), yeast extract (20.0 g), KH_2_PO_4_ (20.0 g), and NaCl (20.0 g) in distilled H_2_O (4.0 L).

#### Cell lines

PC3 (prostate cancer) and HeLa (cervical cancer) cell lines were purchased from the American Type Culture Collection (ATCC) for anticancer activity.

#### General Fermentation and Extraction Conditions

4 Liters fungal media was prepared and distributed into 40 conical flasks (100 mL in each flask). All flasks were then autoclaved at 121°C. The fungal cultures were then inoculated into each flask containing media and incubated at room temperature on shaker for three days. Compound **1** was dissolved in 40 mL methanol and distributed equally to all 40 flasks. All experimental flasks were then kept for fermentation. Two control experiments, i.e. media + compound **1** and media + fungus were also conducted. The transformation was then checked on TLC. After the detection of transformation on TLC, fungal culture from all 40 flasks was filtered and extracted with CH_2_Cl_2_ (12 L) by using liquid-liquid chromatography. The dichloromethane layer was evaporated *in vaccue*. The obtained gum was analyzed by thin-layer chromatography.

#### Fermentation and Purification of Exemestane (1) with *Fusarium lini*

Exemestane (**1**; 1.0 g) was dissolved in 40 mL methanol, and incubated with culture of *F. Lini.* The obtained gum (2.3 g) was fractionated (ARC 1-3) by using silica gel column chromatography. The mobile phase was composed of petroleum ether and acetone with a gradient of 10%. Fraction ARC-2 yielded metabolite **2** (4 mg, pet. ether: acetone = 8:2) after elution through silica gel column.

#### *11α-Hydroxy-6-methylene-androsta-1,4-diene-3,17-dione* (**2**)

Amorphous material; [α]^25^_D_: +81.4 (*c* = 0.096, MeOH); IR (KBr): ν_max_ 3408, 1657 cm^-1^; UV (MeOH): λ_max_ nm (log ε) 247 (3.78); ^1^H- and ^13^C-NMR: see Tables 1 and 2 (Additional file 1).

#### Fermentation and Purification of Exemestane (**1**) with *Macrophomina phaseolina*

Incubation of **1** (1.0 g / 40 mL methanol) with 3 days old culture of *M. phaseolina* in 40 flasks for 12 days produced the metabolites **3** (10 mg), **4** (5 mg) and **5** (6 mg).

#### *16β, 17β-Dihydroxy-6-methylene-androsta-1,4-diene-3-one* (**3**)

Amorphous material; [α]^25^_D_: +181.6 (*c* = 0.032, MeOH); IR (KBr): ν_max_ 3388, 1658 cm^-1^; UV (MeOH): λ_max_ nm (log ε) 249 (4.03); ^1^H- and ^13^C-NMR: see Tables 1, and 2 (Additional file 2).

#### *17β-Hydroxy-6-methylene-androsta-1,4-diene-3,16-dione* (**4**)

Amorphous material; [α]^25^_D_: -56.0 (*c* = 0.043, MeOH); IR (KBr): ν_max_ 3411, 1749, 1658 cm^-1^; UV (MeOH): λ_max_ nm (log ε) 247 (4.04); ^1^H- and ^13^C-NMR: see Tables 1 and 2 (Additional file 3).

#### *17β-Hydroxy-6-methylene-androsta-1,4-diene-3-one* (**5**)

Amorphous material; [α]^25^_D_: +174.5 (*c* = 0.046, MeOH); IR (KBr): ν_max_ 3421, 1657, cm^-1^; UV (MeOH): λ_max_ nm (log ε) 248 (4.24); ^1^H- and ^13^C-NMR: see Tables 1 and 2 (Additional file 4).

#### Cell Viability Assay

The cytotoxicity of metabolites **1**-**5** were determined by using MTT-based colorimetric assay in 96-well plate [23]. Both cell lines (PC-3 and HeLa) were cultured in DMEM and MEM media, respectively, in 25 cm^3^ tissue culture flasks. The media were supplemented with FBS (5%), pencillin (100 IU/mL) and streptomycin (100 mg/mL). The flasks were then incubated at 37°C in an incubator containing 5% CO_2_. The flask (80% confluence) was processed for MTT-based cytotoxicity assay. The percent viability of the cells was monitored by trypan blue dye. The cells with clear cytoplasm were considered viable. For the assay, the cells (1 × 10^5^) were loaded onto 96-well tissue culture treated plate. The plate was incubated for 24 hours at 37°C. After incubation, the cells were treated with different concentrations (1.56-50 μM dissolved in DMSO) of compounds **1**-**5** and kept in an incubator for 48 hours at 37°C. At the end of the incubation, the MTT dye (50 μL, 2 mg/mL) was added to each well and the plate was incubated for 4 hours at 37°C in an incubator. Following incubation, the insoluble formazan crystals were dissolved by adding DMSO (100 μL).

The following formula was used to analyze the cytotoxic effects of the compounds.

$$\%\;\mathrm{Inhibition}=100-\left[\left(\mathrm{Absorbance}\;\mathrm{of}\;\mathrm{test}\;\mathrm{compound}-\mathrm{Absorbance}\;\mathrm{of}\;\mathrm{blank}\right)/\left(\mathrm{Absorbance}\;\mathrm{of}\;\mathrm{control}-\mathrm{Absorbance}\;\mathrm{of}\;\mathrm{blank}\right)\times 100\right]$$

## Competing interests

The authors declare that they have no competing interests.

## Authors' contributions

EB, MIC, AR, DF, and CM participated in experimental strategy design, supervision and manuscript writing. MB and MAI carried out the experiments. AW performed NMR experiments, while SAS carried out the biological screenings. All authors read and approved the final manuscript.

## Supplementary Material

Additional file 1 **Spectroscopic data of metabolite 2.** Include spectra of EI-MS, UV, IR, ^1^H-NMR, ^13^C-NMR (BB, DEPT-135°, DEPT-90°), HMQC, HMBC, COSY-45°, NOESY experiments. Click here for file

Additional file 2 **Spectroscopic data of metabolite 3.** Include spectra of EI-MS, UV, IR, ^1^H-NMR, ^13^C-NMR (BB, DEPT-135°, DEPT-90°), HSQC, HMBC, COSY-45°, NOESY experiments. Click here for file

Additional file 3 **Spectroscopic data of metabolite 4.** Include spectra of EI-MS, UV, IR, ^1^H-NMR, ^13^C-NMR (BB, DEPT-135°, DEPT-90°), HMQC, HMBC, COSY-45°, NOESY, experiments. Click here for file

Additional file 4 **Spectroscopic data of metabolite 5.** Include spectra of EI-MS, UV, IR, ^1^H-NMR, ^13^C-NMR (BB, DEPT-135°, DEPT-90°), HSQC, HMBC, COSY-45°, NOESY experiments. Click here for file

## References

[B1] Choudhary MI Zafar S Khan NT Ahmad S Noreen S Marasini BP Al-Khedhairy AA Atta-ur-Rahman: Biotransformation of dehydroepiandrosterone with *Macrophomina phaseolina* and β-glucuronidase inhibitory activity of transformed products J Enzyme Inhib Med Chem 2012 27 348 355 10.3109/14756366.2011.590804 21774747

[B2] Choudhary MI Erum S Atif M Malik R Khan NT Atta-ur-Rahman Biotransformation of (20*S*)-20-hydroxymethylpregna-1,4-dien-3-one by four filamentous fungi Steroids 2011 76 1288 1296 21762714 10.1016/j.steroids.2011.06.007

[B3] Choudhary MI Shah SAA Atta-ur-Rahman Khan SN Khan MTH α-Glucosidase and tyrosinase inhibitors from fungal hydroxylation of tibolone and hydroxytibolones Steroids 2010 75 956 966 10.1016/j.steroids.2010.05.017 20685216

[B4] Al-Aboudi A Mohammad MY Haddad S Al-Far R Choudhary MI Atta-ur-Rahman Biotransformation of methyl cholate by *Aspergillus niger* Steroids 2009 74 483 486 10.1016/j.steroids.2009.01.002 19428436

[B5] Choudhary MI Mohammad MY Musharraf SG Parvez M Al-Aboudi A Atta-ur-Rahman New oxandrolone derivatives by biotransformation using *Rhizopus stolonifer* Steroids 2009 74 1040 1044 10.1016/j.steroids.2009.08.003 19698730

[B6] Choudhary MI Khan NT Musharraf SG Anjum S Atta-ur-Rahman Biotransformation of adrenosterone by filamentous fungus, *Cunninghamella elegans* Steroids 2007 72 923 929 10.1016/j.steroids.2007.08.002 17889091

[B7] Devkota KP Choudhary MI Nawaz SA Lannang AM Lenta BN Fokou PA Sewald N Microbial transformation of the steroidal alkaloid dictyophlebine by ***Rhizopus stolonifer*** Chem Pharm Bull 2007 55 682 684 10.1248/cpb.55.682 17409573

[B8] Tong WY Dong X Microbial biotransformation: Recent development on steroid drugs Recent Pat Biotechnol 2009 3 2 141 153 10.2174/187220809788700157 19519569

[B9] Henry NL Azzouz F Desta Z Li L Nguyen AT Lemler S Hayden J Tarpinian K Yakim E Flockhart DA Stearns V Hayes DF Storniolo AM Predictors of aromatase inhibitor discontinuation as a result of treatment-Emergent symptoms in early-stage breast cancer J Clin Oncol 2012 30 9 936 942 10.1200/JCO.2011.38.0261 22331951 PMC3341106

[B10] Mrozek E Layman R Ramaswamy B Schaaf L Li X Ottman S Shapiro CL Phase II trial of exemestane in combination with fulvestrant in postmenopausal women with advanced, Hormone-Responsive Breast Cancer Clin Breast Cancer 2012 12 2 151 156 10.1016/j.clbc.2012.01.003 22444722 PMC5003403

[B11] Debled M Le Loarer F Callonnec F Soubeyran I Cambon-Michot C Dujardin F Italiano A Complete response to exemestane in a patient with a desmoid tumor Futur Oncol 2012 8 4 483 486 10.2217/fon.12.24 22515450

[B12] Hille U Soergel P Laenger F Schippert C Makowski L Hillemanns P Aromatase inhibitors as solely treatment in postmenopausal breast cancer patients The Breast Journal 2012 18 2 145 150 10.1111/j.1524-4741.2011.01203.x 22176032

[B13] Long B Groothuis PG Hicklin D IGF1R Inhibitor based treatment of prostrate cancer International *application number: PCT/US2010/055608 Publication number: WO/2011/05706, Filing date: Nov 05, 2010*

[B14] Van-Gool SA Wit JM De-Schutter T De-Clerck N Postnov AA Hovinga SK Van-Doorn J Veiga SJ Garcia-Segura LM Karperien M Marginal growth increase, altered bone quality and polycystic ovaries in female prepubertal rats after treatment with the aromatase inhibitor exemestane Horm Res Paediatr 2010 73 1 49 60 10.1159/000271916 20190540

[B15] Yamashita H Takahashi S Ito Y Yamashita T Ando Y Toyama T Sugiura H Yoshimoto N Kobayashi S Fujii Y Hirotaka I Predictors of response to exemestane as primary endocrine therapy in estrogen receptor-positive breast cancer Cancer Sci 2009 100 11 2028 2033 10.1111/j.1349-7006.2009.01274.x 19659610 PMC11158316

[B16] Pariza RJ Yarger JG (*S*)-6-Methyloxaalkyl exemestane compounds and related methods of use 2010 US patent application number: 11/541,987 Publication number: US 2007/0088013 A1 Filing date: Oct 2, 2006 Issued patent: US7846918 (Issue date Dec 7, 2010)

[B17] Ariazi EA Leitão A Oprea TI Chen B Louis T Bertucci AM Sharma CGN Gill SD Kim HR Shupp HA Pyle JR Madrack A Donato AL Cheng D Paige JR Jordan VC Exemestane's 17-hydroxylated metabolite exerts biological effects as an androgen Mol Cancer Ther 2007 6 11 2817 2827 10.1158/1535-7163.MCT-07-0312 17989318

[B18] Buzzetti F Di Salle E Longo A Briatico G Synthesis and aromatase inhibition by potential metabolites of exemestane (6-methylenandrosta-1,4-diene-3,17-dione) Steroids 1993 58 11 527 532 10.1016/0039-128X(93)90029-M 8273115

[B19] Gorlitzer K Bonnekessel C Jones PG Palusczak A Hartmann RW Exemestan-derivate – Synthese und prüfung auf aromatase-hemmung Die Pharmazie 2006 61 575 581 16889062

[B20] Zafar S Bibi M Yousuf S Choudhary MI New metabolites from fungal biotransformation of an oral contraceptive agent: Methyloestrenolone Steroids 2013 78 4 418 425 10.1016/j.steroids.2013.01.002 23357433

[B21] Al-Maruf MA Khan NT Sakil MAA Choudhary MI Ali MU Islam MA Biotransformations of 11-ketoprogesterone by filamentous fungus, *Fusarium lini* J Sci Res 2011 3 2 347 356

[B22] Kamdem LK Flockhart DA Desta Z *In Vitro* cytochrome P450-mediated metabolism of exemestane Drug Metab Disposition 2011 39 1 98 105 10.1124/dmd.110.032276 PMC3014267 20876785

[B23] Mosmann T Rapid colorimetric assay for cellular growth and survival: Application to proliferation and cytotoxicity assays J Immunol Methods 1983 65 55 63 10.1016/0022-1759(83)90303-4 6606682

