# Supplementary material for: Microbial transformation of anti-cancer steroid exemestane and cytotoxicity of its metabolites against cancer cell lines
Source: Chem Cent J. 2013 Mar 27;7:57. doi: 10.1186/1752-153X-7-57 (PMC3637571; doi:10.1186/1752-153X-7-57)
Supplement: Additional file 1 — Spectroscopic data of metabolite 2. Include spectra of EI-MS, UV, IR, 1H-NMR, 13C-NMR (BB, DEPT-135°, DEPT-90°), HMQC, HMBC, COSY-45°, NOESY experiments. [file 1752-153X-7-57-S1.pdf]

Inlet: My Inlet Ionization mode: EI+

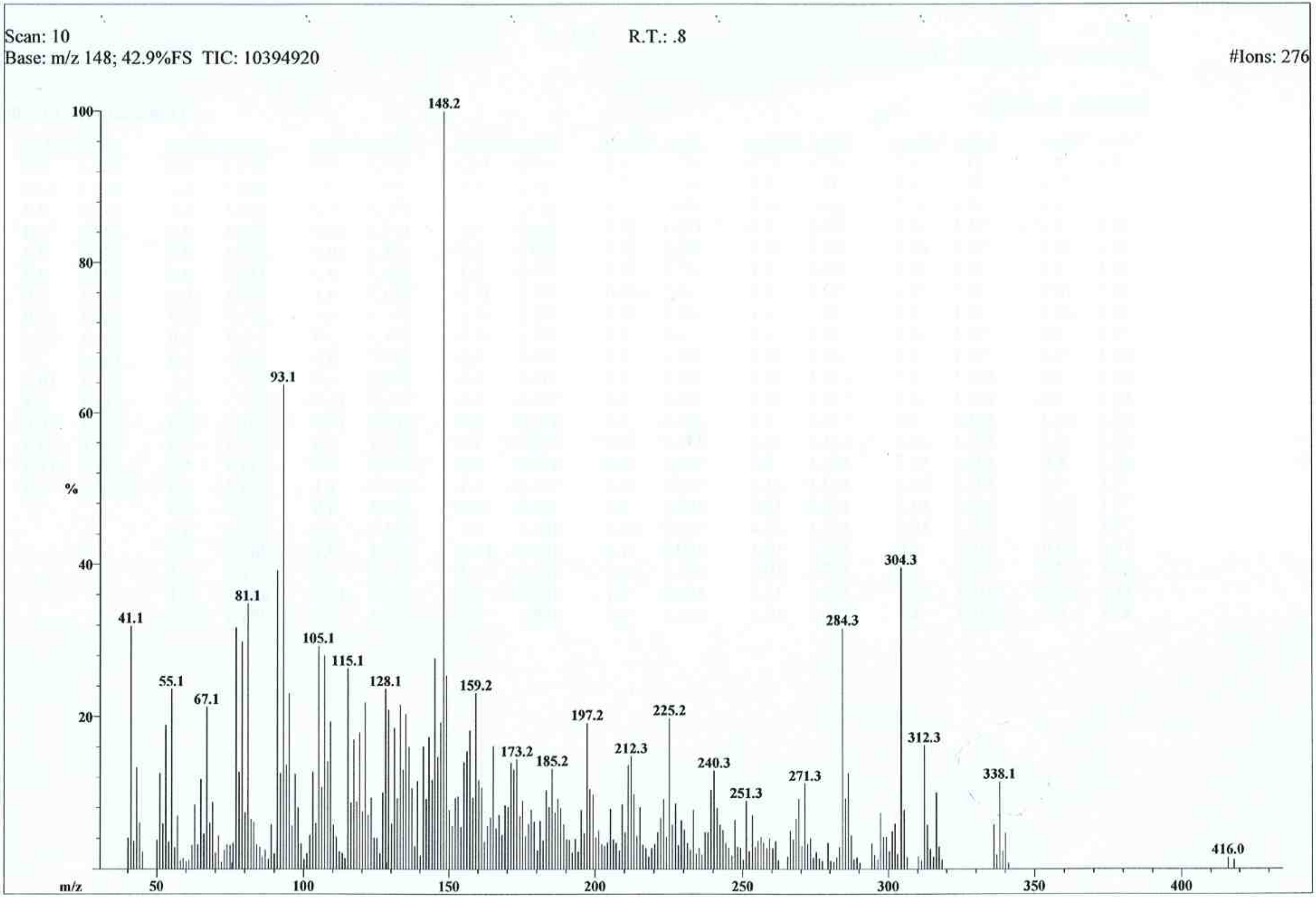

Scan Graph

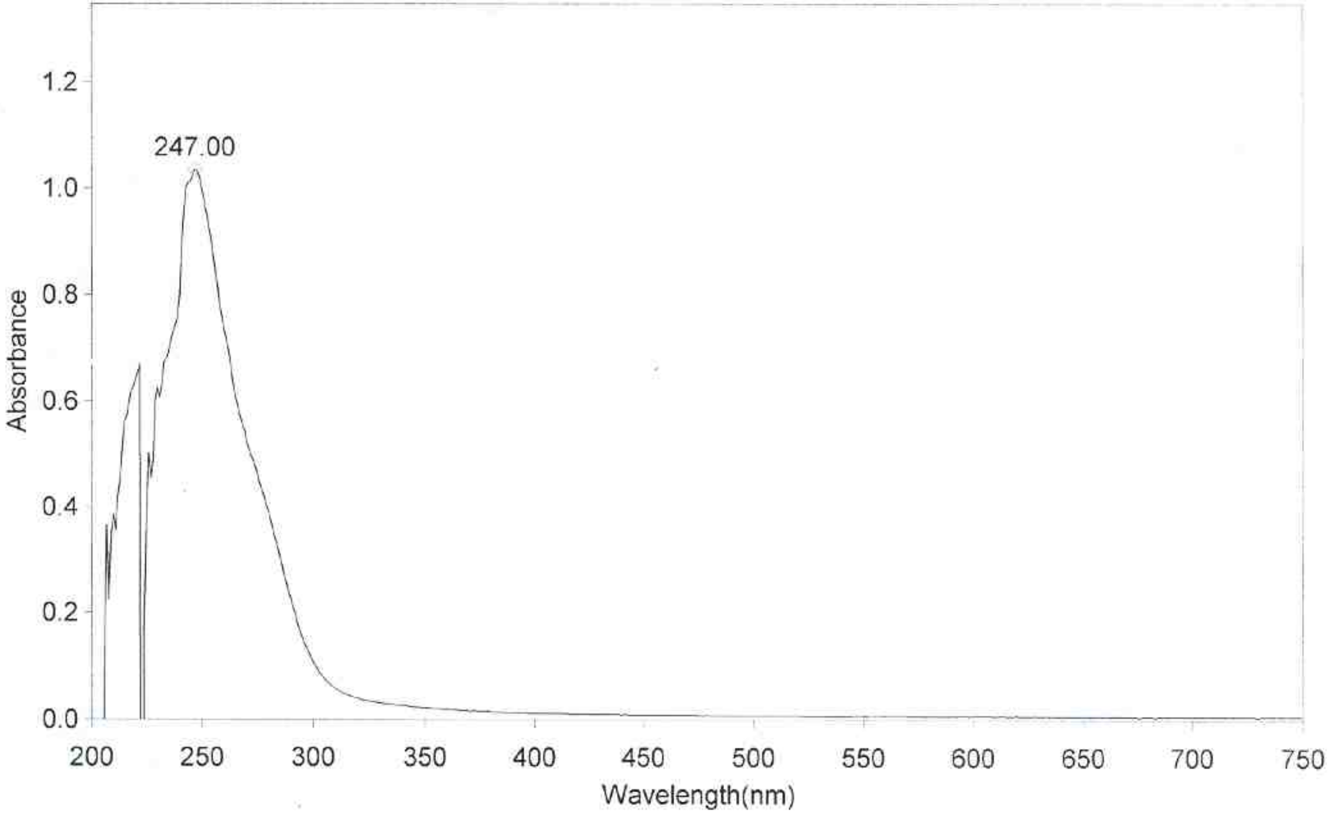
247.00
Absorbance
1.2
1.0
0.8
0.6
0.4
0.2
0.0
200
250
300
350
400
450
500
550
600
650
700
750
Wavelength(nm)

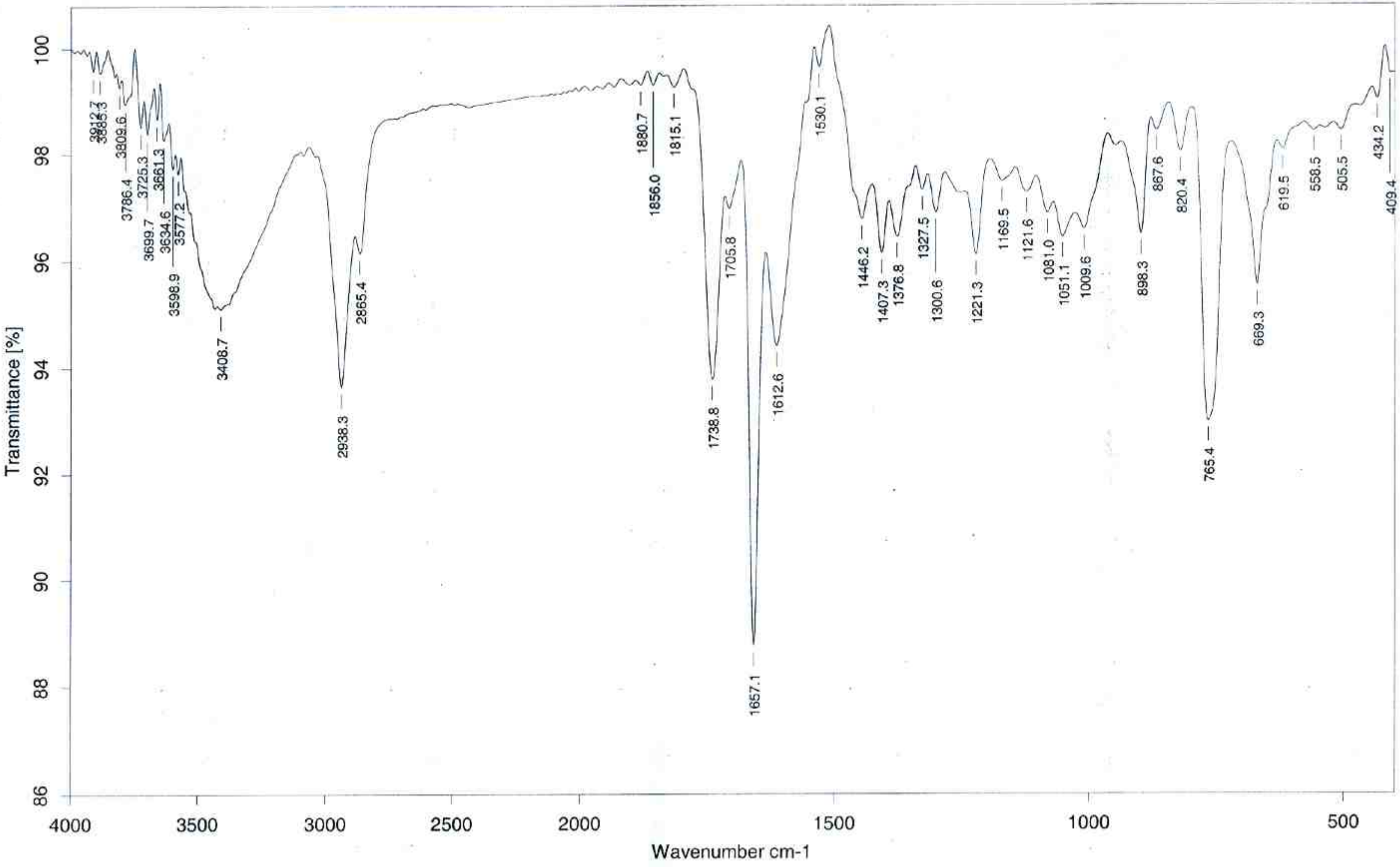
Transmittance [%]
Wavenumber cm-1
100
98
96
94
92
90
88
86
4000
3500
3000
2500
2000
1500
1000
500
3912.7
3885.3
3809.6
3786.4
3725.3
3699.7
3661.3
3634.6
3598.9
3577.2
3408.7
2938.3
2865.4
1880.7
1856.0
1815.1
1738.8
1705.8
1657.1
1612.6
1530.1
1446.2
1407.3
1376.8
1327.5
1300.6
1221.3
1169.5
1121.6
1081.0
1051.1
1009.6
898.3
867.6
820.4
765.4
669.3
619.5
558.5
505.5
434.2
409.4

LAB [illegible] 108

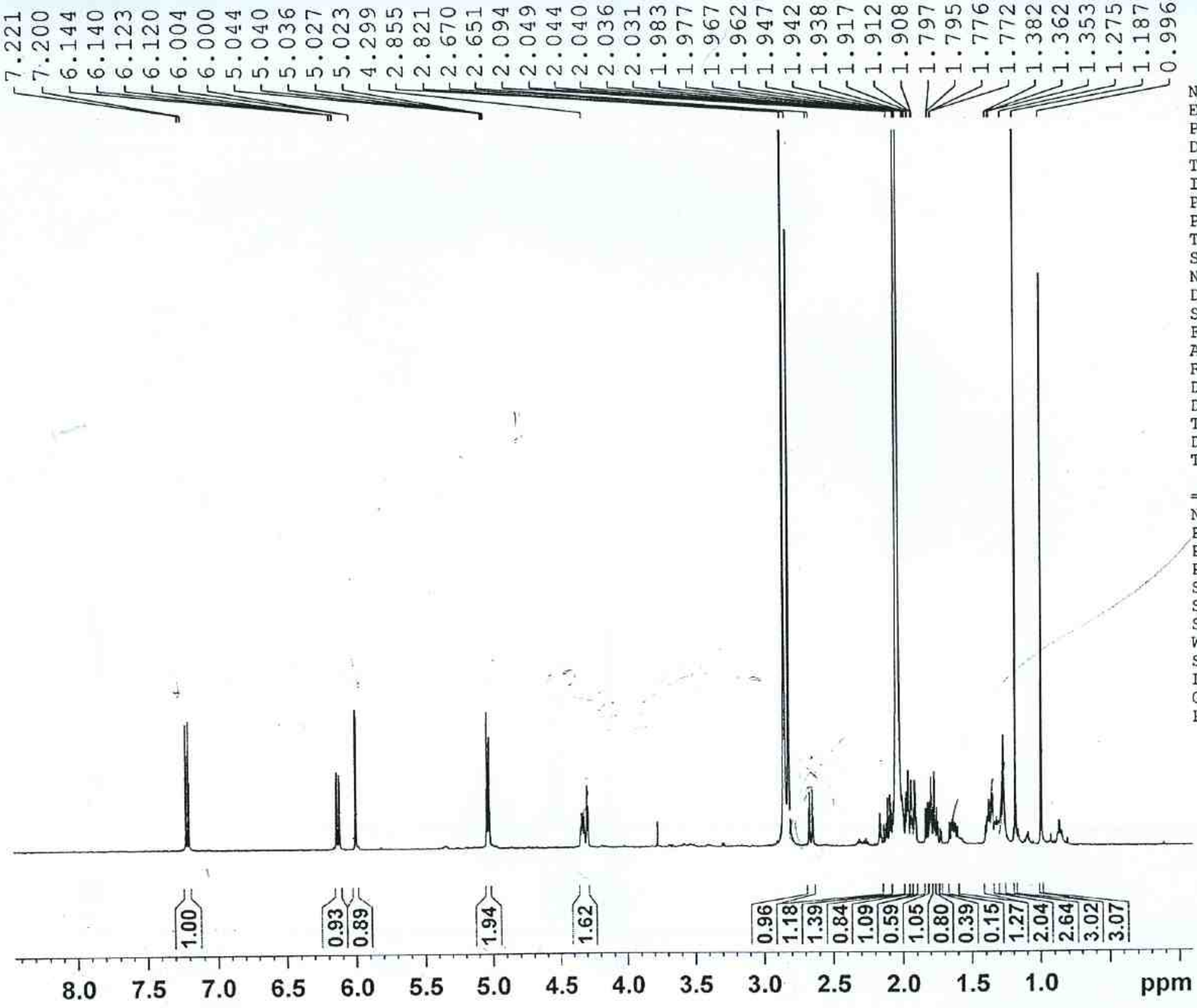

NAME	mar09-12	
EXPNO	1	
PROCNO	1	
Date_	20120309	
Time_	10.47	
INSTRUM	spect	
PROBHD	5 mm CPDUL 13C	
PULPROG	zg30	
TD	32768	
SOLVENT	Acetone	
NS	32	
DS	0	
SWH	10000.000	Hz
FIDRES	0.305176	Hz
AQ	1.6385000	sec
RG	8	
DW	50.000	usec
DE	6.50	usec
TE	293.1	K
D1	1.50000000	sec
TD0	1	

======== CHANNEL f1 ========

NUC1	1H	
P1	13.50	usec
PL1	2.00	dB
PL1W	11.25274181	W
SFO1	500.3335023	MHz
SI	32768	
SF	500.3300136	MHz
WDW	EM	
SSB	0	
LB	0.30	Hz
GB	0	
PC	1.40	

BB

LAB NO: 108

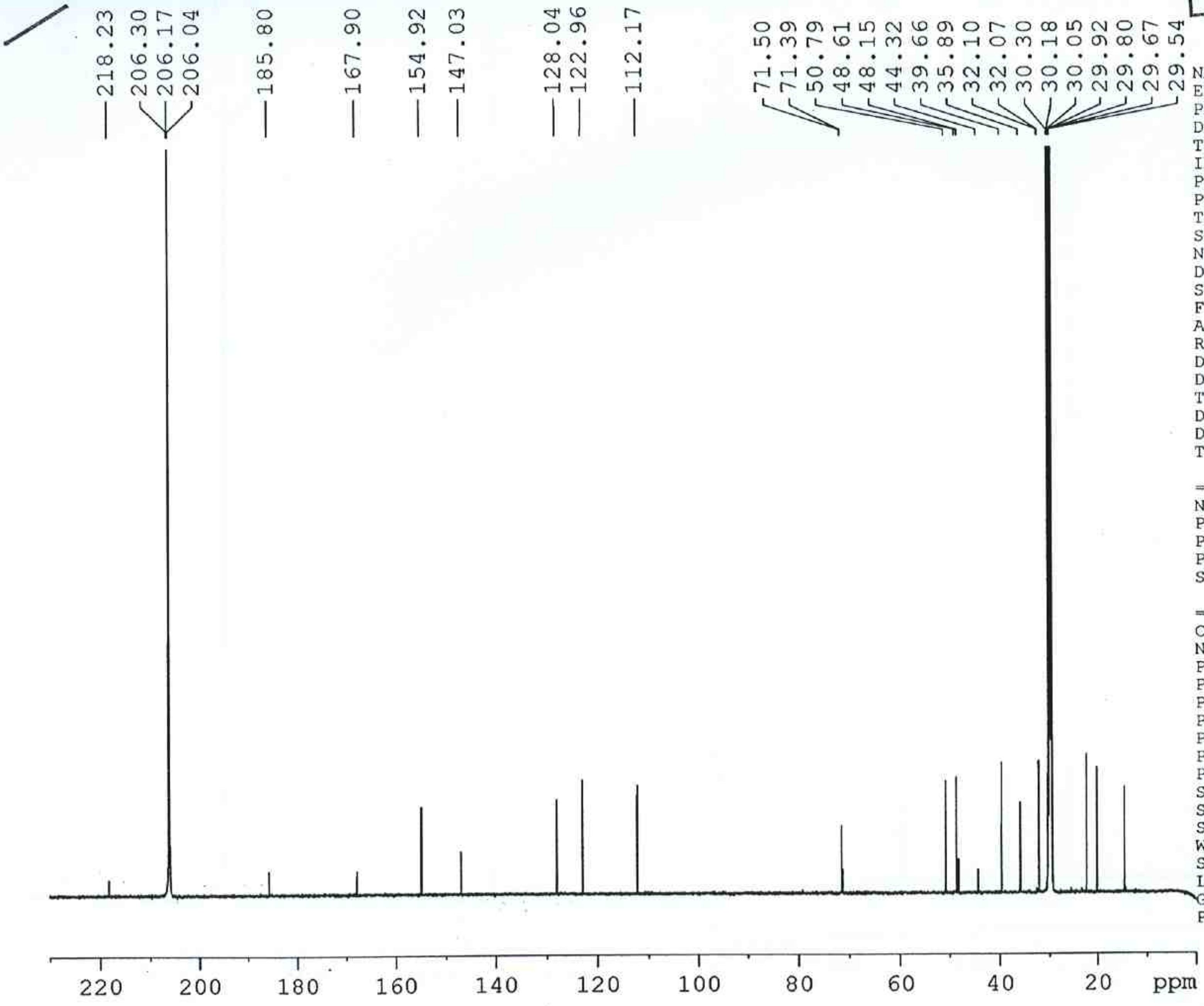

NAME	mar10-12	
EXPNO	9	
PROCNO	1	
Date_	20120311	
Time	2.50	
INSTRUM	spect	
PROBHD	5 mm CPTCI 1H-	
PULPROG	zgpg	
TD	65536	
SOLVENT	Acetone	
NS	12288	
DS	2	
SWH	35971.223	Hz
FIDRES	0.548877	Hz
AQ	0.9110143	sec
RG	32768	
DW	13.900	usec
DE	6.50	usec
TE	294.2	K
D1	1.50000000	sec
D11	0.03000000	sec
TD0	12	

======== CHANNEL f1 ========

NUC1	13C	
P1	16.00	usec
PL1	2.00	dB
PL1W	66.40702820	W
SFO1	150.9453107	MHz

======== CHANNEL f2 ========

CPDPRG2	waltz16	
NUC2	1H	
PCPD2	65.00	usec
PL2	3.30	dB
PL12	22.06	dB
PL13	27.00	dB
PL2W	9.16420078	W
PL12W	0.12192553	W
PL13W	0.03909260	W
SFO2	600.2336014	MHz
SI	32768	
SF	150.9278294	MHz
WDW	EM	
SSB	0	
LB	1.00	Hz
GB	0	
PC	1.40	

DEPT90

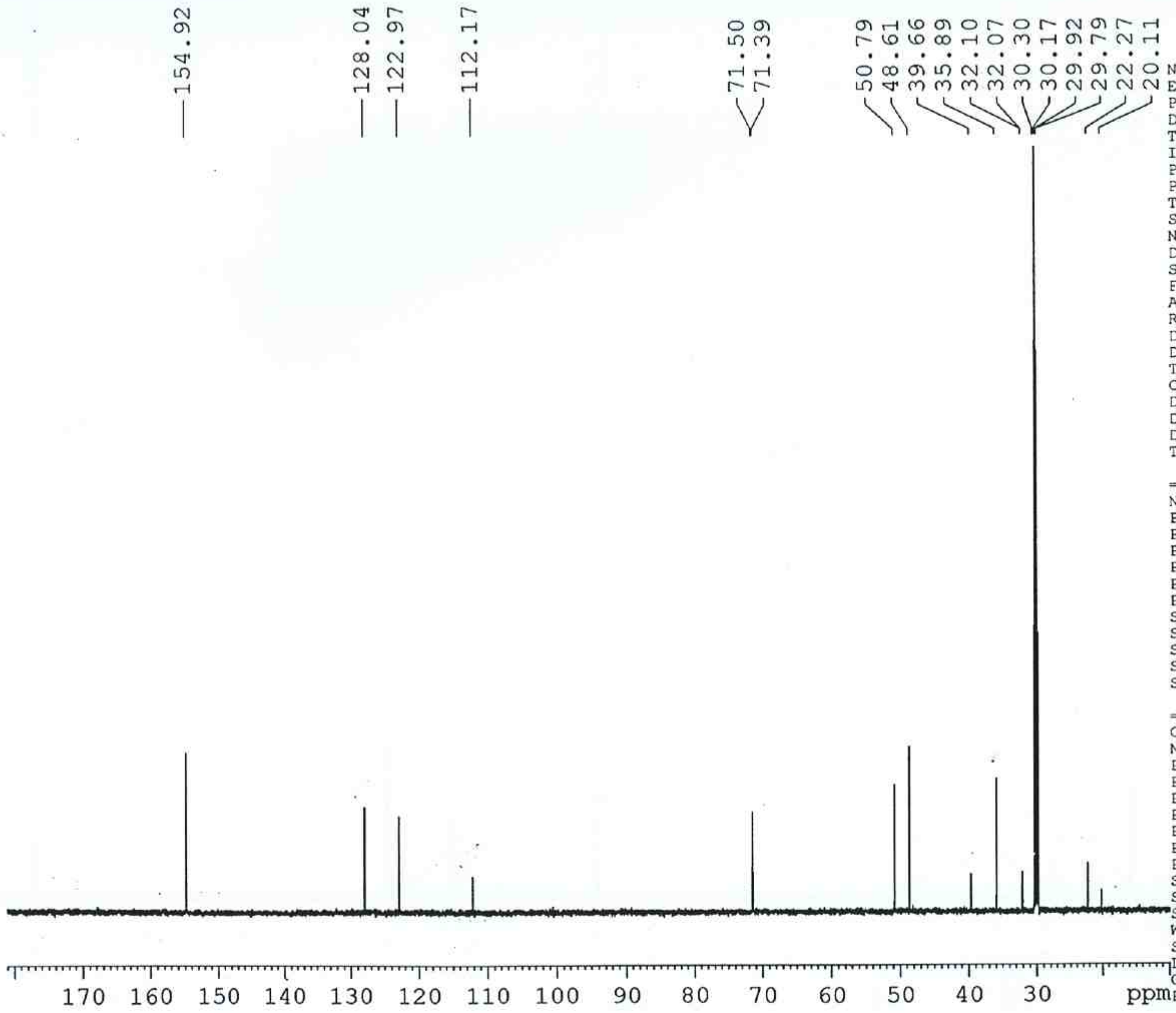

NAME	mar10-12
EXPNO	11
PROCNO	1
Date_	20120311
Time	15.49
INSTRUM	spect
PROBHD	5 mm CPTCI 1H-
PULPROG	deptsp90
TD	65536
SOLVENT	Acetone
NS	4096
DS	2
SWH	30303.031 Hz
FIDRES	0.462388 Hz
AQ	1.0814105 sec
RG	32768
DW	16.500 usec
DE	6.50 usec
TE	294.6 K
CNST2	145.0000000
D1	1.50000000 sec
D2	0.00344828 sec
D12	0.00002000 sec
TD0	4

======== CHANNEL f1 ========

NUC1	13C
P1	16.00 usec
P12	2000.00 usec
PL0	120.00 dB
PL1	2.00 dB
PL0W	0.00000000 W
PL1W	66.40702820 W
SFO1	150.9430463 MHz
SP2	1.99 dB
SPNAM2	Crp60comp.4
SPOAL2	0.500
SPOFFS2	0.00 Hz

======== CHANNEL f2 ========

CPDPRG2	waltz16
NUC2	1H
P3	7.50 usec
P4	15.00 usec
PCPD2	65.00 usec
PL2	3.30 dB
PL12	22.06 dB
PL2W	9.16420078 W
PL12W	0.12192553 W
SFO2	600.2324009 MHz
SI	32768
SF	150.9278294 MHz
WDW	EM
SSB	0
LB	1.00 Hz
GB	0
PC	1.40

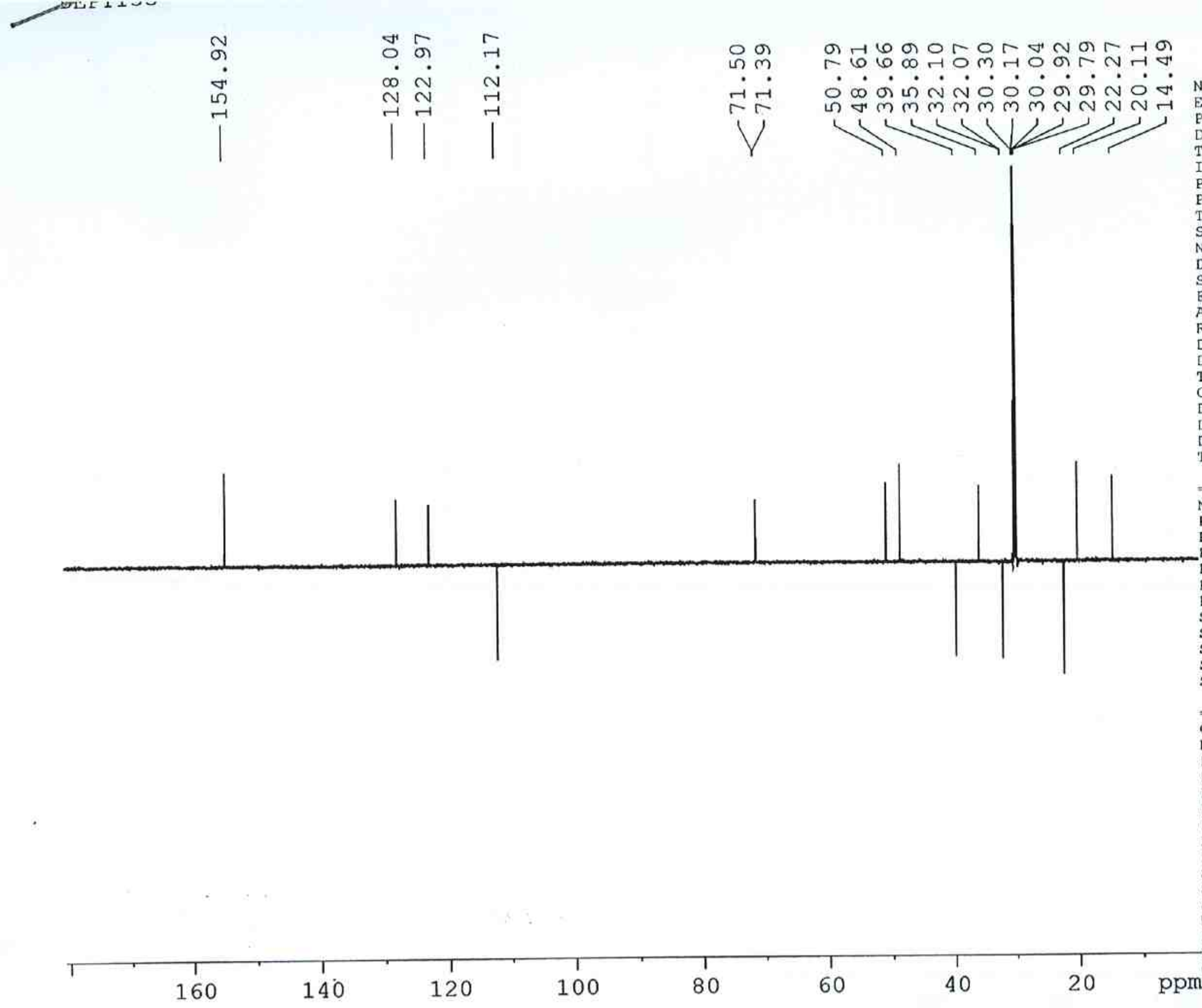

NAME	mar10-12
EXPNO	10
PROCNO	1
Date_	20120311
Time	11.19
INSTRUM	spect
PROBHD	5 mm CPTCI 1H-
PULPROG	deptsp135
TD	65536
SOLVENT	Acetone
NS	6144
DS	2
SWH	30303.031 Hz
FIDRES	0.462388 Hz
AQ	1.0814105 sec
RG	32768
DW	16.500 usec
DE	6.50 usec
TE	294.4 K
CNST2	145.0000000
D1	1.50000000 sec
D2	0.00344828 sec
D12	0.00002000 sec
TD0	6

======== CHANNEL f1 ========

NUC1	13C
P1	16.00 usec
P12	2000.00 usec
PL0	120.00 dB
PL1	2.00 dB
PL0W	0.00000000 W
PL1W	66.40702820 W
SFO1	150.9430463 MHz
SP2	1.99 dB
SPNAM2	Crp60comp.4
SPOAL2	0.500
SPOFFS2	0.00 Hz

======== CHANNEL f2 ========

CPDPRG2	waltz16
NUC2	1H
P3	7.50 usec
P4	15.00 usec
PCPD2	65.00 usec
PL2	3.30 dB
PL12	22.06 dB
PL2W	9.16420078 W
PL12W	0.12192553 W
SFO2	600.2324009 MHz
SI	32768
SF	150.9278294 MHz
WDW	EM
SSB	0
LB	1.00 Hz
GB	0
PC	1.40

HSQC

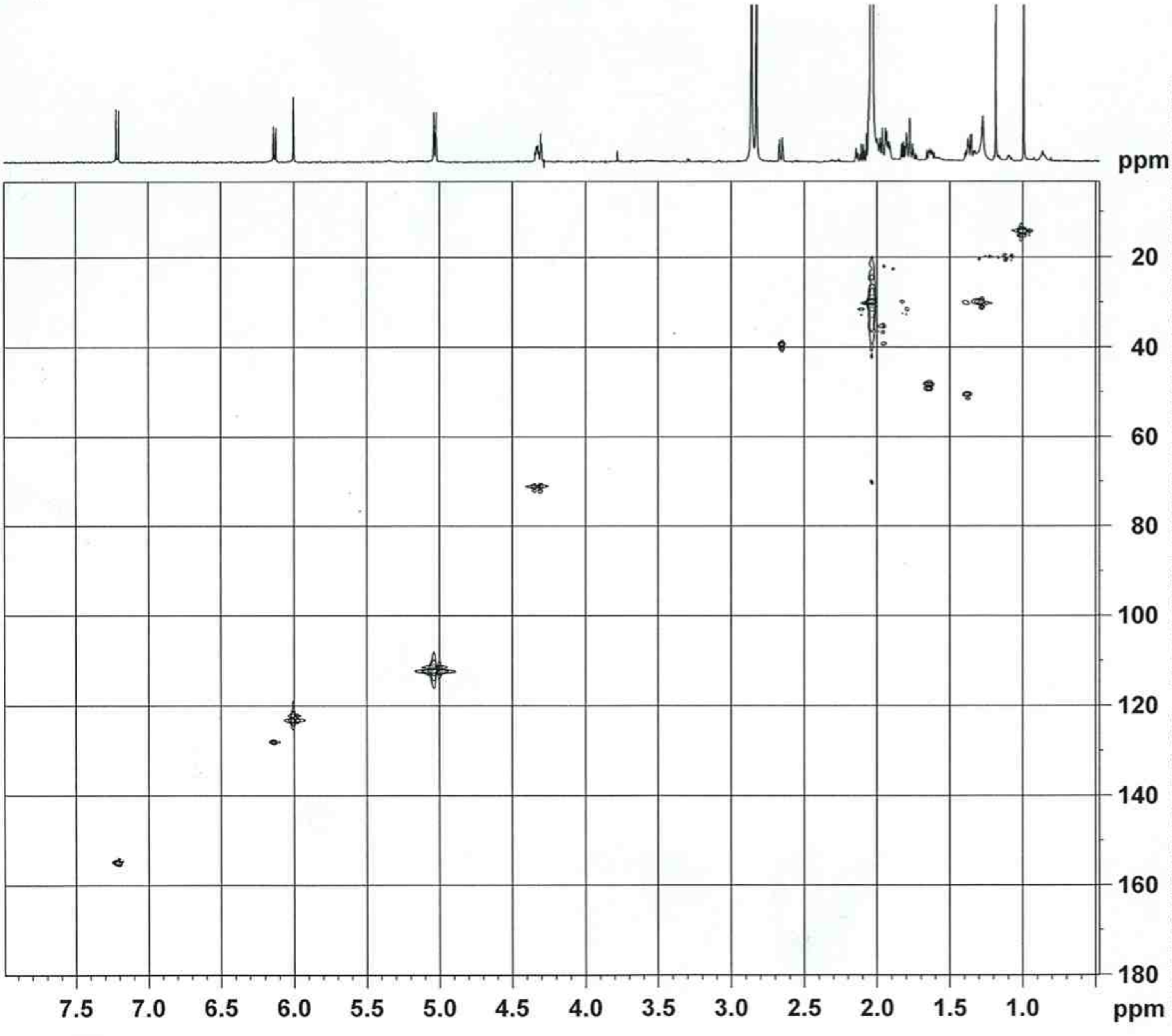

NAME	mar10-12
EXPNO	7
PROCNO	1
Date_	20120310
Time	18.02
INSTRUM	spect
PROBHD	5 mm CPTCI 1H-
PULPROG	hsqcetgpsi
TD	1024
SOLVENT	Acetone
NS	32
DS	8
SWH	5387.931 Hz
FIDRES	5.261652 Hz
AQ	0.0951700 sec
RG	46341
DW	92.800 usec
DE	6.50 usec
TE	294.2 K
CNST2	145.0000000
D0	0.00000300 sec
D1	1.50000000 sec
D4	0.00172414 sec
D11	0.03000000 sec
D13	0.00000400 sec
D16	0.00015000 sec
D24	0.00110000 sec
IN0	0.00001655 sec
ZGOPTNS	
======== CHANNEL f1 ========	
NUC1	1H
P1	7.40 usec
P2	14.80 usec
P28	0.50 usec
PL1	3.30 dB
PL1W	9.16420078 W
SFO1	600.2326410 MHz
======== CHANNEL f2 ========	
CPDPRG2	garp
NUC2	13C
P3	10.00 usec
P4	20.00 usec
PCPD2	60.00 usec
PL2	2.00 dB
PL12	17.56 dB
PL2W	66.40702820 W
PL12W	1.84592509 W
SFO2	150.9430468 MHz
====== GRADIENT CHANNEL =====	
GPNAM1	SINE.100
GPNAM2	SINE.100
GPZ1	80.00 %
GPZ2	20.10 %
P16	2000.00 usec
ND0	2
TD	256
SFO1	150.943 MHz
FIDRES	117.924255 Hz
SW	200.000 ppm
FnMODE	Echo-Antiecho
SI	1024
SF	600.2300139 MHz
WDW	QSINE
SSB	2
LB	0.00 Hz
GB	0
PC	4.00
SI	1024
MC2	echo-antiecho
SF	150.9278294 MHz
WDW	QSINE
SSB	2
LB	0.00 Hz
GB	0

[illegible]
3mm CRYOPRO[illegible]
LAB NO: 108

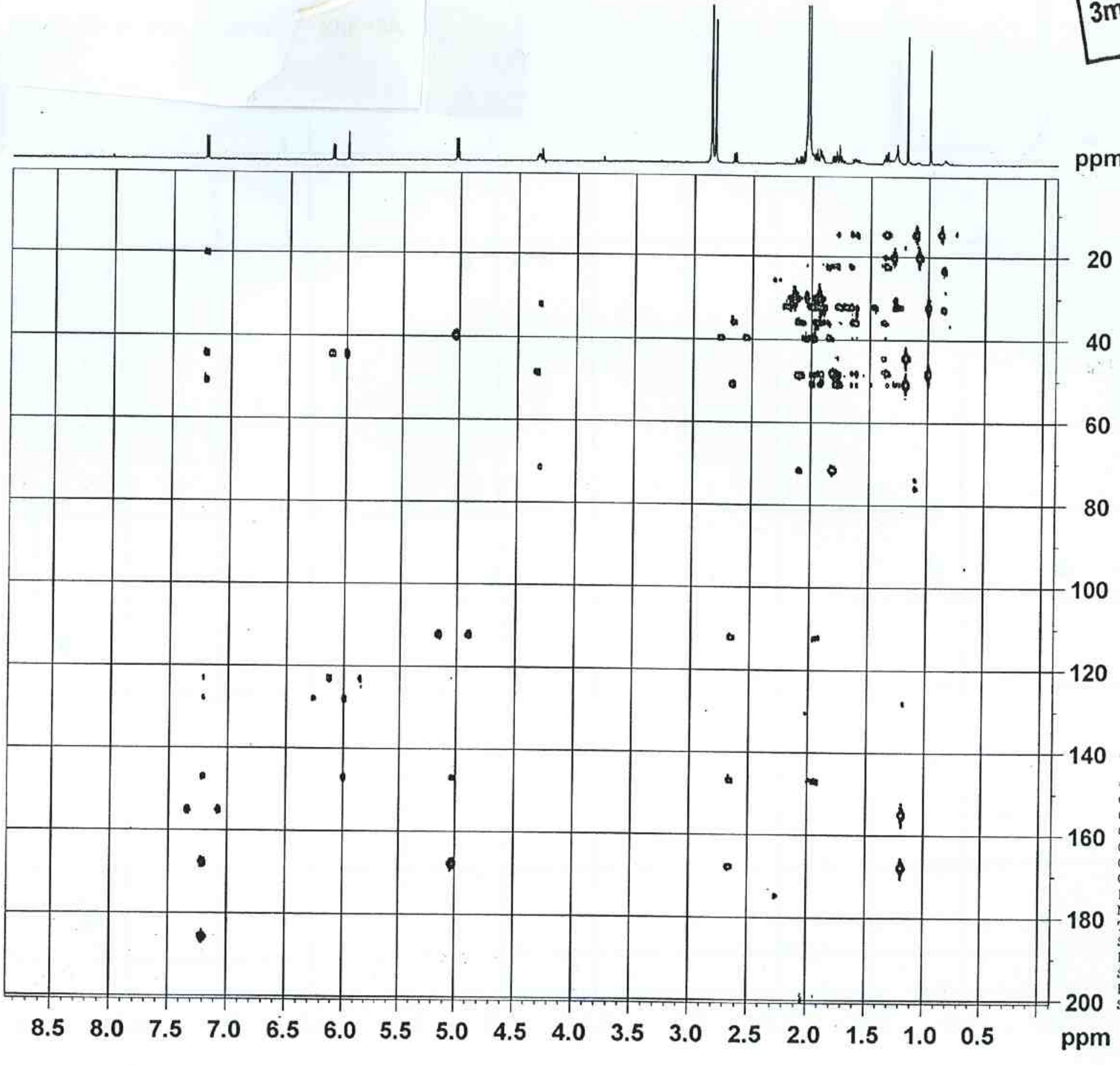

NAME	mar10-12
EXPNO	8
PROCNO	1
Date_	20120310
Time	21.44
INSTRUM	spect
PROBHD	5 mm CPTCI 1H-
PULPROG	hmbcgplpndqf
TD	4096
SOLVENT	Acetone
NS	32
DS	8
SWH	5387.931 Hz
FIDRES	1.315413 Hz
AQ	0.3802516 sec
RG	36780.8
DW	92.800 usec
DE	6.50 usec
TE	294.3 K
CNST2	145.0000000
CNST13	13.0000000
D0	0.00000300 sec
D1	1.50000000 sec
D2	0.00344828 sec
D6	0.03846154 sec
D16	0.00015000 sec
IN0	0.00001440 sec

======== CHANNEL f1 ========

NUC1	1H
P1	7.40 usec
P2	14.80 usec
PL1	3.30 dB
PL1W	9.16420078 W
SFO1	600.2326410 MHz

======== CHANNEL f2 ========

NUC2	13C
P3	10.00 usec
PL2	2.00 dB
PL2W	66.40702820 W
SFO2	150.9453107 MHz

====== GRADIENT CHANNEL =====

GPNAM1	SINE.100
GPNAM2	SINE.100
GPNAM3	SINE.100
GPZ1	50.00 %
GPZ2	30.00 %
GPZ3	40.10 %
P16	2000.00 usec
ND0	2
TD	256
SFO1	150.9453 MHz
FIDRES	135.614929 Hz
SW	230.000 ppm
FnMODE	QF
SI	1024

AVANCE AV-600-LC
3mm CRYOPROBE
LAB NO: 108

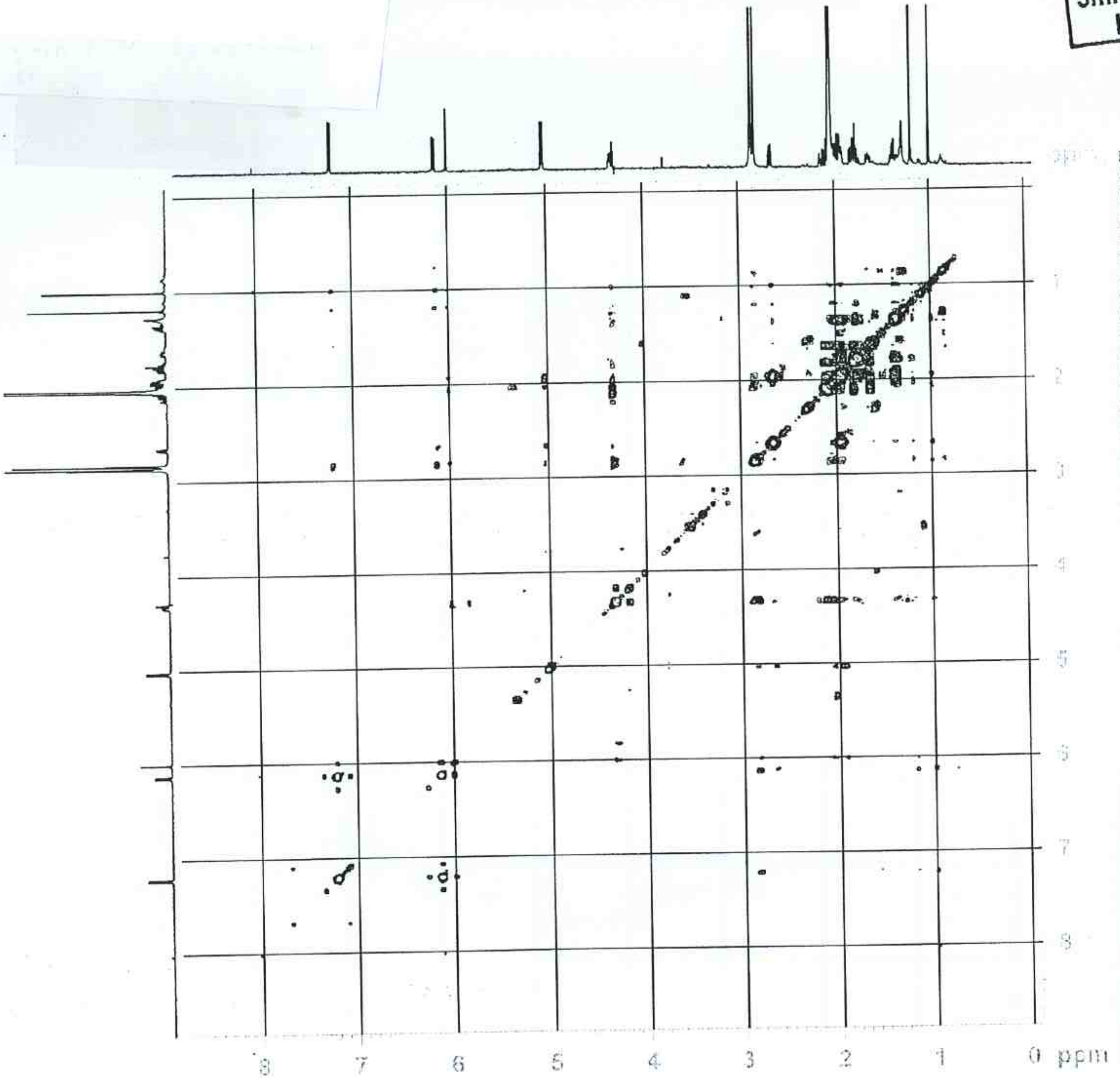

NAME	mar10-12
EXPNO	2
PROCNO	1
Date_	20120310
Time	12.31
INSTRUM	spect
PROBHD	5 mm CPTCI 1H-
PULPROG	cosydfqf
TD	2048
SOLVENT	Acetone
NS	8
DS	4
SWH	5387.931 Hz
FIDRES	2.630826 Hz
AQ	0.1901972 sec
RG	128
DW	92.800 usec
DE	6.50 usec
TE	294.4 K
D0	0.00000300 sec
D1	1.50000000 sec
D13	0.00000400 sec
D20	0.00000200 sec
IN0	0.00018560 sec

======== CHANNEL f1 ========

NUC1	1H
P1	7.40 usec
PL1	3.30 dB
PL1W	9.16420078 W
SFO1	600.2326410 MHz
ND0	1
TD	256
SFO1	600.2326 MHz
FIDRES	21.046606 Hz
SW	8.976 ppm
FnMODE	QF
SI	1024
SF	600.2300139 MHz
WDW	QSINE
SSB	0
LB	0.00 Hz
GB	0
PC	1.40
SI	1024
MC2	QF
SF	600.2300139 MHz
WDW	QSINE
SSB	0
LB	0.00 Hz
GB	0

AVANCE AV-600-LC
3mm CRYOPROBE
LAB NO: 108

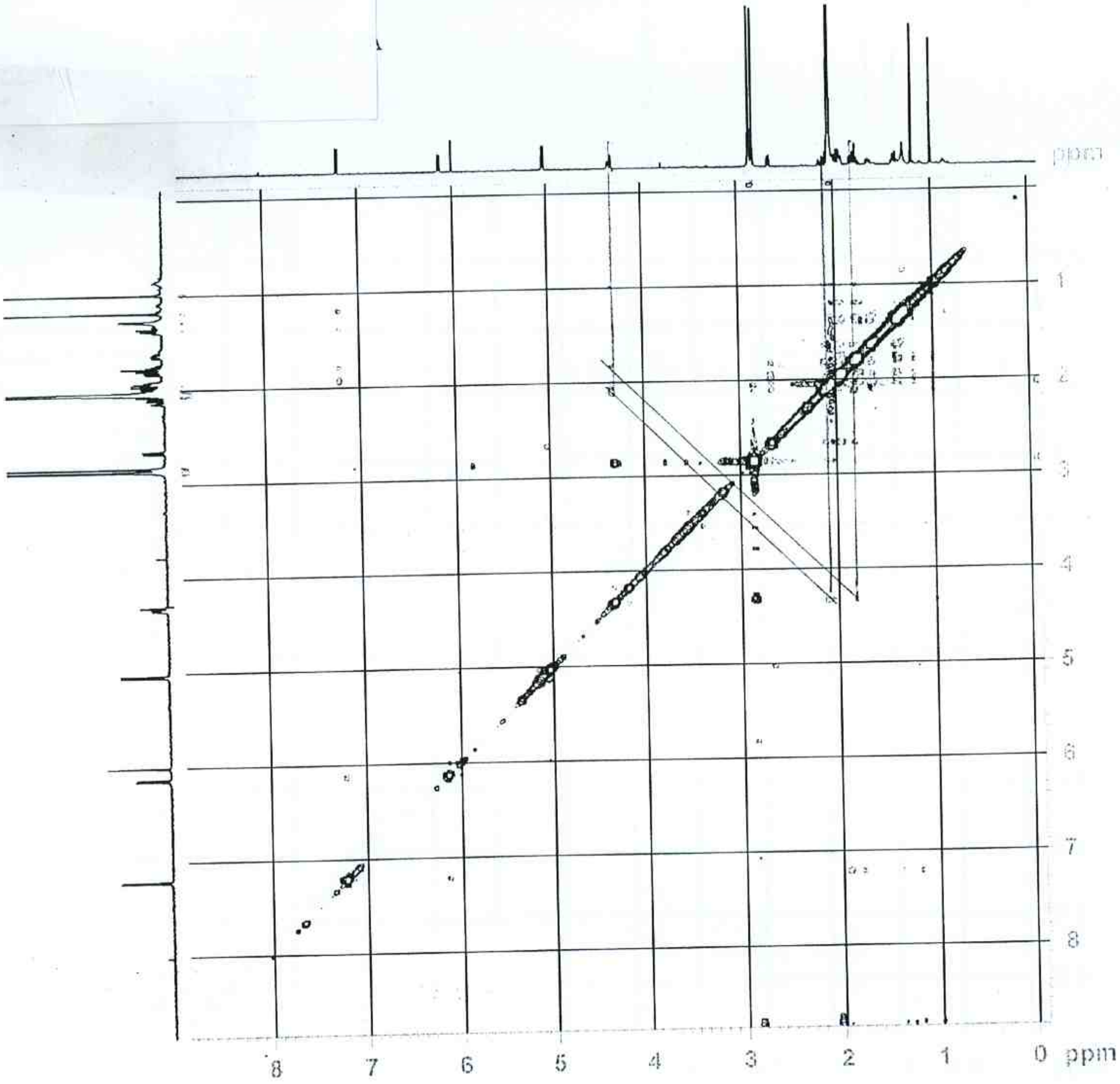

NAME	mar10-12
EXPNO	3
PROCNO	1
Date_	20120310
Time	13.30
INSTRUM	spect
PROBHD	5 mm CPTCI 1H-
PULPROG	noesygpph
TD	1024
SOLVENT	Acetone
NS	8
DS	4
SWH	5387.931 Hz
FIDRES	5.261652 Hz
AQ	0.0951700 sec
RG	181
DW	92.800 usec
DE	6.50 usec
TE	294.3 K
D0	0.00008338 sec
D1	1.50000000 sec
D8	0.80000001 sec
D16	0.00015000 sec
IN0	0.00018560 sec

======== CHANNEL f1 ========

NUC1	1H
P1	7.40 usec
P2	14.80 usec
PL1	3.30 dB
PL1W	9.16420078 W
SFO1	600.2326410 MHz

====== GRADIENT CHANNEL =====

GPNAM1	SINE.100
GPNAM2	SINE.100
GPZ1	40.00 %
GPZ2	-40.00 %
P16	2000.00 usec
ND0	1
TD	256
SFO1	600.2326 MHz
FIDRES	21.046606 Hz
SW	8.976 ppm
FnMODE	States-TPPI
SI	1024
SF	600.2300139 MHz
WDW	QSINE
SSB	2
LB	0.00 Hz
GB	0
PC	1.40
SI	1024
MC2	States-TPPI
SF	600.2300139 MHz
WDW	QSINE
SSB	2
LB	0.00 Hz
GB	0
