# Supplementary material for: Microbial transformation of anti-cancer steroid exemestane and cytotoxicity of its metabolites against cancer cell lines
Source: Chem Cent J. 2013 Mar 27;7:57. doi: 10.1186/1752-153X-7-57 (PMC3637571; doi:10.1186/1752-153X-7-57)
Supplement: Additional file 2 — Spectroscopic data of metabolite 3. Include spectra of EI-MS, UV, IR, 1H-NMR, 13C-NMR (BB, DEPT-135°, DEPT-90°), HSQC, HMBC, COSY-45°, NOESY experiments. [file 1752-153X-7-57-S2.pdf]

Ionization mode: EI+

Scan: 15
Base: m/z 148; 62.7%FS TIC: 18696040

R.T.: 1.25

#Ions: 316

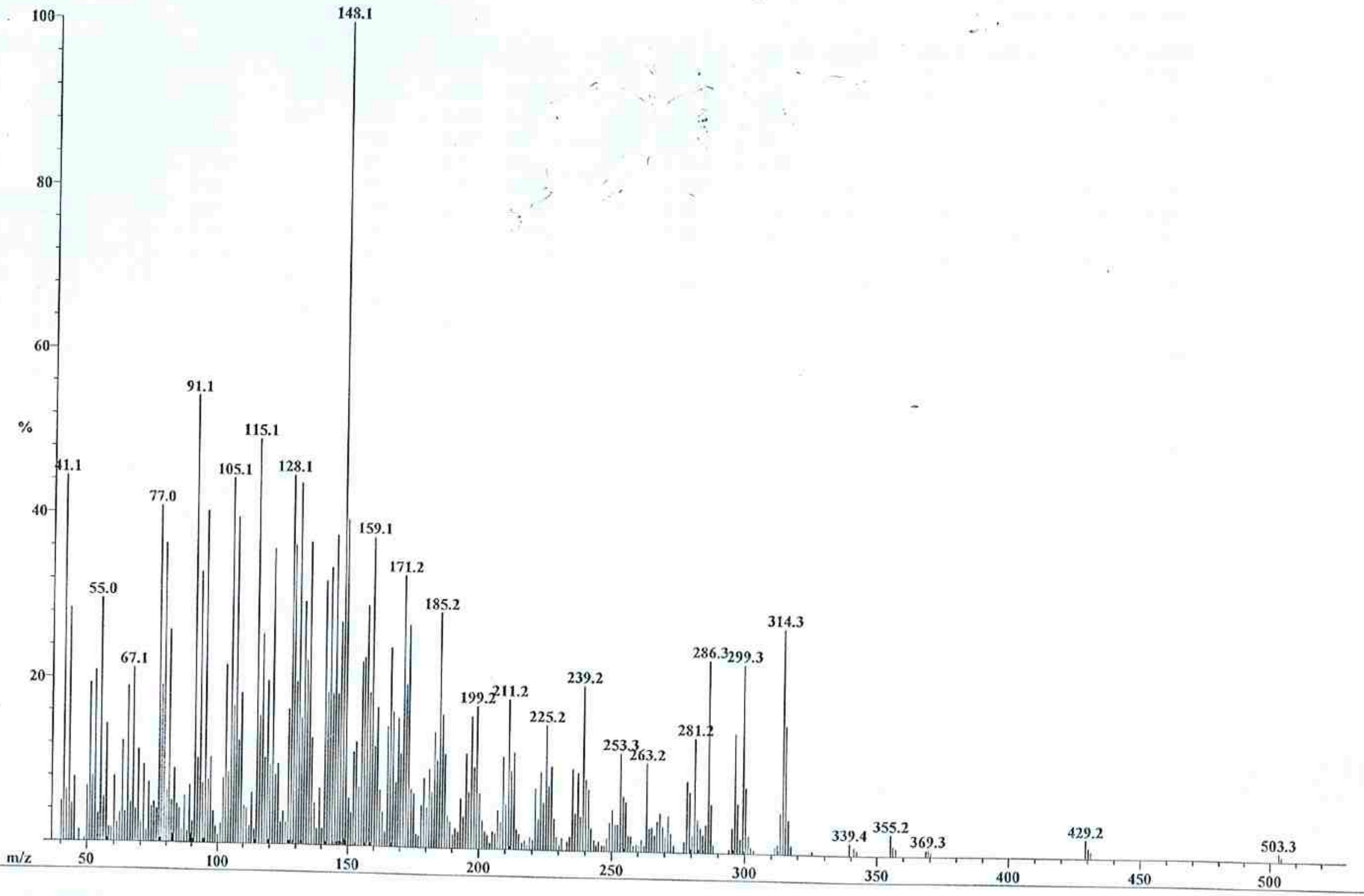

[illegible]

Scan Graph

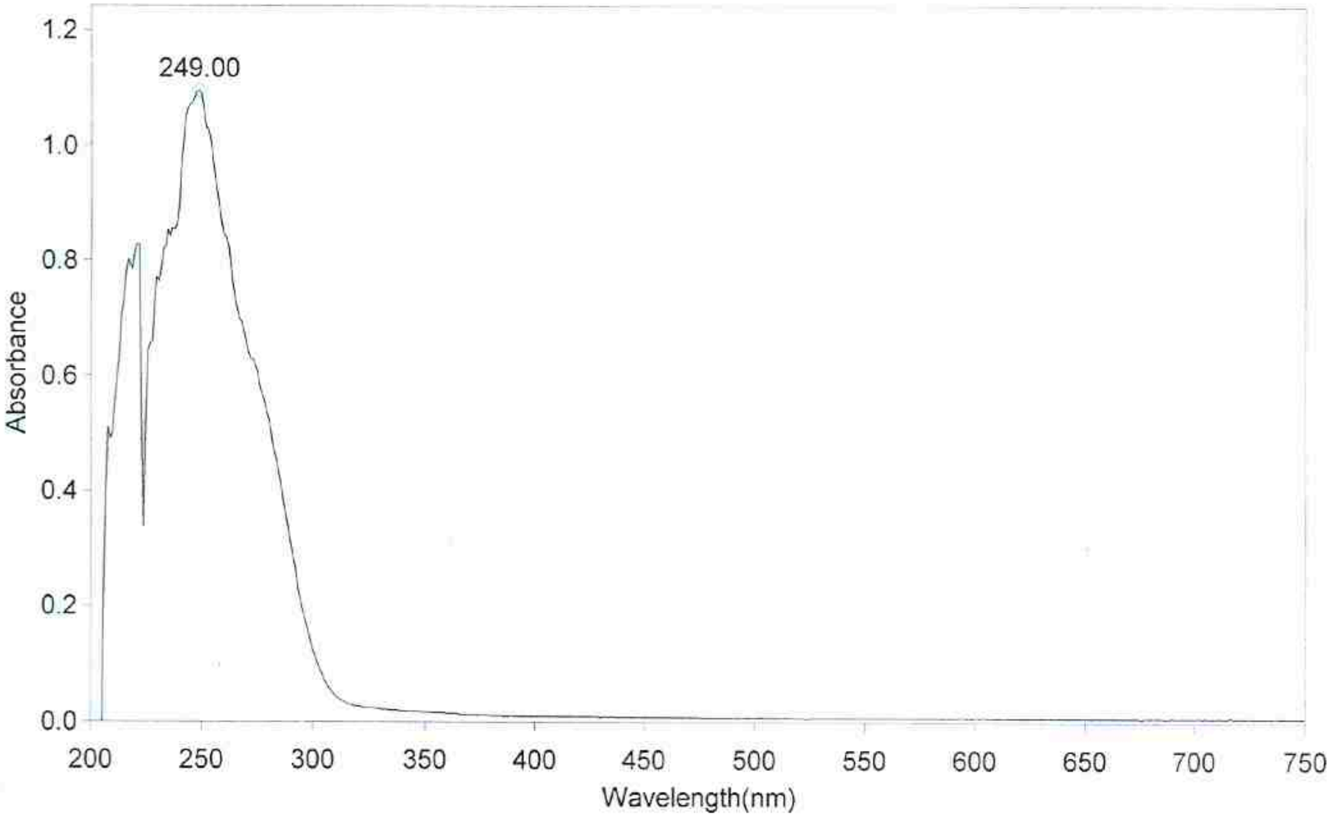

249.00
Absorbance
Wavelength(nm)
1.2
1.0
0.8
0.6
0.4
0.2
0.0
200
250
300
350
400
450
500
550
600
650
700
750

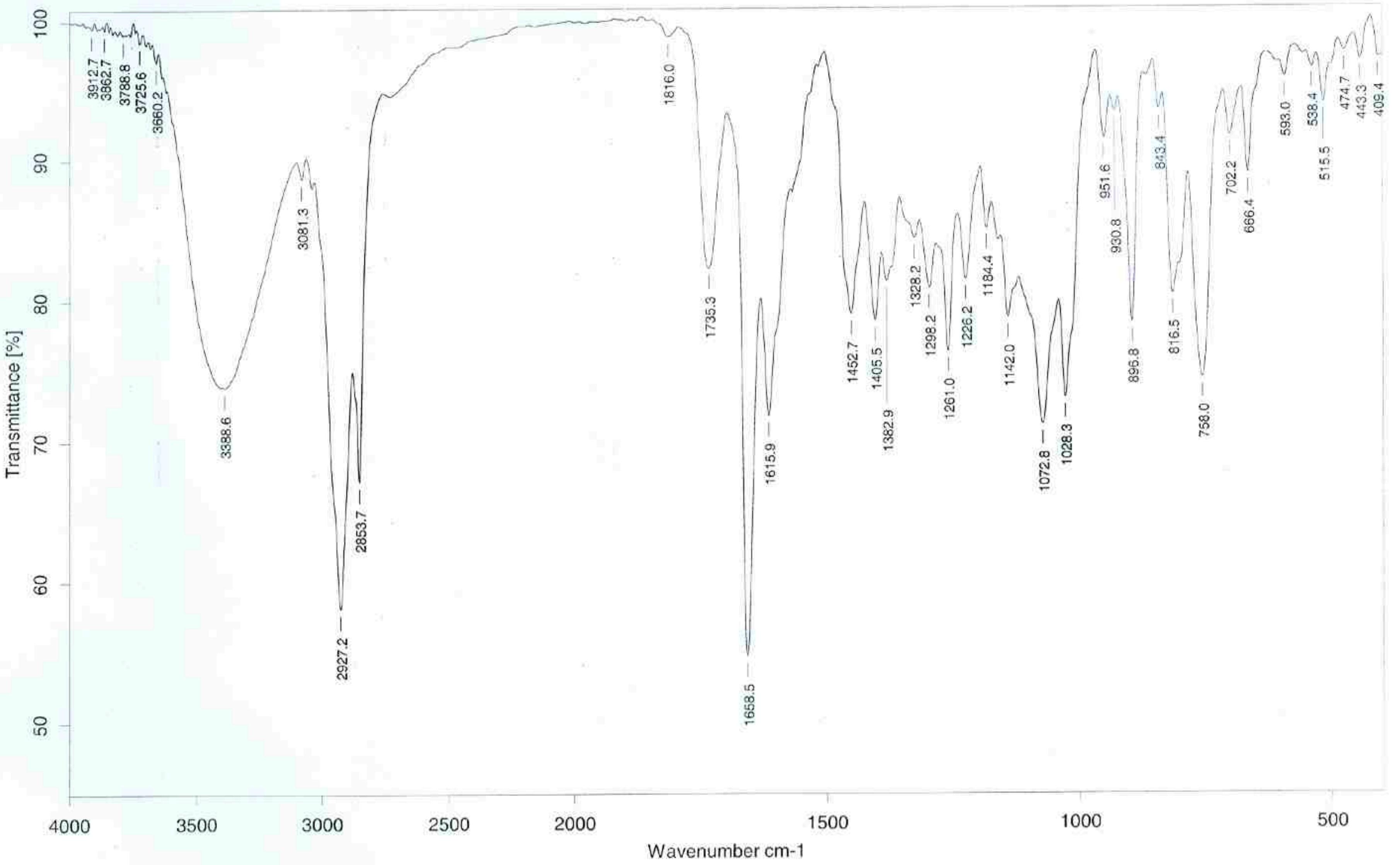
Transmittance [%]
Wavenumber cm-1
100
90
80
70
60
50
4000
3500
3000
2500
2000
1500
1000
500
3912.7
3862.7
3788.8
3725.6
3660.2
3388.6
3081.3
2927.2
2853.7
1816.0
1735.3
1658.5
1615.9
1452.7
1405.5
1382.9
1328.2
1298.2
1261.0
1226.2
1184.4
1142.0
1072.8
1028.3
951.6
930.8
896.8
843.4
816.5
758.0
702.2
666.4
593.0
538.4
515.5
474.7
443.3
409.4

LAB NO: 108

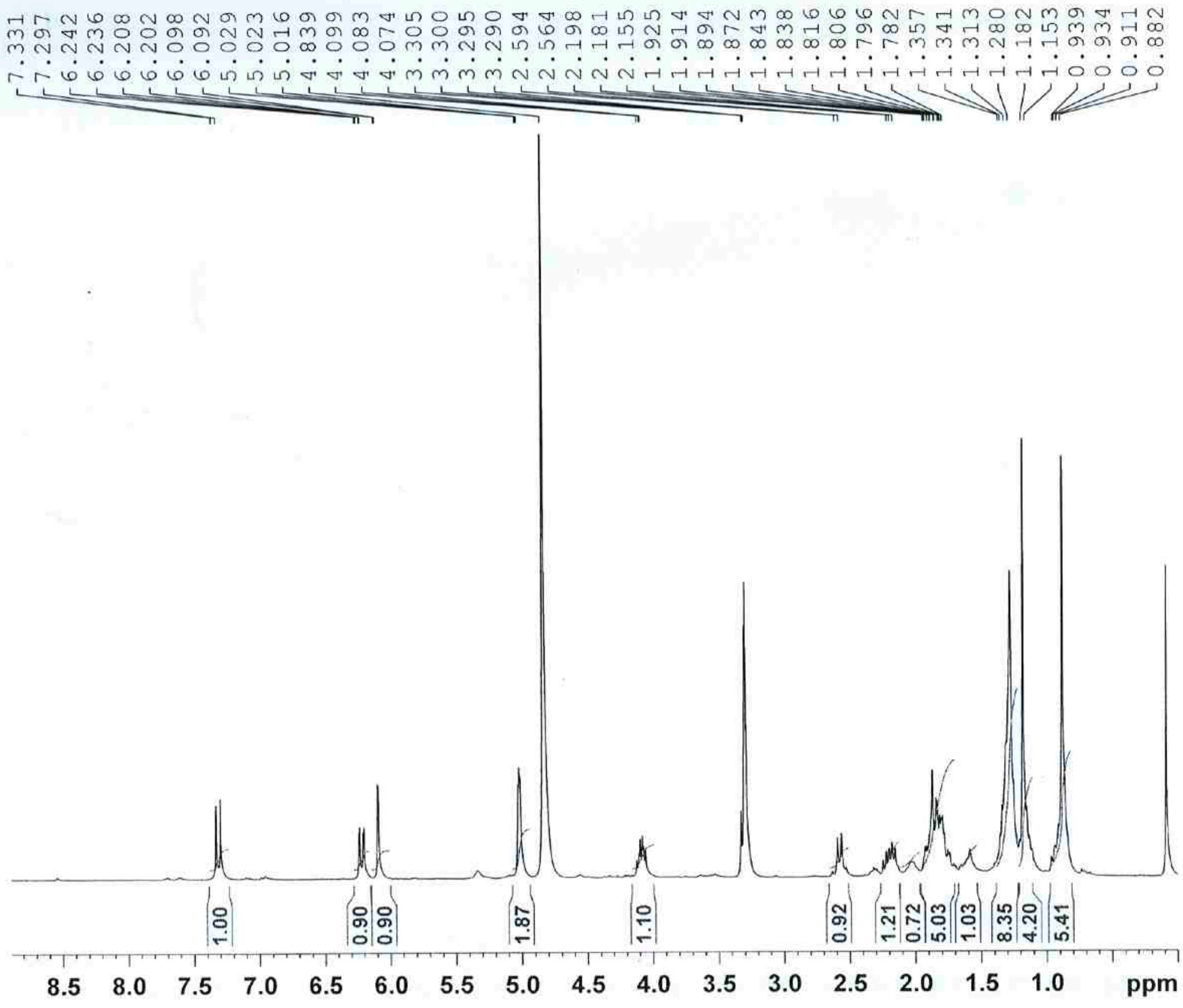

NAME	may15
EXPNO	3
PROCNO	1
Date_	20120515
Time_	10.51
INSTRUM	spect
PROBHD	5 mm DUL 13C-1
PULPROG	zg30
TD	32768
SOLVENT	MeOD
NS	32
DS	0
SWH	6172.839 Hz
FIDRES	0.188380 Hz
AQ	2.6542580 sec
RG	362
DW	81.000 usec
DE	6.50 usec
TE	298.1 K
D1	1.50000000 sec
TD0	1

======== CHANNEL f1 ========

NUC1	1H
P1	12.80 usec
PL1	5.00 dB
SFO1	300.1324010 MHz
SI	32768
SF	300.1300076 MHz
WDW	EM
SSB	0
LB	0.30 Hz
GB	0
PC	1.00

BB

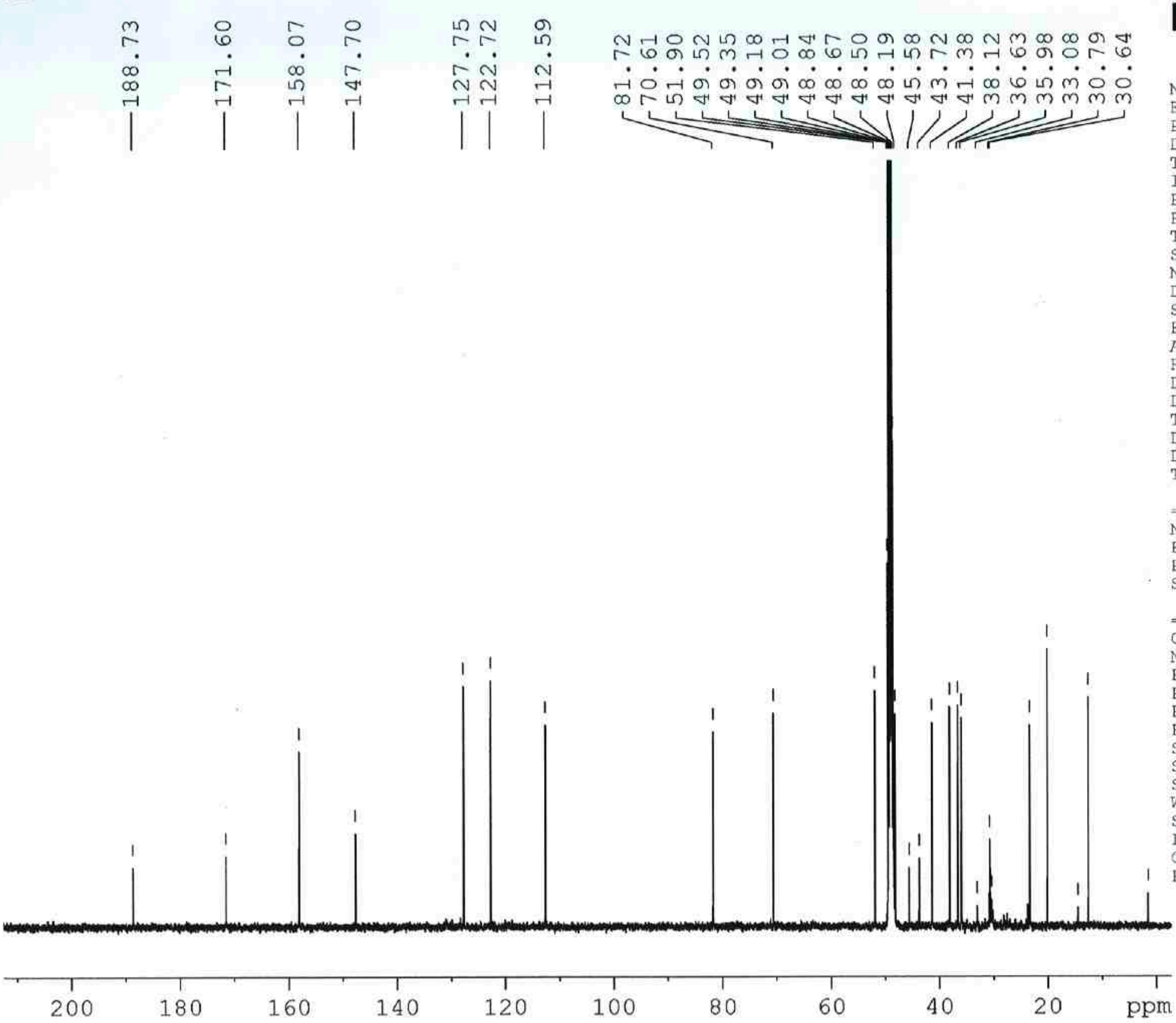

BRUKER

NAME	may28-12	
EXPNO	2	
PROCNO	1	
Date_	20120528	
Time	15.46	
INSTRUM	spect	
PROBHD	5 mm PABBI 1H/	
PULPROG	zgpg	
TD	32768	
SOLVENT	MeOD	
NS	20480	
DS	2	
SWH	30303.031	Hz
FIDRES	0.924775	Hz
AQ	0.5407385	sec
RG	32768	
DW	16.500	usec
DE	6.50	usec
TE	300.0	K
D1	1.50000000	sec
D11	0.03000000	sec
TD0	20	

======== CHANNEL f1 ========

NUC1	13C	
P1	15.25	usec
PL1	-3.00	dB
SFO1	125.7975248	MHz

======== CHANNEL f2 ========

CPDPRG2	waltz16	
NUC2	1H	
PCPD2	80.00	usec
PL2	5.75	dB
PL12	25.50	dB
PL13	120.00	dB
SFO2	500.2330014	MHz
SI	32768	
SF	125.7827561	MHz
WDW	EM	
SSB	0	
LB	1.00	Hz
GB	0	
PC	1.40	

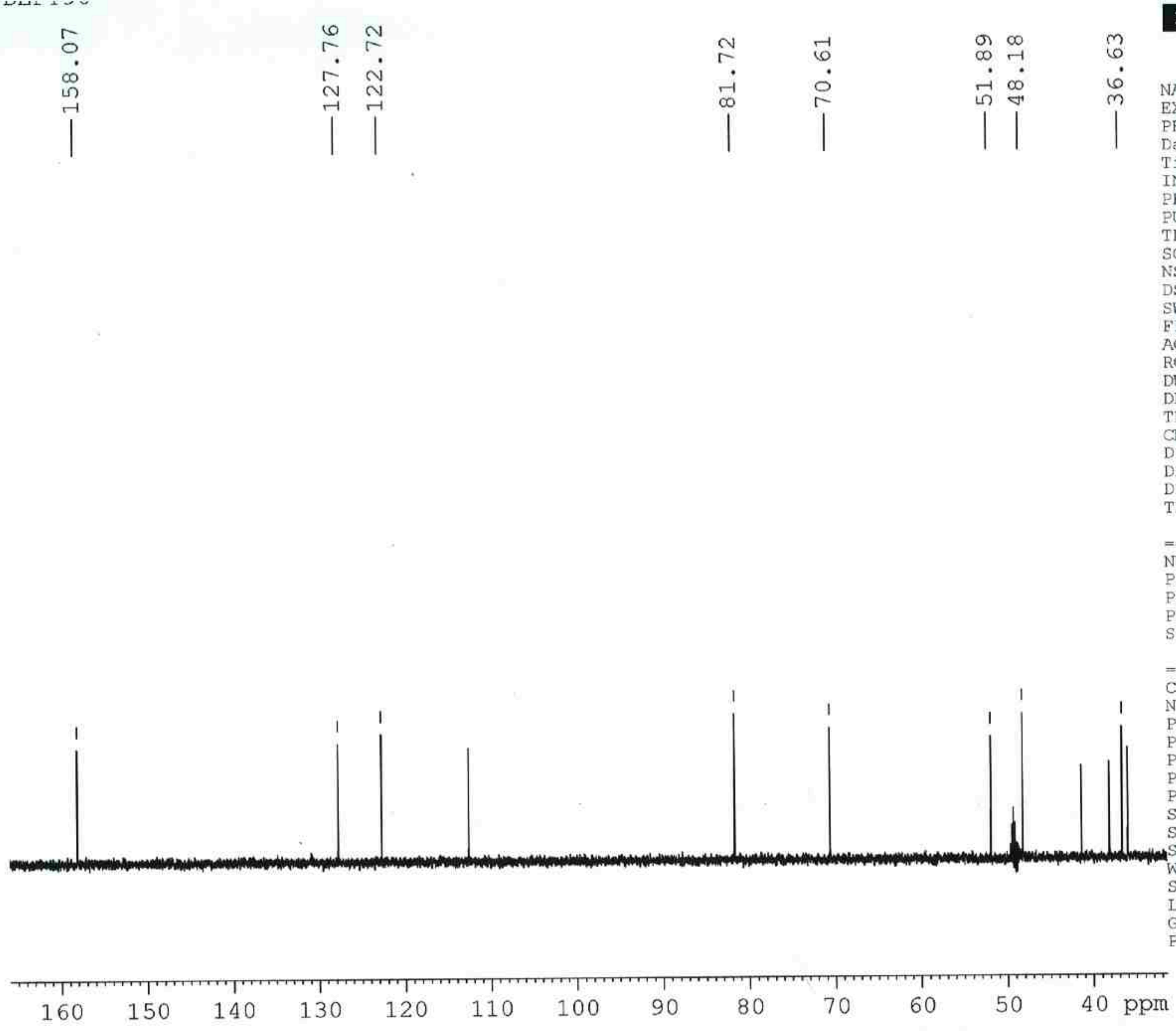

BRUKER

NAME	may28-12
EXPNO	4
PROCNO	1
Date_	20120529
Time	9.43
INSTRUM	spect
PROBHD	5 mm PABBI 1H/
PULPROG	dept90
TD	32768
SOLVENT	MeOD
NS	2398
DS	0
SWH	23809.523 Hz
FIDRES	0.726609 Hz
AQ	0.6881990 sec
RG	32768
DW	21.000 usec
DE	6.50 usec
TE	299.7 K
CNST2	145.0000000
D1	1.50000000 sec
D2	0.00344828 sec
D12	0.00002000 sec
TD0	6

======== CHANNEL f1 ========

NUC1	13C
P1	15.25 usec
P2	30.50 usec
PL1	-3.00 dB
SFO1	125.7948834 MHz

======== CHANNEL f2 ========

CPDPRG2	waltz16
NUC2	1H
P3	8.00 usec
P4	16.00 usec
PCPD2	80.00 usec
PL2	5.75 dB
PL12	25.50 dB
SFO2	500.2330014 MHz
SI	32768
SF	125.7827561 MHz
WDW	EM
SSB	0
LB	1.00 Hz
GB	0
PC	1.40

DEPT135

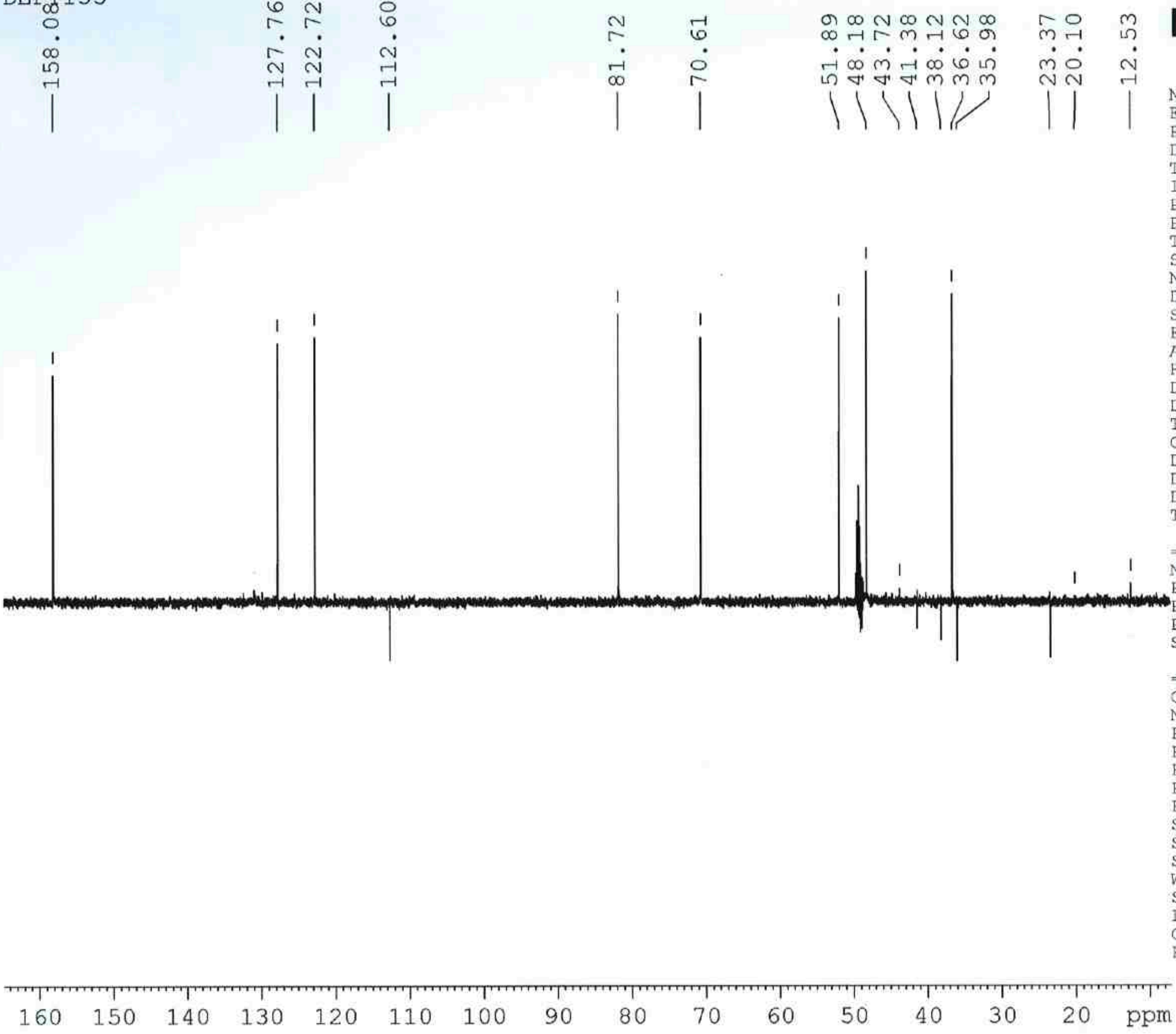

BRUKER

NAME	may28-12
EXPNO	3
PROCNO	1
Date_	20120529
Time	3.46
INSTRUM	spect
PROBHD	5 mm PABBI 1H/
PULPROG	dept135
TD	32768
SOLVENT	MeOD
NS	10240
DS	0
SWH	23809.523 Hz
FIDRES	0.726609 Hz
AQ	0.6881990 sec
RG	32768
DW	21.000 usec
DE	6.50 usec
TE	298.9 K
CNST2	145.0000000
D1	1.50000000 sec
D2	0.00344828 sec
D12	0.00002000 sec
TD0	10

======== CHANNEL f1 ========

NUC1	13C
P1	15.25 usec
P2	30.50 usec
PL1	-3.00 dB
SFO1	125.7948834 MHz

======== CHANNEL f2 ========

CPDPRG2	waltz16
NUC2	1H
P3	8.00 usec
P4	16.00 usec
PCPD2	80.00 usec
PL2	5.75 dB
PL12	25.50 dB
SFO2	500.2330014 MHz
SI	32768
SF	125.7827561 MHz
WDW	EM
SSB	0
LB	1.00 Hz
GB	0
PC	1.20

BRUKER

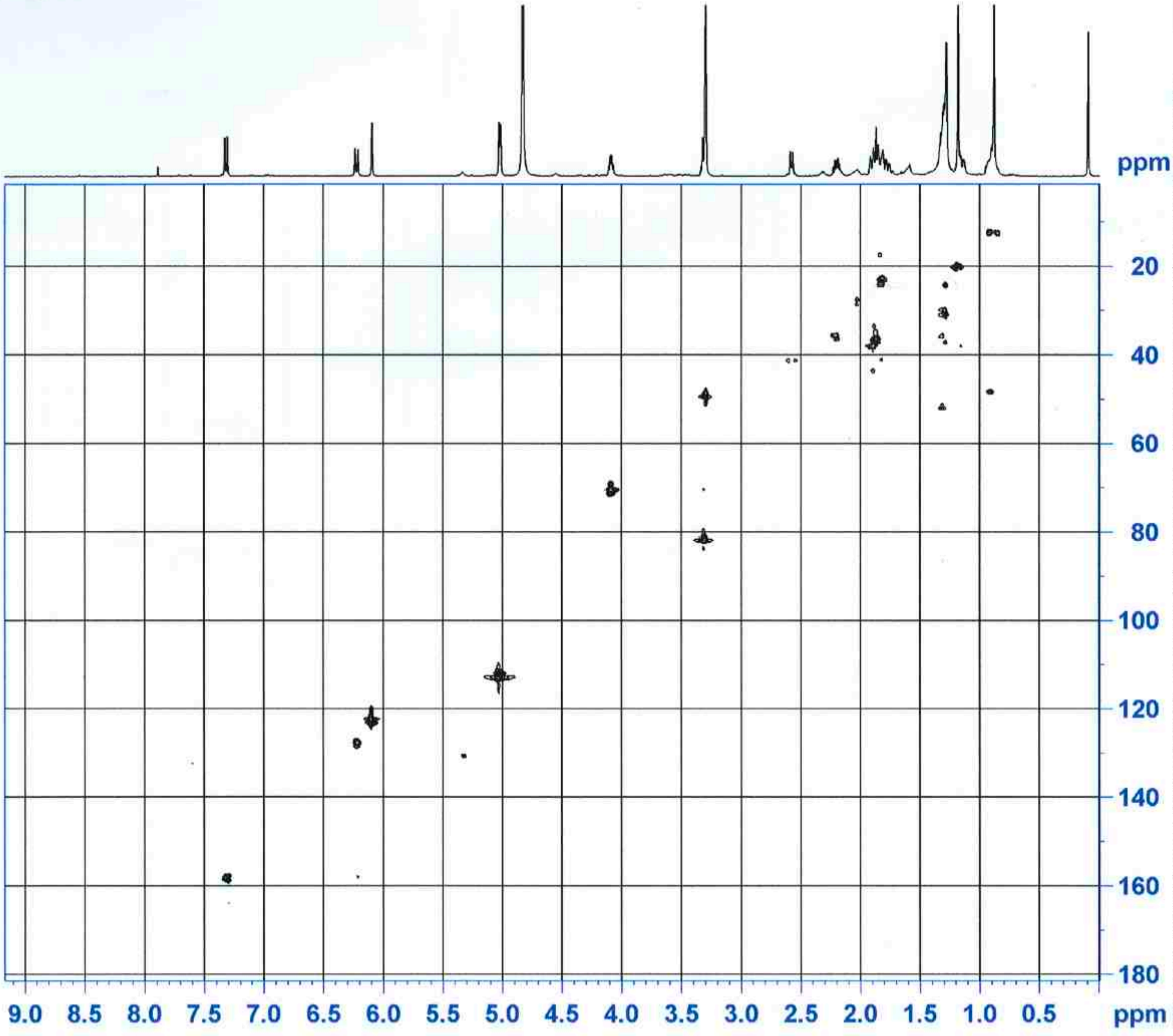

NAME	may29-12
EXPNO	2
PROCNO	1
Date_	20120529
Time	11.36
INSTRUM	spect
PROBHD	5 mm PABBI 1H/
PULPROG	hsqcetgpsi
TD	1024
SOLVENT	MeOD
NS	32
DS	8
SWH	4595.588 Hz
FIDRES	4.487879 Hz
AQ	0.1115700 sec
RG	20642.5
DW	108.800 usec
DE	6.50 usec
TE	300.0 K
CNST2	145.0000000
D0	0.00000300 sec
D1	1.50000000 sec
D4	0.00172414 sec
D11	0.03000000 sec
D13	0.00000400 sec
D16	0.00020000 sec
D24	0.00110000 sec
IN0	0.00002210 sec
ZGOPTNS	

======== CHANNEL f1 ========

NUC1	1H
P1	7.05 usec
P2	14.10 usec
P28	1000.00 usec
PL1	5.00 dB
SFO1	500.2323011 MHz

======== CHANNEL f2 ========

CPDPRG2	garp
NUC2	13C
P3	15.25 usec
P4	30.50 usec
PCPD2	70.00 usec
PL2	-3.00 dB
PL12	9.00 dB
SFO2	125.7942545 MHz

====== GRADIENT CHANNEL =====

GPNAM1	SINE.100
GPNAM2	SINE.100
GPZ1	80.00 %
GPZ2	20.10 %
P16	1000.00 usec
ND0	2
TD	256
SFO1	125.7943 MHz
FIDRES	88.449081 Hz
SW	180.000 ppm
FnMODE	Echo-Antiecho
SI	1024
SF	500.2300121 MHz
WDW	QSINE
SSB	2
LB	0.00 Hz
GB	0
PC	1.00
SI	1024
MC2	echo-antiecho
SF	125.7827561 MHz
WDW	QSINE
SSB	2
LB	0.00 Hz
GB	0

LAB N

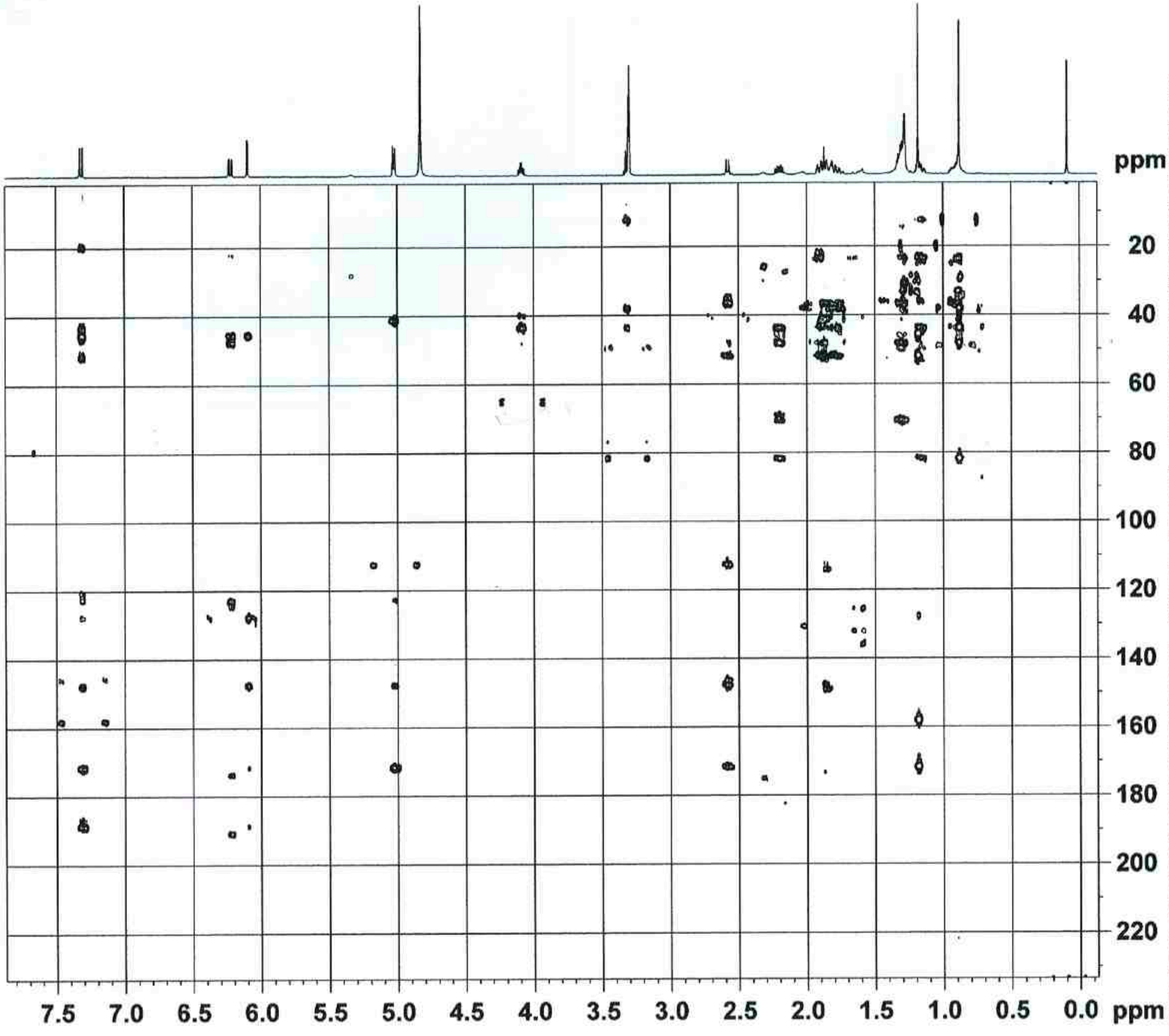

NAME	may19-12
EXPNO	13
PROCNO	1
Date_	20120519
Time	21.29
INSTRUM	spect
PROBHD	5 mm BBI 1H/D-
PULPROG	hmbcgplpndqf
TD	4096
SOLVENT	MeOD
NS	64
DS	8
SWH	4006.410 Hz
FIDRES	0.978127 Hz
AQ	0.5113556 sec
RG	23170.5
DW	124.800 usec
DE	6.50 usec
TE	302.0 K
CNST2	145.0000000
CNST13	10.0000000
D0	0.00000300 sec
D1	2.00000000 sec
D2	0.00344828 sec
D6	0.05000000 sec
D16	0.00010000 sec
IN0	0.00001715 sec
======== CHANNEL f1 ========	
NUC1	1H
P1	7.60 usec
P2	15.20 usec
PL1	6.00 dB
SFO1	500.1319505 MHz
======== CHANNEL f2 ========	
NUC2	13C
P3	13.25 usec
PL2	-2.85 dB
SFO2	125.7723769 MHz
====== GRADIENT CHANNEL =====	
GPNAM1	SINE.100
GPNAM2	SINE.100
GPNAM3	SINE.100
GPZ1	50.00 %
GPZ2	30.00 %
GPZ3	40.10 %
P16	1000.00 usec
ND0	2
TD	256
SFO1	125.7724 MHz
FIDRES	113.981216 Hz
SW	232.000 ppm
FnMODE	QF
SI	1024
SF	500.1300158 MHz
WDW	QSINE
SSB	0
LB	0.00 Hz
GB	0
PC	4.00
SI	1024
MC2	QF
SF	125.7576112 MHz
WDW	QSINE
SSB	0
LB	0.00 Hz
GB	0

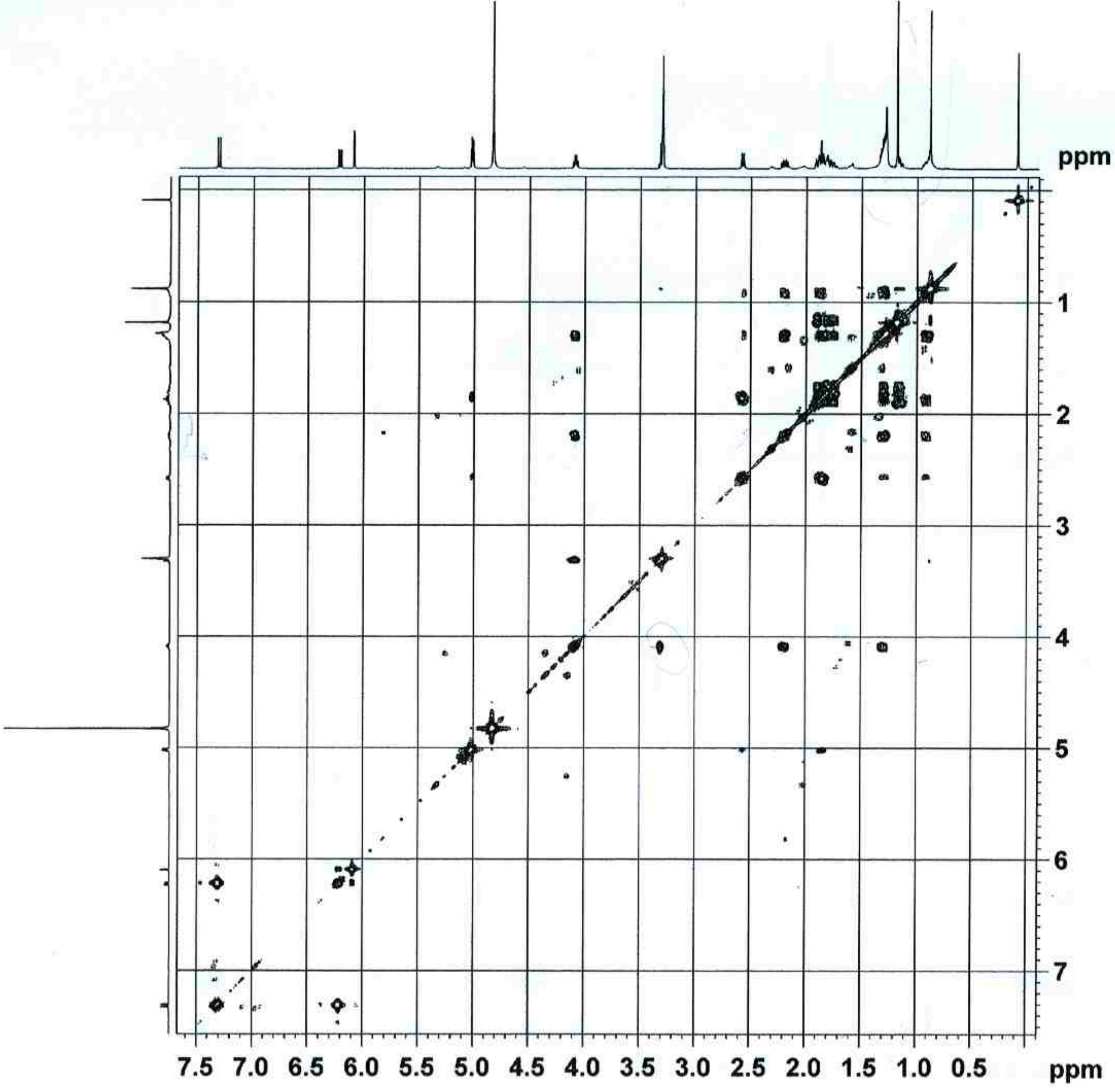

NAME	may19-12
EXPNO	10
PROCNO	1
Date_	20120519
Time	13.44
INSTRUM	spect
PROBHD	5 mm BBI 1H/D-
PULPROG	cosygpqf
TD	2048
SOLVENT	MeOD
NS	8
DS	8
SWH	4006.410 Hz
FIDRES	1.956255 Hz
AQ	0.2557652 sec
RG	256
DW	124.800 usec
DE	6.50 usec
TE	299.8 K
D0	0.00000300 sec
D1	1.50000000 sec
D13	0.00000400 sec
D16	0.00010000 sec
IN0	0.00024960 sec

======== CHANNEL f1 ========

NUC1	1H
P0	7.60 usec
P1	7.60 usec
PL1	6.00 dB
SFO1	500.1319505 MHz

====== GRADIENT CHANNEL =====

GPNAM1	SINE.100
GPZ1	10.00 %
P16	1000.00 usec
ND0	1
TD	256
SFO1	500.132 MHz
FIDRES	15.650039 Hz
SW	8.011 ppm
FnMODE	QF
SI	1024
SF	500.1300158 MHz
WDW	SINE
SSB	0
LB	0.00 Hz
GB	0
PC	4.00
SI	1024
MC2	QF
SF	500.1300158 MHz
WDW	SINE
SSB	0
LB	0.00 Hz
GB	0

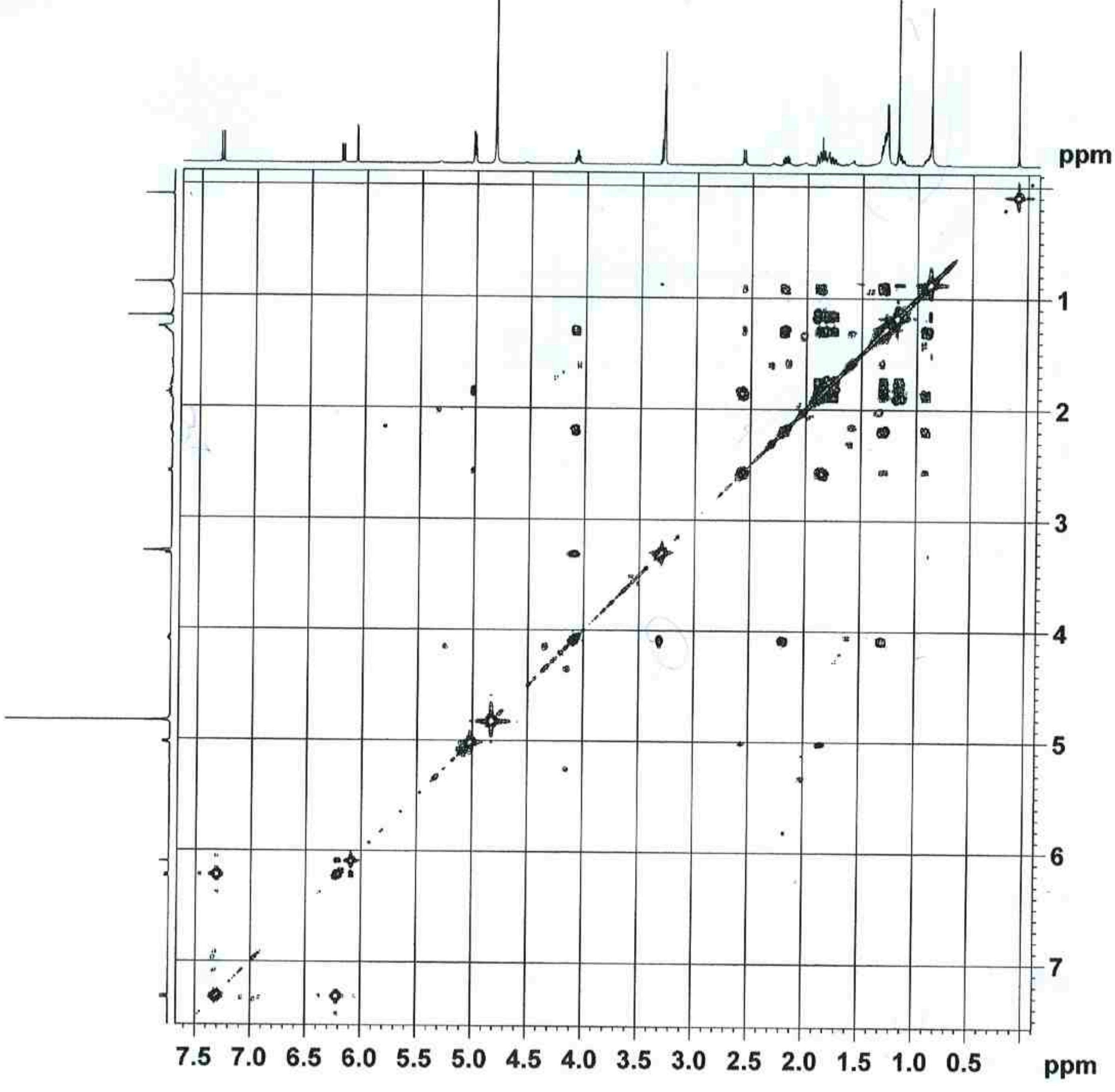

NAME	may19-12
EXPNO	10
PROCNO	1
Date_	20120519
Time	13.44
INSTRUM	spect
PROBHD	5 mm BBI 1H/D-
PULPROG	cosygpqf
TD	2048
SOLVENT	MeOD
NS	8
DS	8
SWH	4006.410 Hz
FIDRES	1.956255 Hz
AQ	0.2557652 sec
RG	256
DW	124.800 usec
DE	6.50 usec
TE	299.8 K
D0	0.00000300 sec
D1	1.50000000 sec
D13	0.00000400 sec
D16	0.00010000 sec
IN0	0.00024960 sec

======== CHANNEL f1 ========

NUC1	1H
P0	7.60 usec
P1	7.60 usec
PL1	6.00 dB
SFO1	500.1319505 MHz

====== GRADIENT CHANNEL =====

GPNAM1	SINE.100
GPZ1	10.00 %
P16	1000.00 usec
ND0	1
TD	256
SFO1	500.132 MHz
FIDRES	15.650039 Hz
SW	8.011 ppm
FnMODE	QF
SI	1024
SF	500.1300158 MHz
WDW	SINE
SSB	0
LB	0.00 Hz
GB	0
PC	4.00
SI	1024
MC2	QF
SF	500.1300158 MHz
WDW	SINE
SSB	0
LB	0.00 Hz
GB	0
