# Supplementary material for: Microbial transformation of anti-cancer steroid exemestane and cytotoxicity of its metabolites against cancer cell lines
Source: Chem Cent J. 2013 Mar 27;7:57. doi: 10.1186/1752-153X-7-57 (PMC3637571; doi:10.1186/1752-153X-7-57)
Supplement: Additional file 3 — Spectroscopic data of metabolite 4. Include spectra of EI-MS, UV, IR, 1H-NMR, 13C-NMR (BB, DEPT-135°, DEPT-90°), HMQC, HMBC, COSY-45°, NOESY, experiments. [file 1752-153X-7-57-S3.pdf]

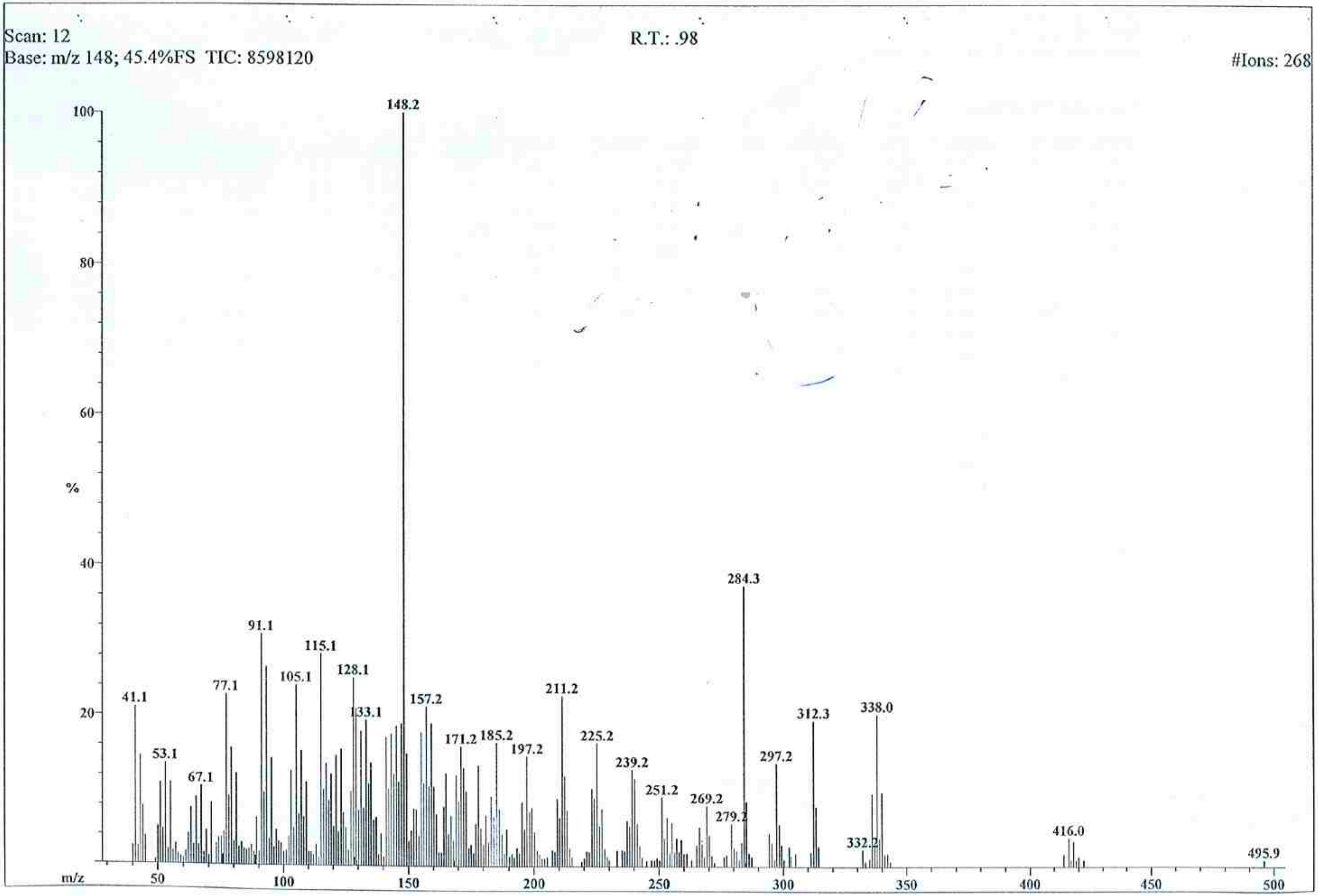
Scan: 12
Base: m/z 148; 45.4%FS TIC: 8598120
R.T.: .98
#Ions: 268
%
m/z
148.2
91.1
115.1
105.1
128.1
77.1
41.1
133.1
157.2
53.1
67.1
171.2
185.2
197.2
211.2
225.2
239.2
251.2
269.2
279.2
284.3
297.2
312.3
338.0
332.2
416.0
495.9

Scan Graph

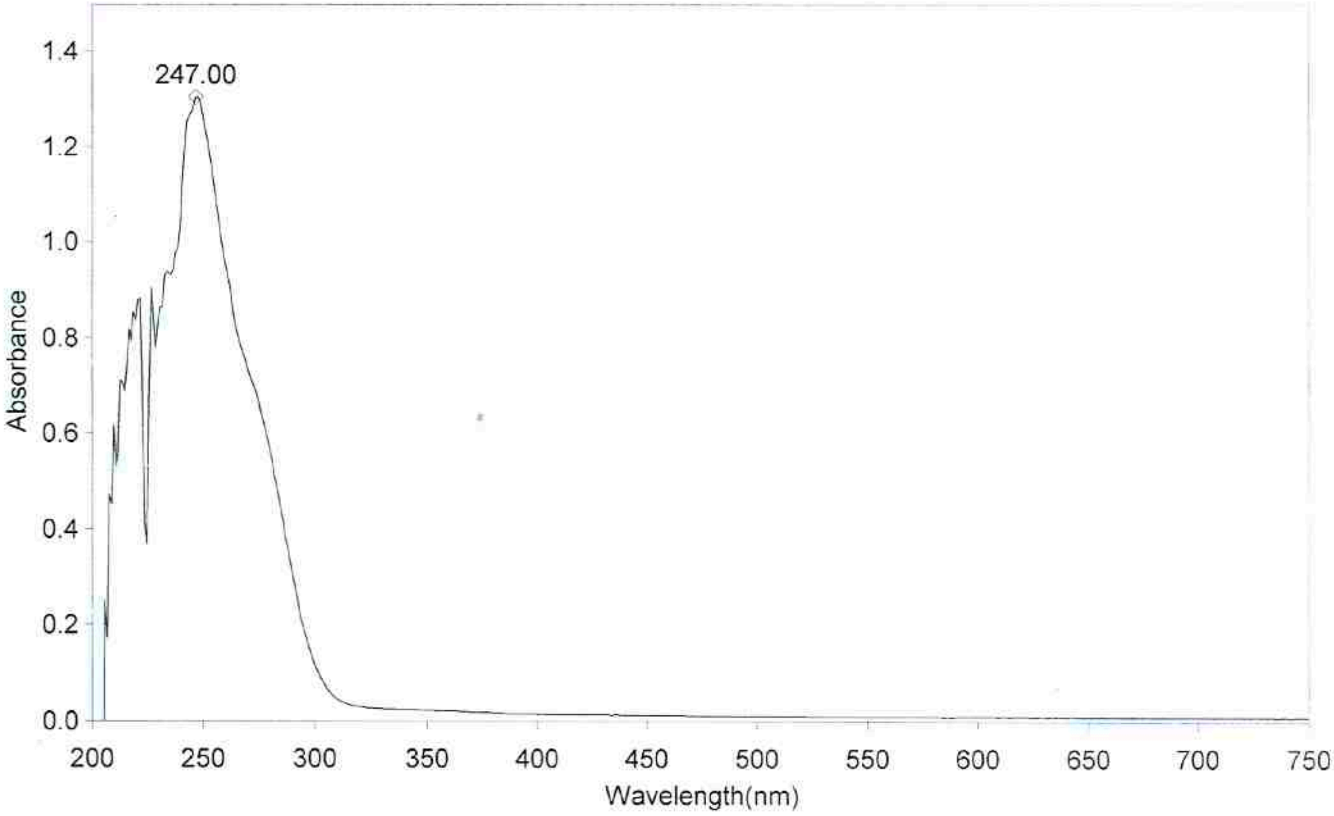
247.00
Absorbance
0.0
0.2
0.4
0.6
0.8
1.0
1.2
1.4
200
250
300
350
400
450
500
550
600
650
700
750
Wavelength(nm)

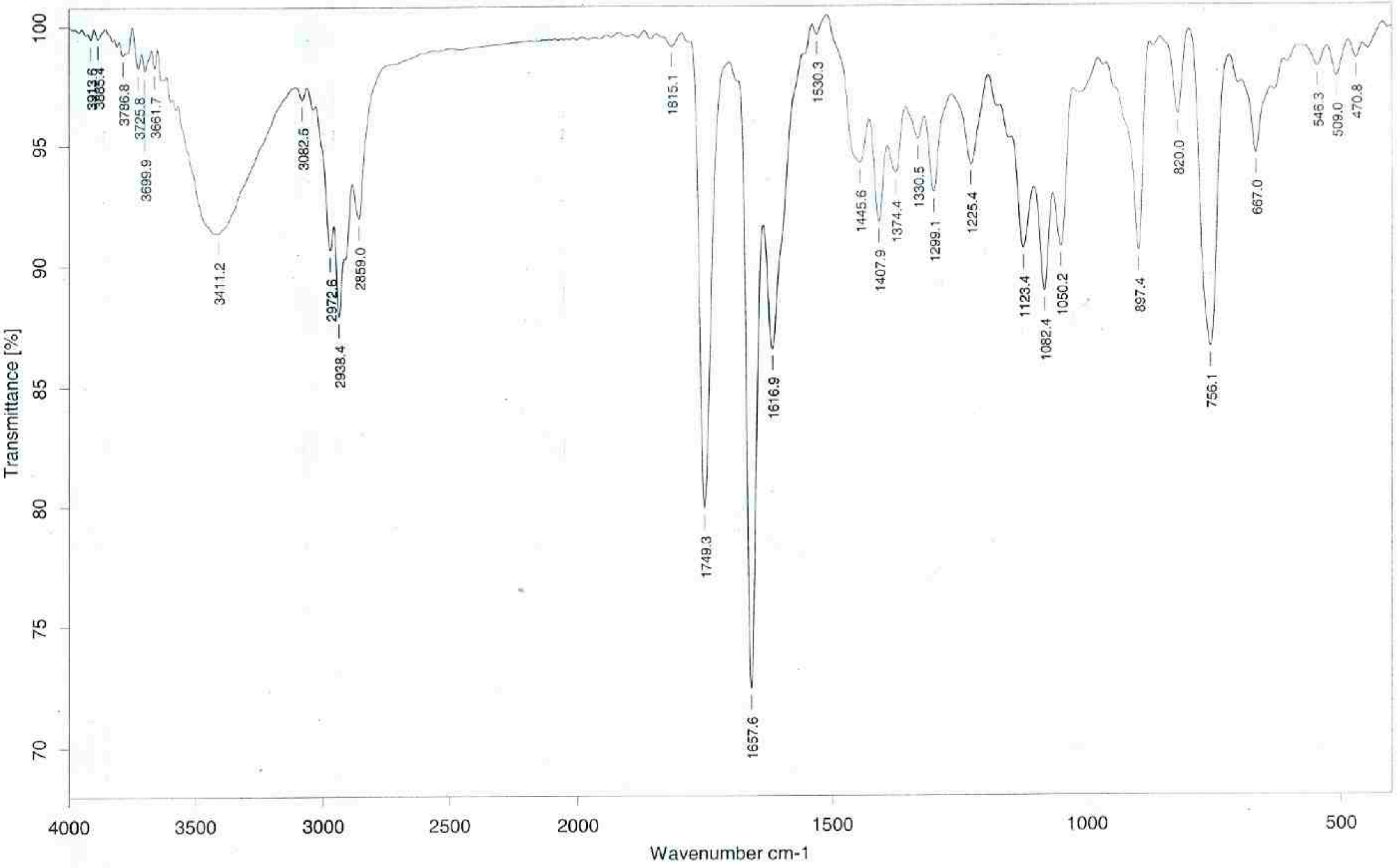
Transmittance [%]
Wavenumber cm-1
100
95
90
85
80
75
70
4000
3500
3000
2500
2000
1500
1000
500
3913.6
3885.4
3786.8
3725.8
3699.9
3661.7
3411.2
3082.5
2972.6
2938.4
2859.0
1815.1
1749.3
1657.6
1616.9
1530.3
1445.6
1407.9
1374.4
1330.5
1299.1
1225.4
1123.4
1082.4
1050.2
897.4
820.0
756.1
667.0
546.3
509.0
470.8

[illegible]

LAB NO: 108

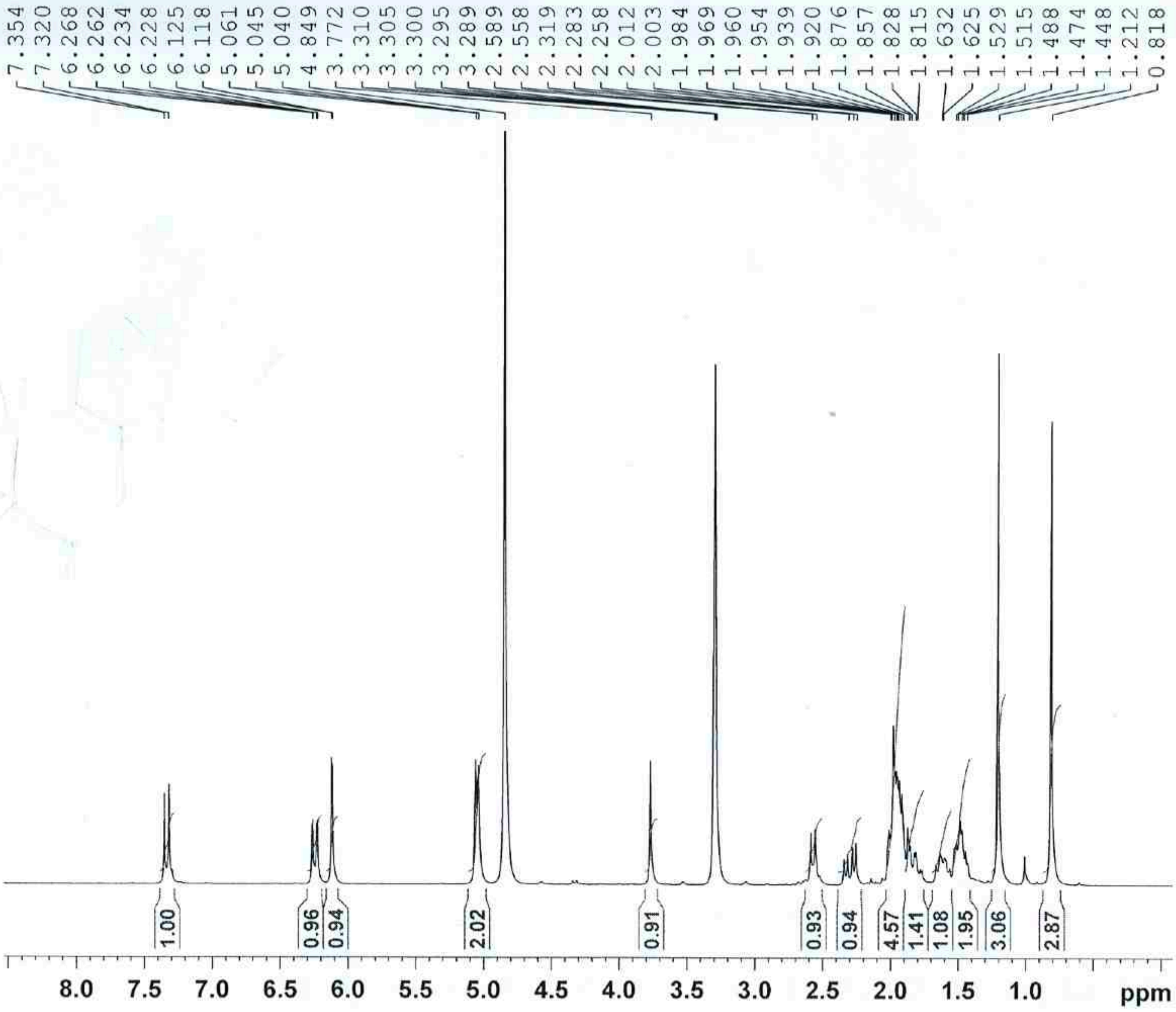

NAME	may18
EXPNO	2
PROCNO	1
Date_	20120518
Time	11.34
INSTRUM	spect
PROBHD	5 mm DUL 13C-1
PULPROG	zg30
TD	32768
SOLVENT	MeOD
NS	64
DS	0
SWH	6172.839 Hz
FIDRES	0.188380 Hz
AQ	2.6542580 sec
RG	512
DW	81.000 usec
DE	6.50 usec
TE	296.7 K
D1	1.50000000 sec
TD0	1

======== CHANNEL f1 ========

NUC1	1H
P1	12.80 usec
PL1	5.00 dB
SFO1	300.1324010 MHz
SI	32768
SF	300.1300076 MHz
WDW	EM
SSB	0
LB	0.30 Hz
GB	0
PC	1.00

M.Asif / Dr. Iqbal / Arc-1-2-10
BB

LAB NO. 116

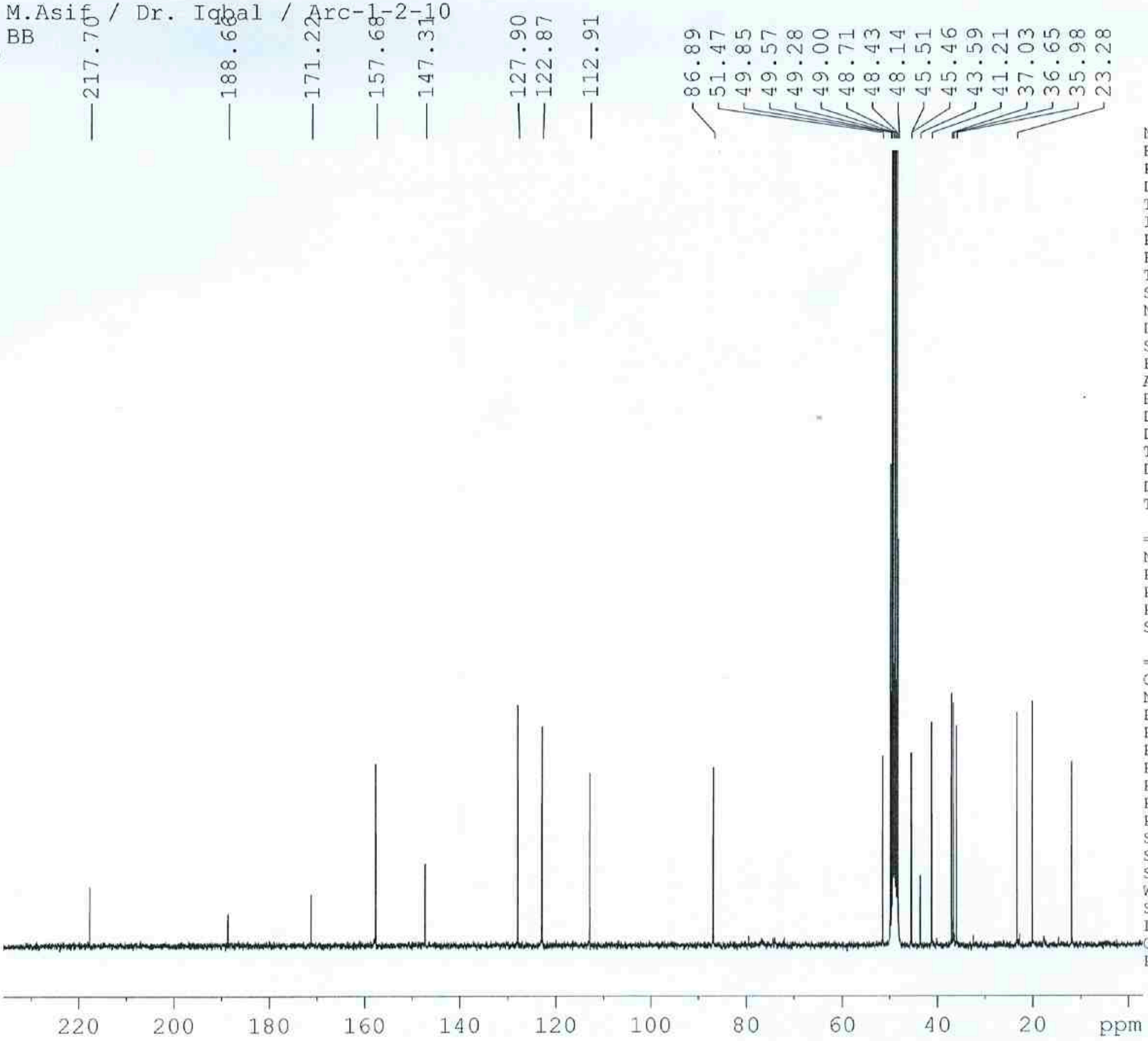

NAME	jun08	
EXPNO	1	
PROCNO	1	
Date_	20120608	
Time	12.51	
INSTRUM	Spect	
PROBHD	5 mm BBO BB-1H	
PULPROG	zgpg	
TD	32768	
SOLVENT	MeOD	
NS	18432	
DS	2	
SWH	18028.846	Hz
FIDRES	0.550197	Hz
AQ	0.9088159	sec
RG	22.6	
DW	27.733	usec
DE	6.50	usec
TE	300.0	K
D1	1.50000000	sec
D11	0.03000000	sec
TD0	18	
======== CHANNEL f1 ========		
NUC1	13C	
P1	11.00	usec
PL1	0.00	dB
PL1W	28.88669395	W
SFO1	75.4764278	MHz
======== CHANNEL f2 ========		
CPDPRG2	waltz16	
NUC2	1H	
PCPD2	80.00	usec
PL2	120.00	dB
PL12	17.00	dB
PL13	17.00	dB
PL2W	0.00000000	W
PL12W	0.26262212	W
PL13W	0.26262212	W
SFO2	300.1315007	MHz
SI	16384	
SF	75.4676422	MHz
WDW	EM	
SSB	0	
LB	1.00	Hz
GB	0	
PC	1.40	

DEPT90

LAB NO. 116

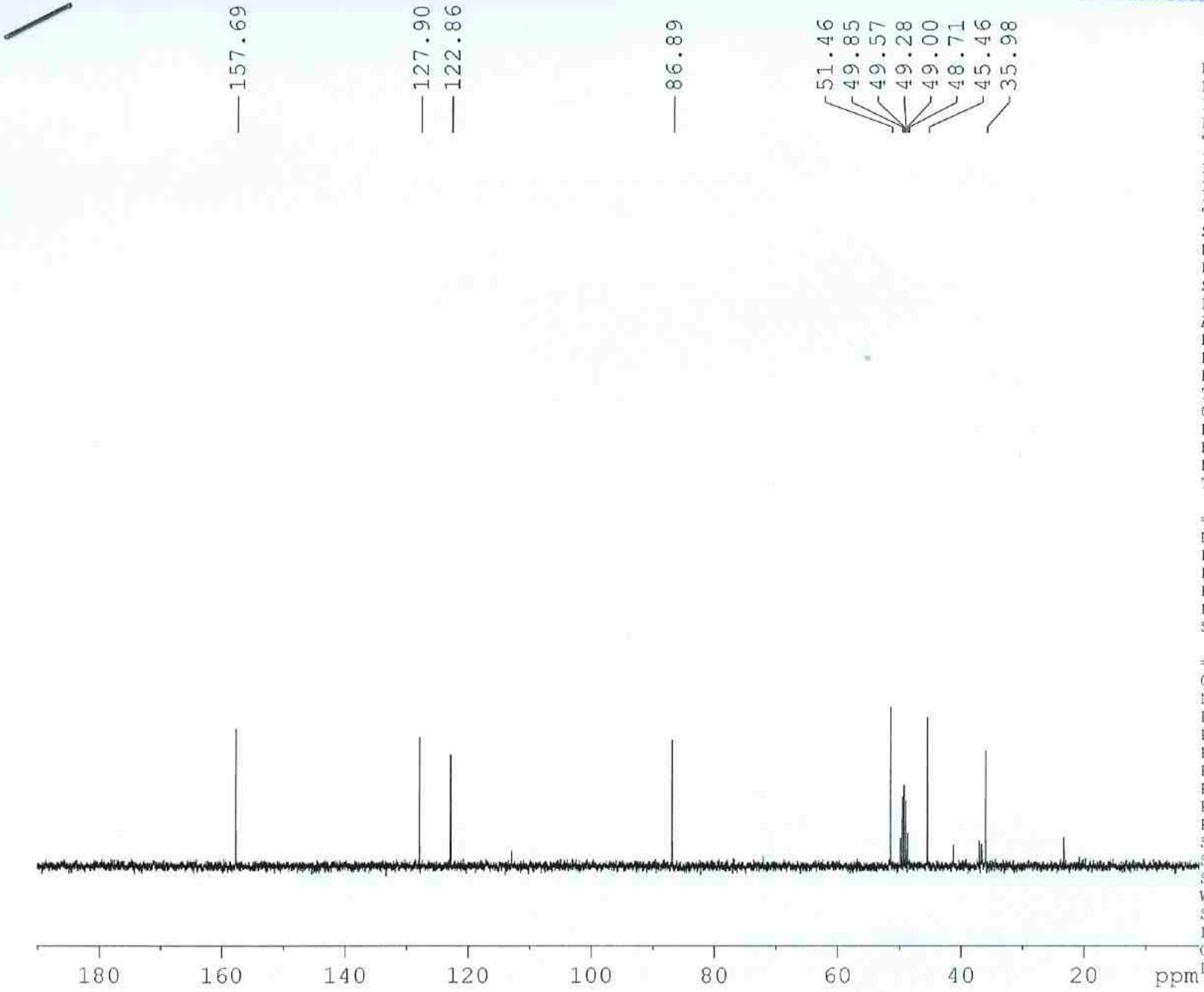

NAME	jun08
EXPNO	3
PROCNO	1
Date_	20120609
Time	7.41
INSTRUM	Spect
PROBHD	5 mm BBO BB-1H
PULPROG	dept90
TD	32768
SOLVENT	MeOD
NS	2528
DS	2
SWH	14367.816 Hz
FIDRES	0.438471 Hz
AQ	1.1403764 sec
RG	203
DW	34.800 usec
DE	6.50 usec
TE	300.0 K
CNST2	145.0000000
D1	1.50000000 sec
D2	0.00344828 sec
D12	0.00002000 sec
TD0	4
======== CHANNEL f1 ========	
NUC1	13C
P1	11.00 usec
P2	22.00 usec
PL1	0.00 dB
PL1W	28.88669395 W
SFO1	75.4749184 MHz
======== CHANNEL f2 ========	
CPDPRG2	waltz16
NUC2	1H
P3	12.00 usec
P4	24.00 usec
PCPD2	80.00 usec
PL2	0.00 dB
PL12	17.00 dB
PL2W	13.16228485 W
PL12W	0.26262212 W
SFO2	300.1315007 MHz
SI	16384
SF	75.4676422 MHz
WDW	EM
SSB	0
LB	1.00 Hz
GB	0
PC	1.00

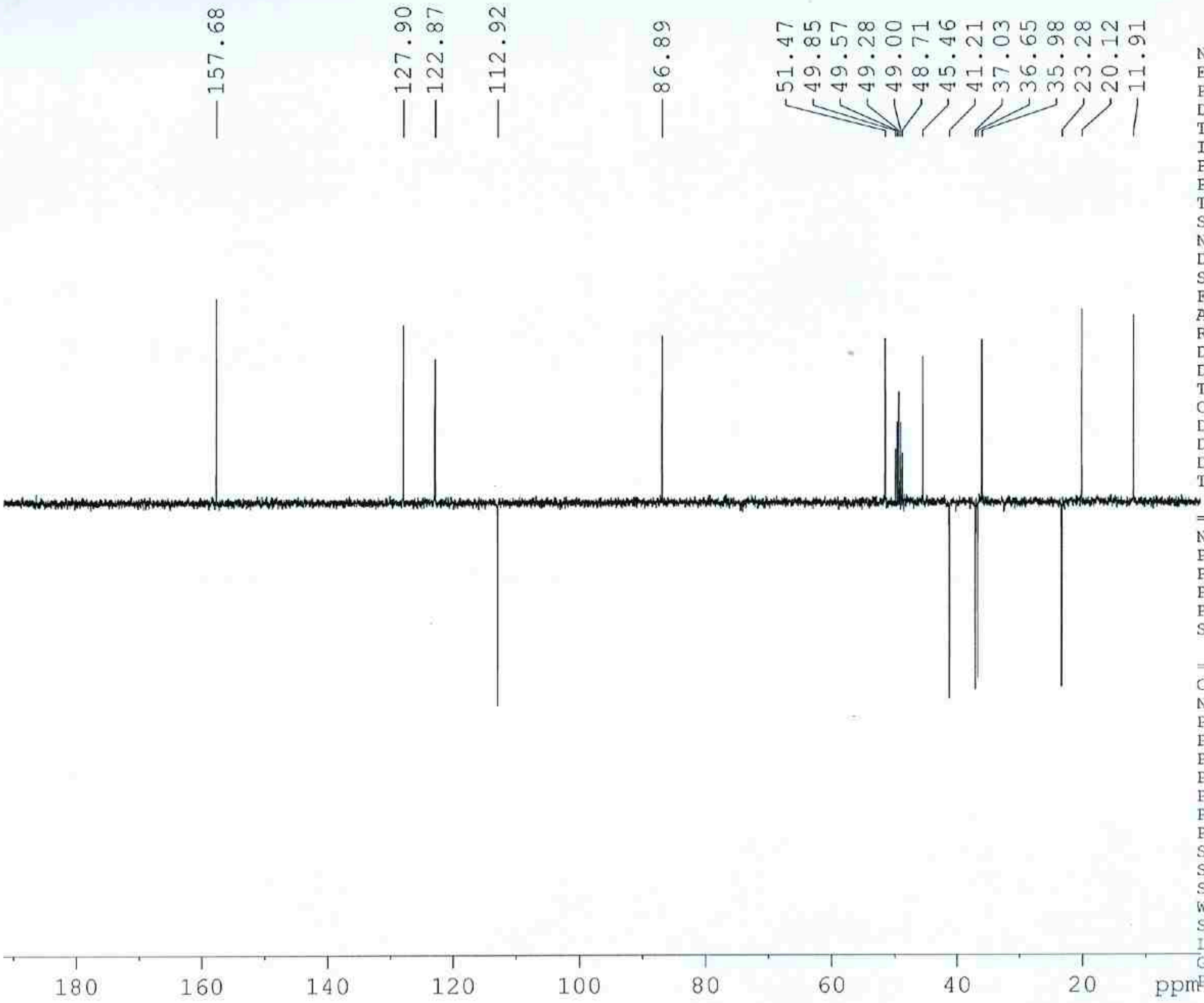

NAME jun08
EXPNO 2
PROCNO 1
Date_ 20120609
Time 1.34
INSTRUM Spect
PROBHD 5 mm BBO BB-1H
PULPROG dept135
TD 32768
SOLVENT MeOD
NS 8192
DS 2
SWH 14367.816 Hz
FIDRES 0.438471 Hz
AQ 1.1403764 sec
RG 203
DW 34.800 usec
DE 6.50 usec
TE 300.0 K
CNST2 145.0000000
D1 1.50000000 sec
D2 0.00344828 sec
D12 0.00002000 sec
TD0 8

======== CHANNEL f1 ========
NUC1 13C
P1 11.00 usec
P2 22.00 usec
PL1 0.00 dB
PL1W 28.88669395 W
SFO1 75.4749184 MHz

======== CHANNEL f2 ========
CPDPRG2 waltz16
NUC2 1H
P3 12.00 usec
P4 24.00 usec
PCPD2 80.00 usec
PL2 0.00 dB
PL12 17.00 dB
PL2W 13.16228485 W
PL12W 0.26262212 W
SFO2 300.1315007 MHz
SI 16384
SF 75.4676422 MHz
WDW EM
SSB 0
LB 1.00 Hz
GB 0
PC 1.00

LAB NO: 109-B

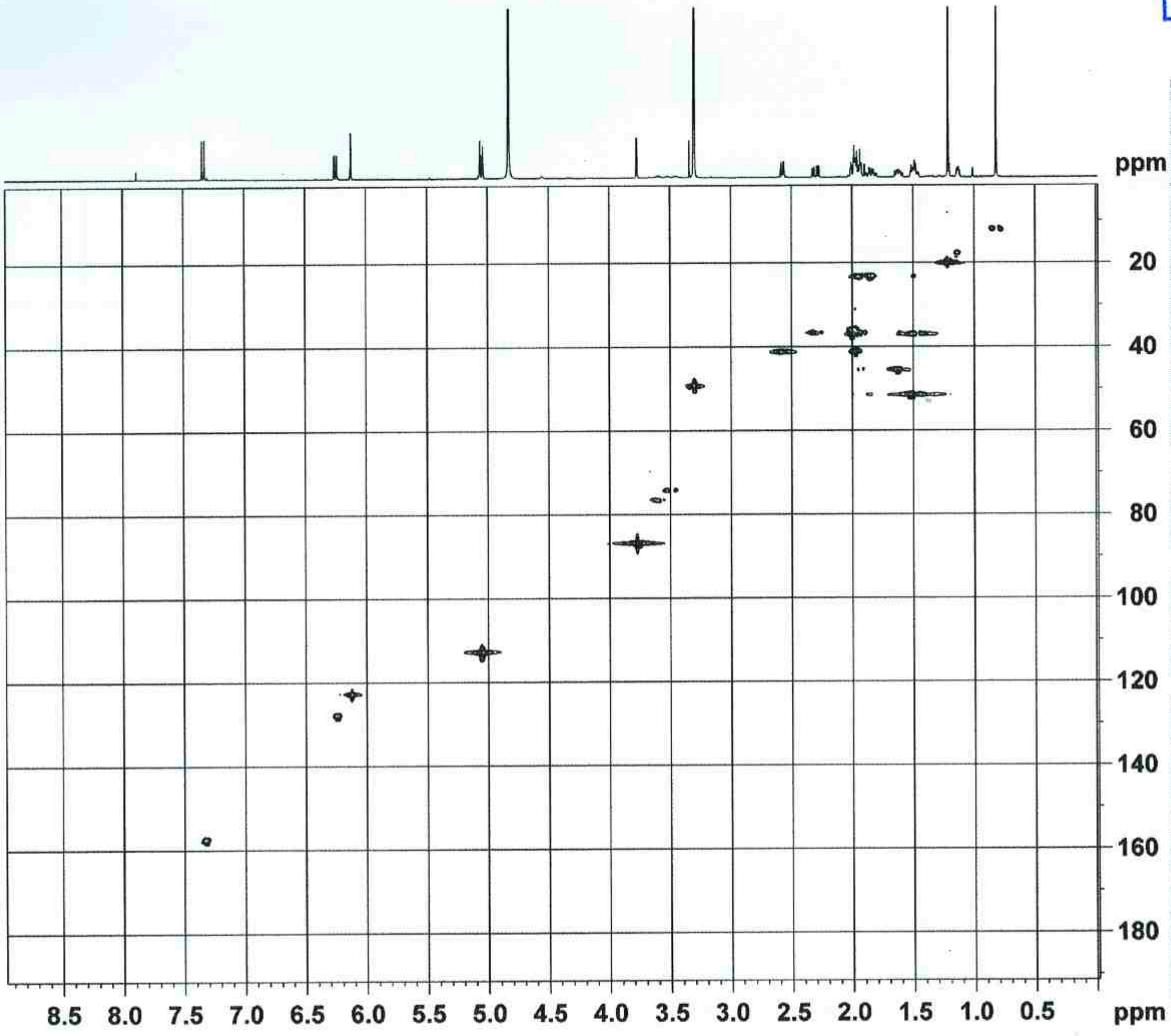

NAME	may29-12
EXPNO	13
PROCNO	1
Date_	20120529
Time	20.50
INSTRUM	spect
PROBHD	5 mm BBI 1H/D-
PULPROG	hsqcetgpsi
TD	1024
SOLVENT	MeOD
NS	32
DS	8
SWH	4496.403 Hz
FIDRES	4.391018 Hz
AQ	0.1140300 sec
RG	23170.5
DW	111.200 usec
DE	6.50 usec
TE	302.4 K
CNST2	145.0000000
D0	0.00000300 sec
D1	1.50000000 sec
D4	0.00172414 sec
D11	0.03000000 sec
D13	0.00000400 sec
D16	0.00010000 sec
D24	0.00110000 sec
IN0	0.00002090 sec
ZGOPTNS	
======== CHANNEL f1 ========	
NUC1	1H
P1	7.60 usec
P2	15.20 usec
P28	0.00 usec
PL1	6.00 dB
SFO1	500.1322506 MHz
======== CHANNEL f2 ========	
CPDPRG2	garp
NUC2	13C
P3	13.25 usec
P4	26.50 usec
PCPD2	65.00 usec
PL2	-2.85 dB
PL12	9.65 dB
SFO2	125.7697360 MHz
====== GRADIENT CHANNEL =====	
GPNAM1	SINE.100
GPNAM2	SINE.100
GPZ1	80.00 %
GPZ2	20.10 %
P16	1000.00 usec
ND0	2
TD	256
SFO1	125.7697 MHz
FIDRES	93.344727 Hz
SW	190.000 ppm
FnMODE	Echo-Antiecho
SI	1024
SF	500.1300158 MHz
WDW	QSINE
SSB	2
LB	0.00 Hz
GB	0
PC	4.00
SI	1024
MC2	echo-antiecho
SF	125.7576112 MHz
WDW	QSINE
SSB	2
LB	0.00 Hz
GB	0

AVANCE AV[illegible]
LAB NO: 109-B

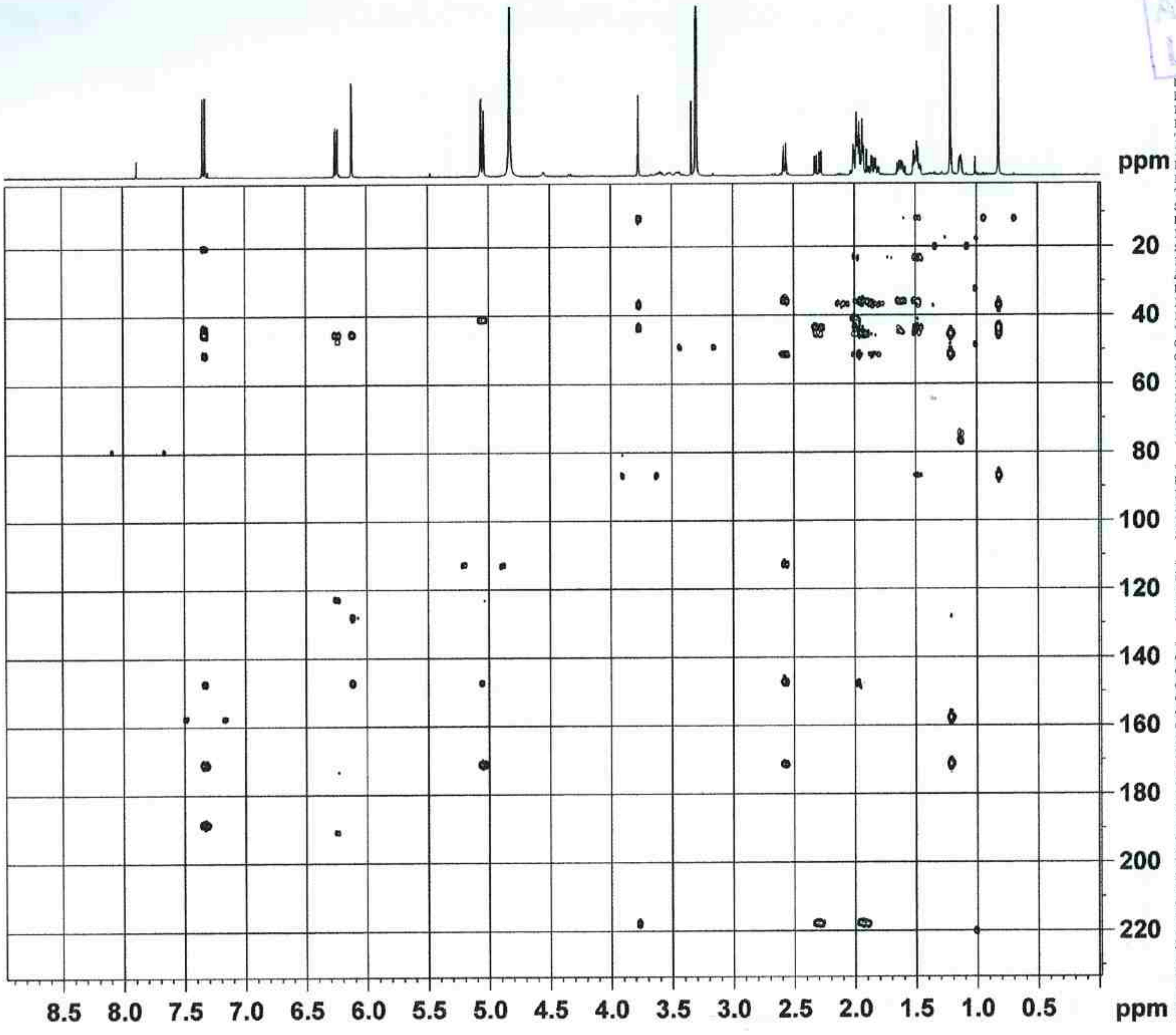

NAME may29-12
EXPNO 14
PROCNO 1
Date_ 20120530
Time 0.35
INSTRUM spect
PROBHD 5 mm BBI 1H/D-
PULPROG hmbcgplpndqf
TD 4096
SOLVENT MeOD
NS 64
DS 8
SWH 4496.403 Hz
FIDRES 1.097755 Hz
AQ 0.4556364 sec
RG 23170.5
DW 111.200 usec
DE 6.50 usec
TE 302.9 K
CNST2 145.0000000
CNST13 10.0000000
D0 0.00000300 sec
D1 2.00000000 sec
D2 0.00344828 sec
D6 0.05000000 sec
D16 0.00010000 sec
IN0 0.00001715 sec

======== CHANNEL f1 ========
NUC1 1H
P1 7.60 usec
P2 15.20 usec
PL1 6.00 dB
SFO1 500.1322506 MHz

======== CHANNEL f2 ========
NUC2 13C
P3 13.25 usec
PL2 -2.85 dB
SFO2 125.7723769 MHz

====== GRADIENT CHANNEL =====
GPNAM1 SINE.100
GPNAM2 SINE.100
GPNAM3 SINE.100
GPZ1 50.00 %
GPZ2 30.00 %
GPZ3 40.10 %
P16 1000.00 usec
ND0 2
TD 199
SFO1 125.7724 MHz
FIDRES 146.629105 Hz
SW 232.000 ppm
FnMODE QF
SI 1024
SF 500.1300158 MHz
WDW QSINE
SSB 0
LB 0.00 Hz
GB 0
PC 4.00
SI 1024
MC2 QF
SF 125.7576112 MHz
WDW QSINE
SSB 0
LB 0.00 Hz
GB 0

LAB NO: [illegible]

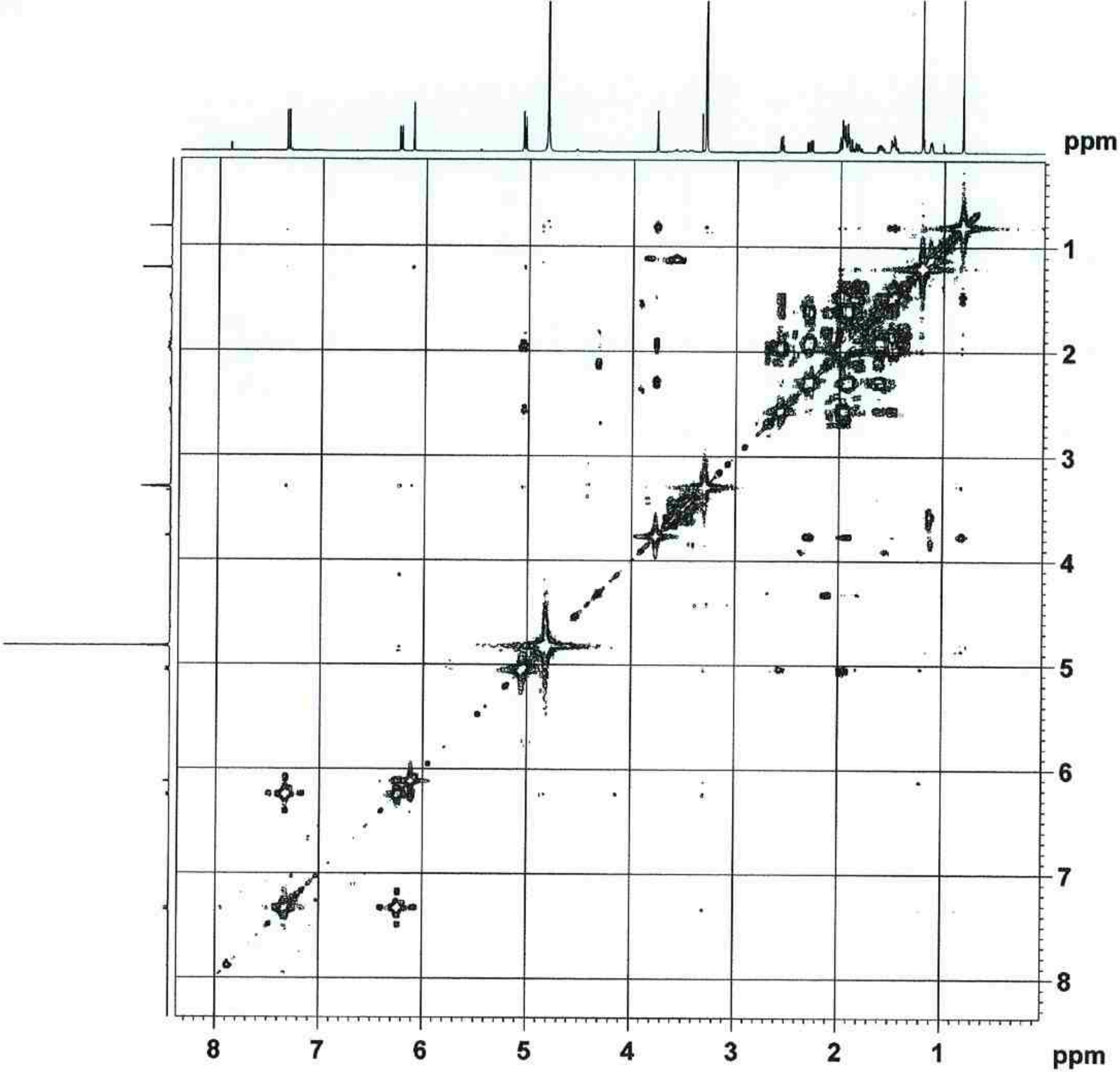

NAME	may29-12
EXPNO	11
PROCNO	1
Date_	20120529
Time	16.55
INSTRUM	spect
PROBHD	5 mm BBI 1H/D-
PULPROG	cosygpqf
TD	2048
SOLVENT	MeOD
NS	8
DS	8
SWH	4496.403 Hz
FIDRES	2.195509 Hz
AQ	0.2278988 sec
RG	256
DW	111.200 usec
DE	6.50 usec
TE	300.1 K
D0	0.00000300 sec
D1	1.50000000 sec
D13	0.00000400 sec
D16	0.00010000 sec
IN0	0.00022240 sec
======== CHANNEL f1 ========	
NUC1	1H
P0	7.60 usec
P1	7.60 usec
PL1	6.00 dB
SFO1	500.1322506 MHz
====== GRADIENT CHANNEL =====	
GPNAM1	SINE.100
GPZ1	10.00 %
P16	1000.00 usec
ND0	1
TD	256
SFO1	500.1323 MHz
FIDRES	17.564074 Hz
SW	8.990 ppm
FnMODE	QF
SI	1024
SF	500.1300158 MHz
WDW	SINE
SSB	0
LB	0.00 Hz
GB	0
PC	4.00
SI	1024
MC2	QF
SF	500.1300158 MHz
WDW	SINE
SSB	0
LB	0.00 Hz
GB	0

LAB NO:

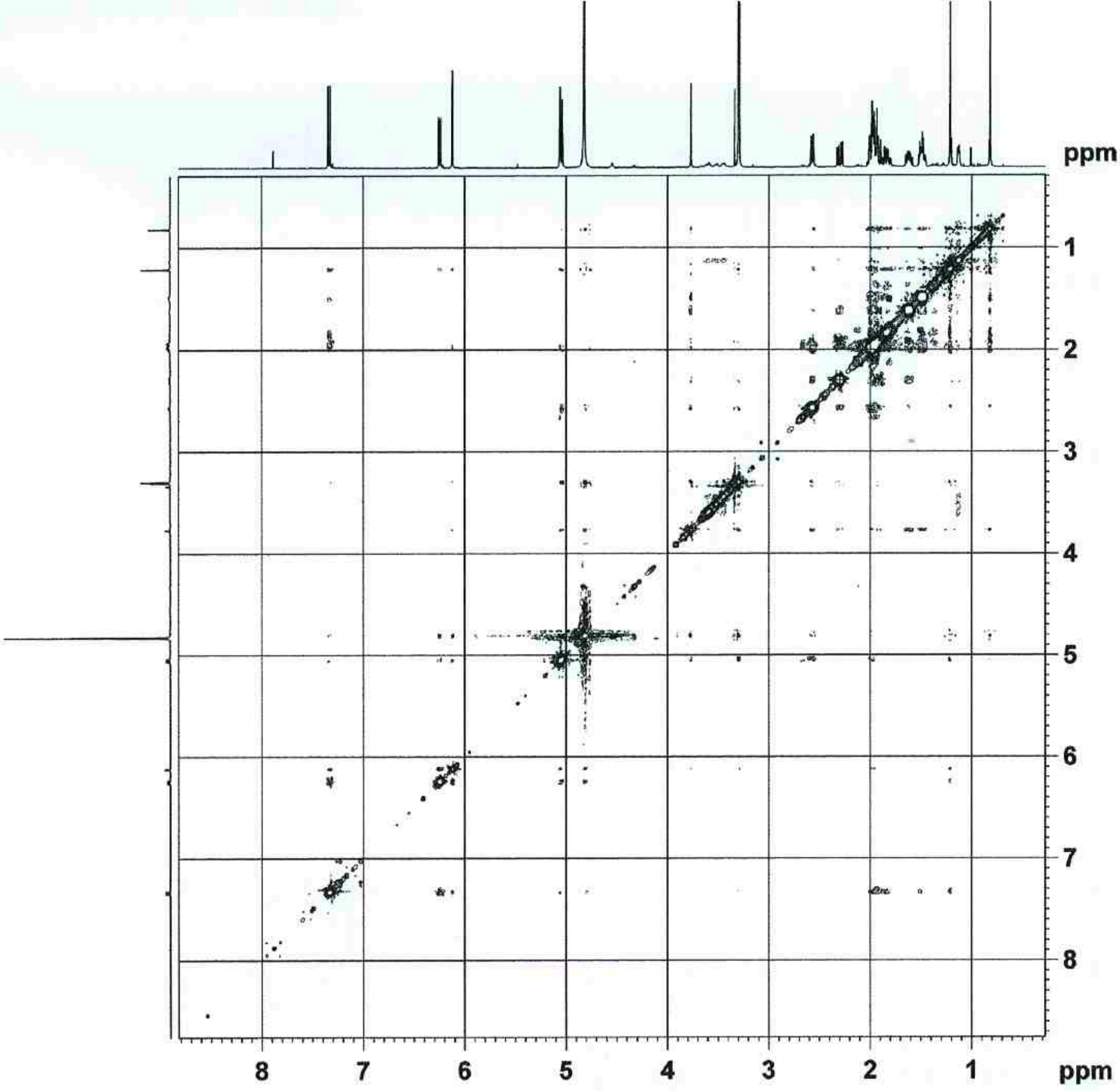

NAME may29-12
EXPNO 12
PROCNO 1
Date_ 20120529
Time_ 17.55
INSTRUM spect
PROBHD 5 mm BBI 1H/D-
PULPROG noesygpph
TD 2048
SOLVENT MeOD
NS 16
DS 2
SWH 4496.403 Hz
FIDRES 2.195509 Hz
AQ 0.2278988 sec
RG 362
DW 111.200 usec
DE 6.50 usec
TE 301.4 K
D0 0.00010152 sec
D1 1.50000000 sec
D8 0.80000001 sec
D16 0.00010000 sec
IN0 0.00022240 sec

======== CHANNEL f1 ========
NUC1 1H
P1 7.60 usec
P2 15.20 usec
PL1 6.00 dB
SFO1 500.1322506 MHz

====== GRADIENT CHANNEL =====
GPNAM1 SINE.100
GPZ1 40.00 %
P16 1000.00 usec
ND0 1
TD 256
SFO1 500.1323 MHz
FIDRES 17.564074 Hz
SW 8.990 ppm
FnMODE States-TPPI
SI 1024
SF 500.1300158 MHz
WDW QSINE
SSB 2
LB 0.00 Hz
GB 0
PC 4.00
SI 1024
MC2 States-TPPI
SF 500.1300158 MHz
WDW QSINE
SSB 2
LB 0.00 Hz
GB 0
