# Supplementary material for: Microbial transformation of anti-cancer steroid exemestane and cytotoxicity of its metabolites against cancer cell lines
Source: Chem Cent J. 2013 Mar 27;7:57. doi: 10.1186/1752-153X-7-57 (PMC3637571; doi:10.1186/1752-153X-7-57)
Supplement: Additional file 4 — Spectroscopic data of metabolite 5. Include spectra of EI-MS, UV, IR, 1H-NMR, 13C-NMR (BB, DEPT-135°, DEPT-90°), HSQC, HMBC, COSY-45°, NOESY experiments. [file 1752-153X-7-57-S4.pdf]

Scan: 11 R.T.: .88

Base: m/z 298; 77.7%FS TIC: 23083178 #Ions: 282

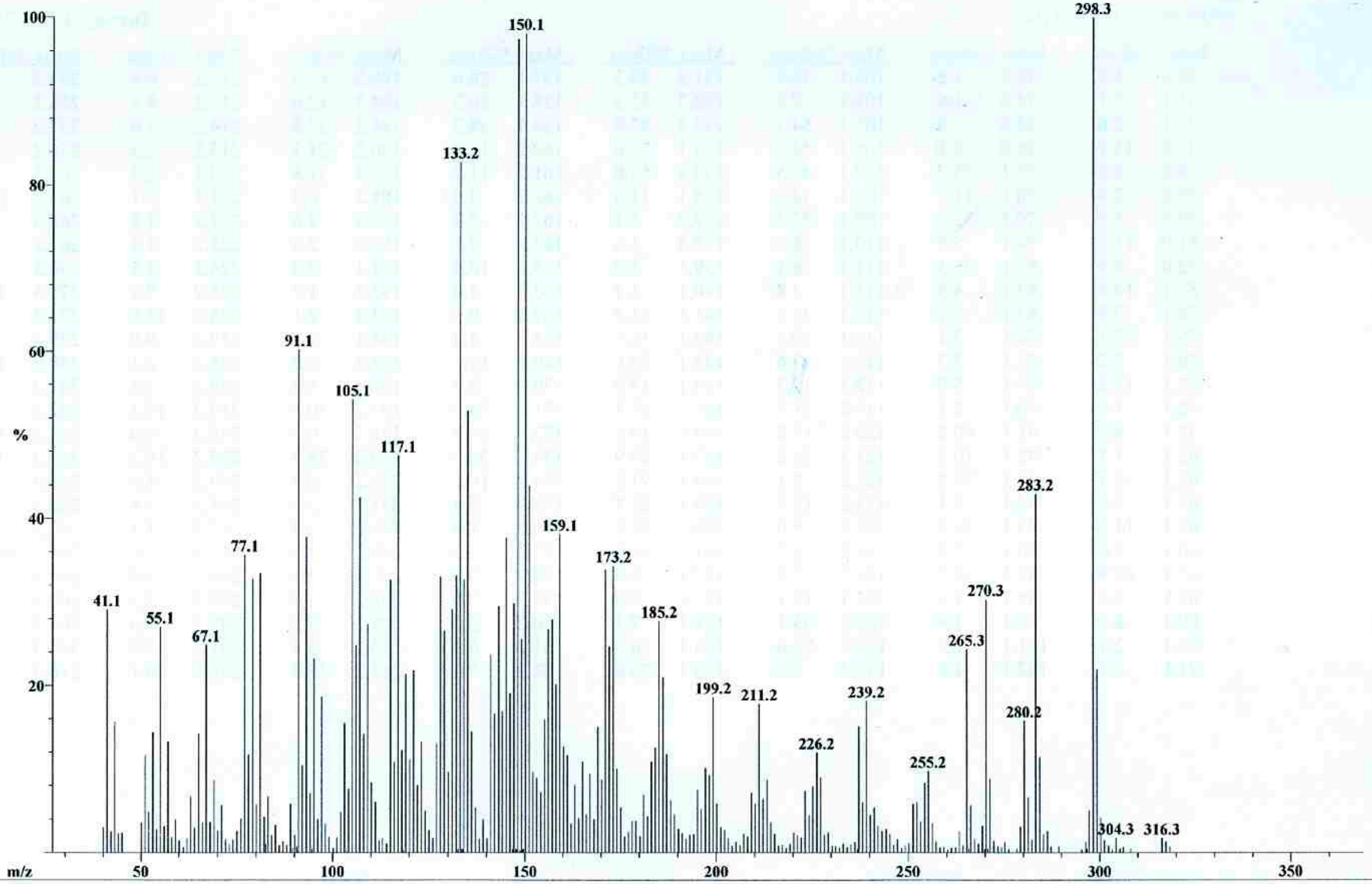

Scan Graph

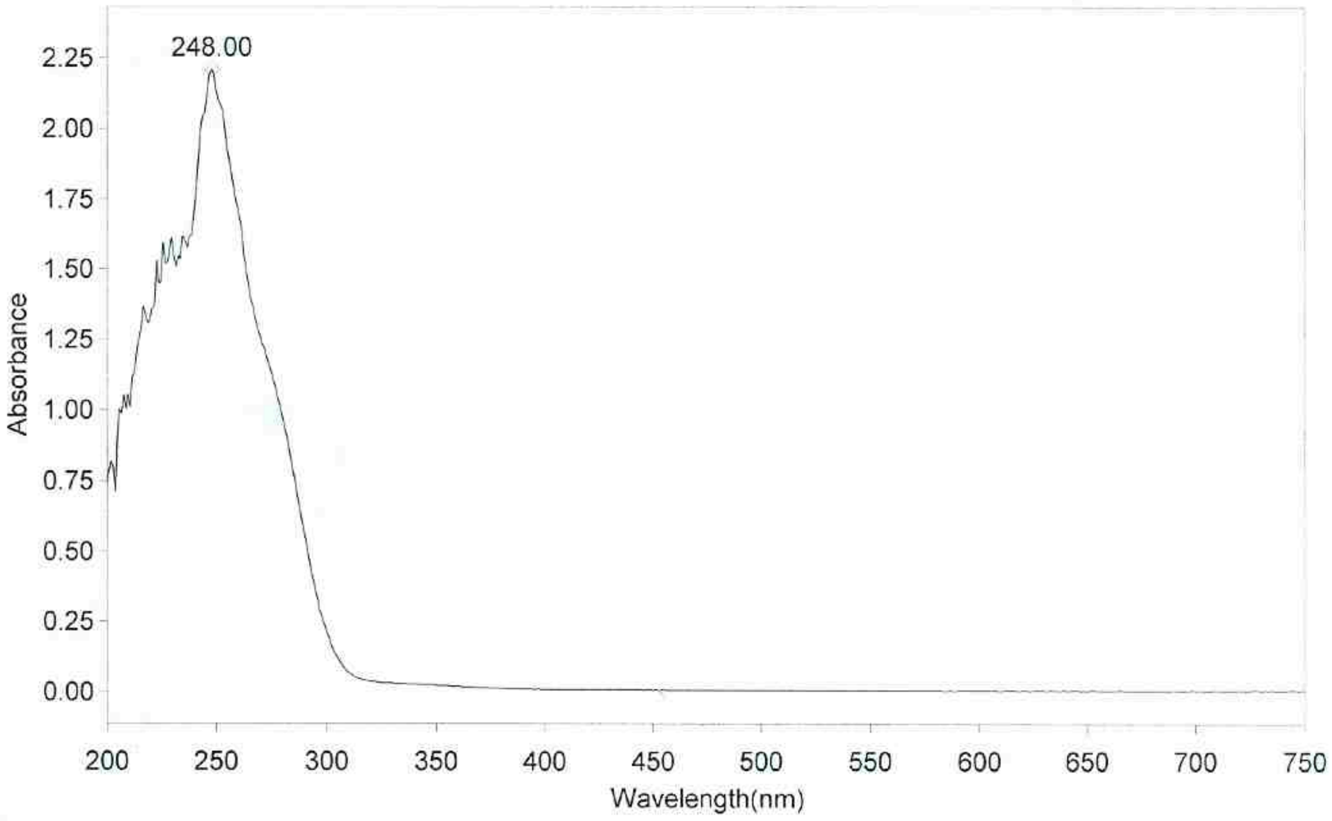

248.00
2.25
2.00
1.75
1.50
1.25
1.00
0.75
0.50
0.25
0.00
Absorbance
200
250
300
350
400
450
500
550
600
650
700
750
Wavelength(nm)

H. E. J. R. I. C., Unive[illegible] of Karachi Analytical Laboratory - Pakistan

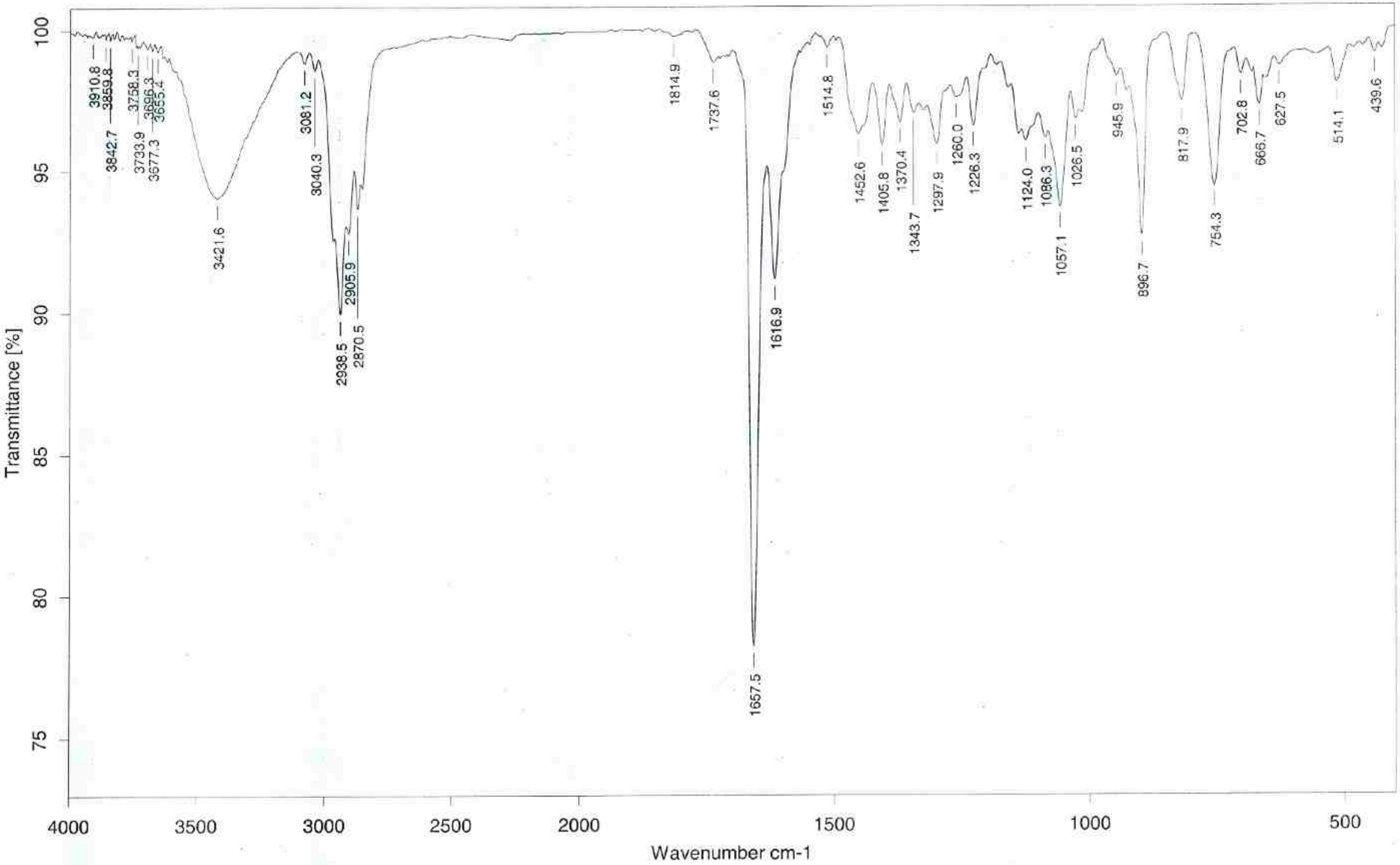

AVANCE AV-300
LAB NO: 115

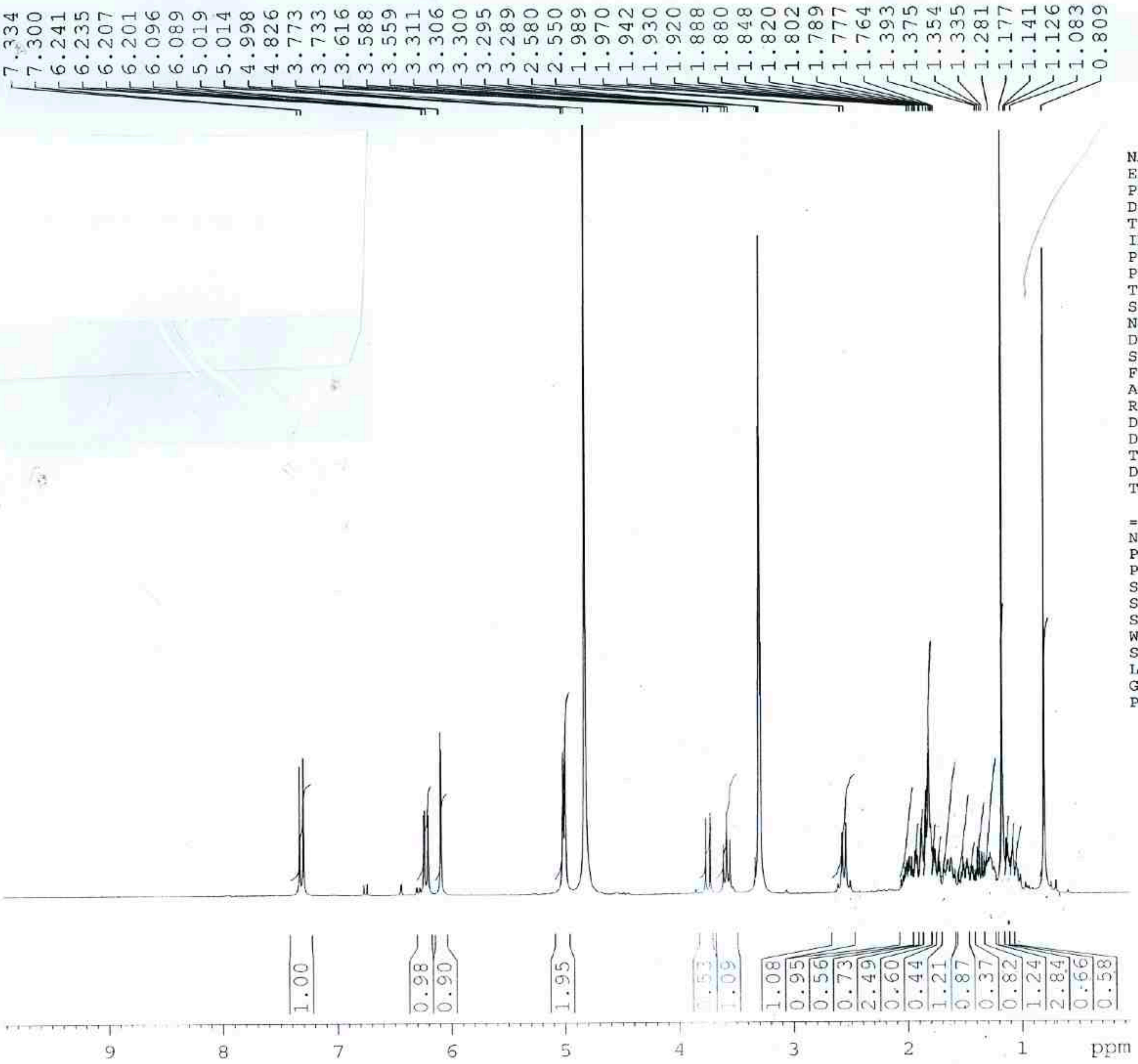

NAME	april18-12
EXPNO	2
PROCNO	1
Date_	20120418
Time	11.14
INSTRUM	spect
PROBHD	5 mm BBO BB-1H
PULPROG	zg30
TD	32768
SOLVENT	MeOD
NS	128
DS	0
SWH	5995.204 Hz
FIDRES	0.182959 Hz
AQ	2.7329011 sec
RG	322.5
DW	83.400 usec
DE	6.50 usec
TE	300.0 K
D1	2.00000000 sec
TD0	1

======== CHANNEL f1 ========

NUC1	1H
P1	7.30 usec
PL1	-3.00 dB
SFO1	300.1324010 MHz
SI	16384
SF	300.1300075 MHz
WDW	EM
SSB	0
LB	0.30 Hz
GB	0
PC	0.50

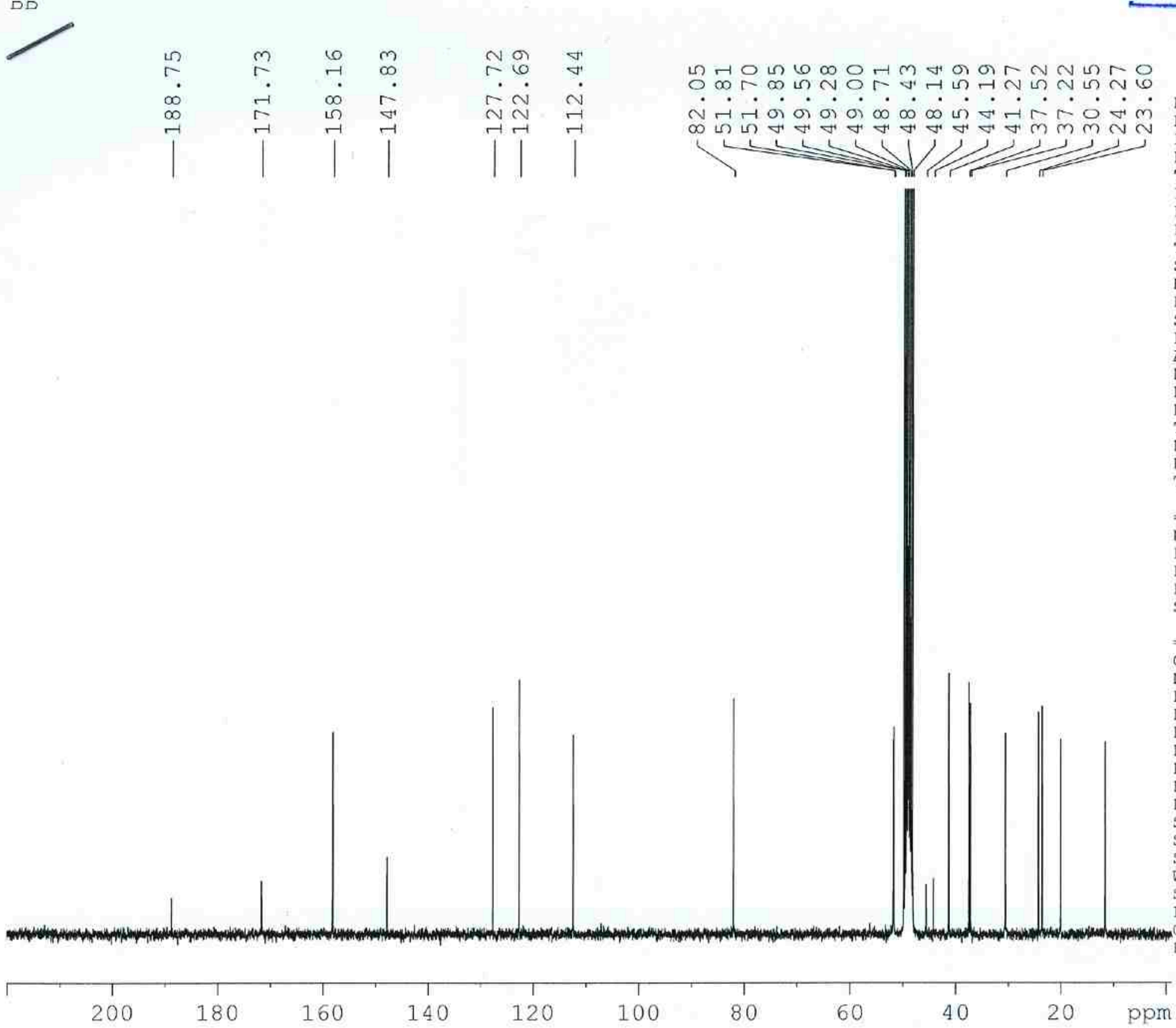

NAME	may16
EXPNO	7
PROCNO	1
Date_	20120516
Time	16.19
INSTRUM	Spect
PROBHD	5 mm BBO BB-1H
PULPROG	zgpg
TD	32768
SOLVENT	MeOD
NS	20480
DS	2
SWH	18028.846 Hz
FIDRES	0.550197 Hz
AQ	0.9088159 sec
RG	28.5
DW	27.733 usec
DE	6.50 usec
TE	300.0 K
D1	1.50000000 sec
D11	0.03000000 sec
TD0	20

======== CHANNEL f1 ========

NUC1	13C
P1	11.00 usec
PL1	0.00 dB
PL1W	28.88669395 W
SFO1	75.4764278 MHz

======== CHANNEL f2 ========

CPDPRG2	waltz16
NUC2	1H
PCPD2	80.00 usec
PL2	120.00 dB
PL12	17.00 dB
PL13	17.00 dB
PL2W	0.00000000 W
PL12W	0.26262212 W
PL13W	0.26262212 W
SFO2	300.1315007 MHz
SI	16384
SF	75.4676422 MHz
WDW	EM
SSB	0
LB	1.00 Hz
GB	0
PC	1.40

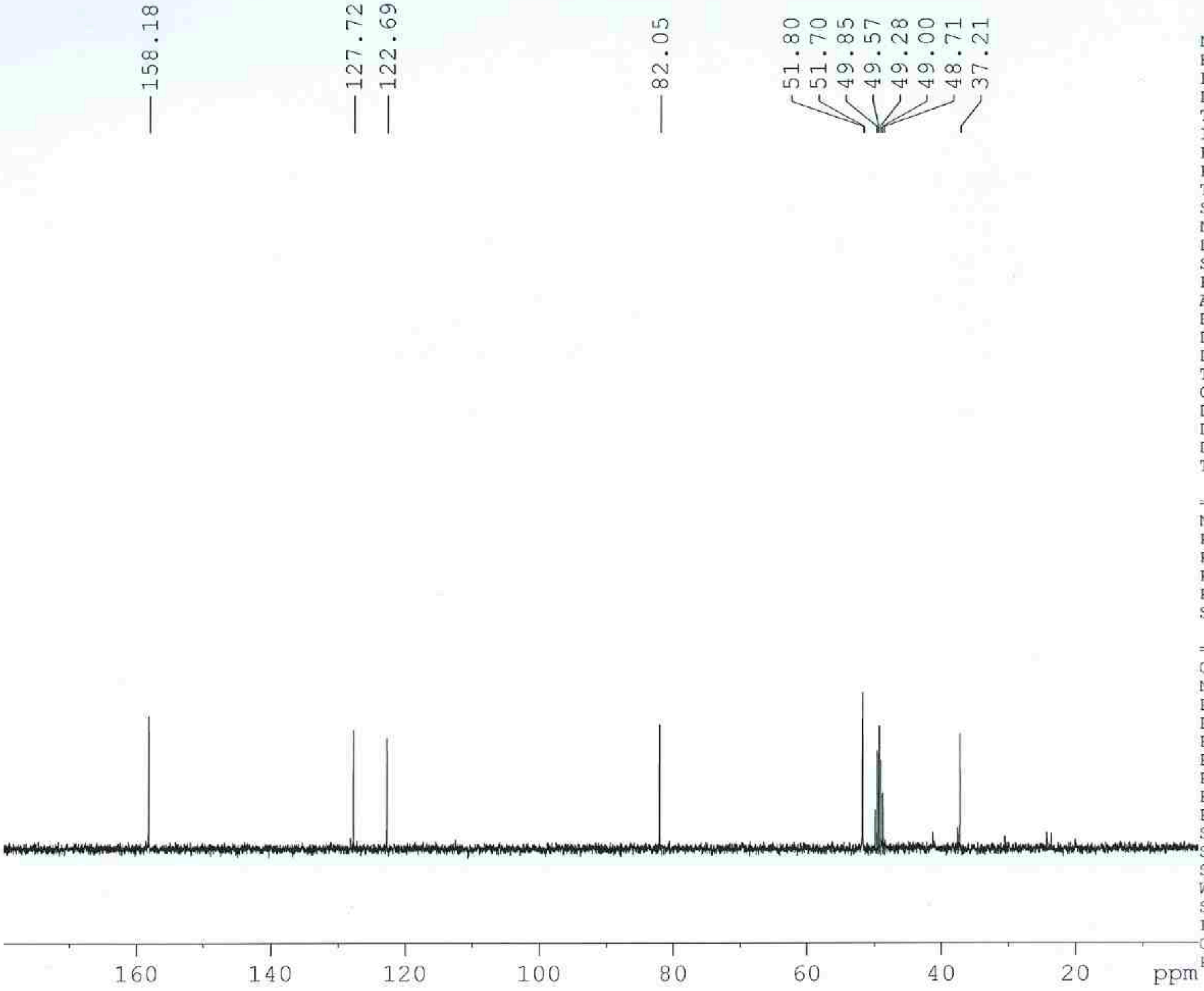

NAME	may16	
EXPNO	9	
PROCNO	1	
Date_	20120517	
Time	9.40	
INSTRUM	Spect	
PROBHD	5 mm BBO BB-1H	
PULPROG	dept90	
TD	32768	
SOLVENT	MeOD	
NS	5106	
DS	2	
SWH	14367.816	Hz
FIDRES	0.438471	Hz
AQ	1.1403764	sec
RG	203	
DW	34.800	usec
DE	6.50	usec
TE	300.0	K
CNST2	145.0000000	
D1	1.50000000	sec
D2	0.00344828	sec
D12	0.00002000	sec
TD0	8	
======== CHANNEL f1 ========		
NUC1	13C	
P1	11.00	usec
P2	22.00	usec
PL1	0.00	dB
PL1W	28.88669395	W
SFO1	75.4749184	MHz
======== CHANNEL f2 ========		
CPDPRG2	waltz16	
NUC2	1H	
P3	12.00	usec
P4	24.00	usec
PCPD2	80.00	usec
PL2	0.00	dB
PL12	17.00	dB
PL2W	13.16228485	W
PL12W	0.26262212	W
SFO2	300.1315007	MHz
SI	16384	
SF	75.4676422	MHz
WDW	EM	
SSB	0	
LB	1.00	Hz
GB	0	
PC	1.00	

DEPT135

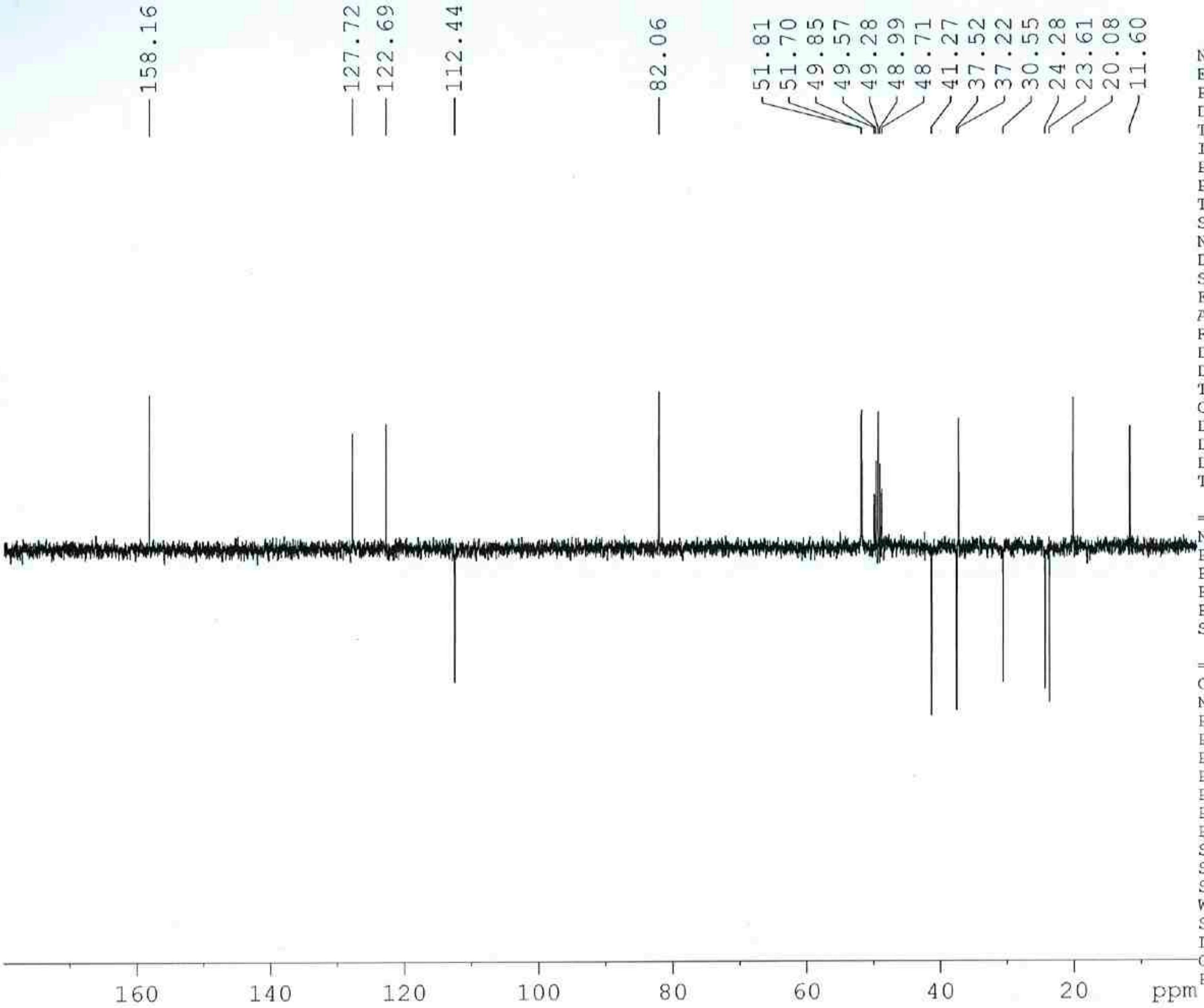

NAME	may16
EXPNO	8
PROCNO	1
Date_	20120517
Time	6.59
INSTRUM	Spect
PROBHD	5 mm BBO BB-1H
PULPROG	dept135
TD	32768
SOLVENT	MeOD
NS	3596
DS	2
SWH	14367.816 Hz
FIDRES	0.438471 Hz
AQ	1.1403764 sec
RG	203
DW	34.800 usec
DE	6.50 usec
TE	300.0 K
CNST2	145.0000000
D1	1.50000000 sec
D2	0.00344828 sec
D12	0.00002000 sec
TD0	12
======== CHANNEL f1 ========	
NUC1	13C
P1	11.00 usec
P2	22.00 usec
PL1	0.00 dB
PL1W	28.88669395 W
SFO1	75.4749184 MHz
======== CHANNEL f2 ========	
CPDPRG2	waltz16
NUC2	1H
P3	12.00 usec
P4	24.00 usec
PCPD2	80.00 usec
PL2	0.00 dB
PL12	17.00 dB
PL2W	13.16228485 W
PL12W	0.26262212 W
SFO2	300.1315007 MHz
SI	16384
SF	75.4676422 MHz
WDW	EM
SSB	0
LB	1.00 Hz
GB	0
PC	1.00

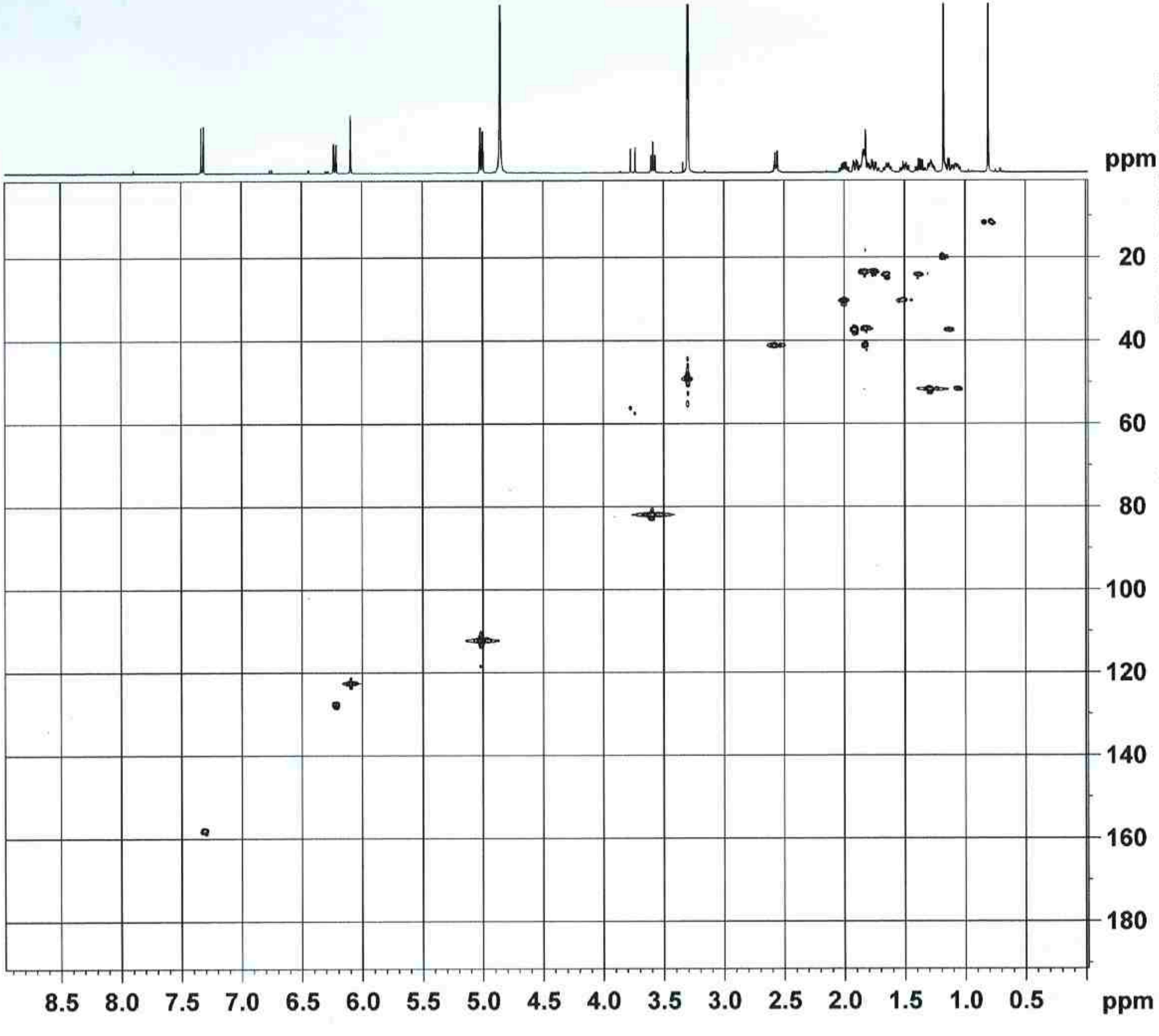

NAME	apr26-12
EXPNO	9
PROCNO	1
Date_	20120426
Time	18.24
INSTRUM	spect
PROBHD	5 mm BBI 1H/D-
PULPROG	hsqcetgpsi
TD	1024
SOLVENT	MeOD
NS	32
DS	8
SWH	4496.403 Hz
FIDRES	4.391018 Hz
AQ	0.1140300 sec
RG	23170.5
DW	111.200 usec
DE	6.50 usec
TE	298.8 K
CNST2	145.0000000
D0	0.00000300 sec
D1	1.50000000 sec
D4	0.00172414 sec
D11	0.03000000 sec
D13	0.00000400 sec
D16	0.00010000 sec
D24	0.00110000 sec
IN0	0.00002090 sec
ZGOPTNS	

======== CHANNEL f1 ========

NUC1	1H
P1	7.60 usec
P2	15.20 usec
P28	0.00 usec
PL1	6.00 dB
SFO1	500.1322506 MHz

======== CHANNEL f2 ========

CPDPRG2	garp
NUC2	13C
P3	13.25 usec
P4	26.50 usec
PCPD2	65.00 usec
PL2	-2.85 dB
PL12	9.65 dB
SFO2	125.7697360 MHz

====== GRADIENT CHANNEL =====

GPNAM1	SINE.100
GPNAM2	SINE.100
GPZ1	80.00 %
GPZ2	20.10 %
P16	1000.00 usec
ND0	2
TD	256
SFO1	125.7697 MHz
FIDRES	93.344727 Hz
SW	190.000 ppm
FnMODE	Echo-Antiecho
SI	1024
SF	500.1300158 MHz
WDW	QSINE
SSB	2
LB	0.00 Hz
GB	0
PC	4.00
SI	1024
MC2	echo-antiecho
SF	125.7576112 MHz
WDW	QSINE
SSB	2
LB	0.00 Hz
GB	0

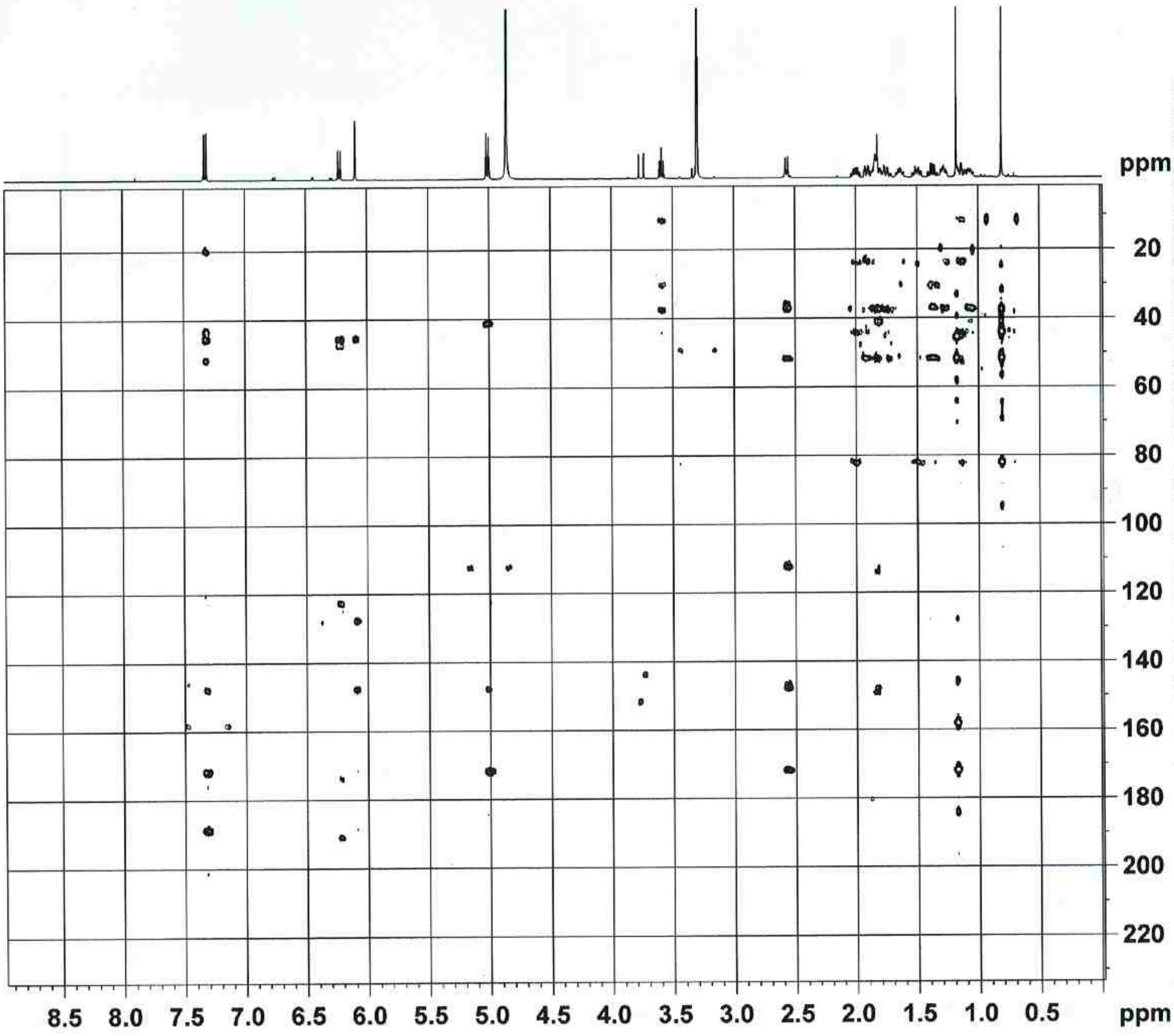

NAME	apr26-12
EXPNO	10
PROCNO	1
Date_	20120426
Time	22.09
INSTRUM	spect
PROBHD	5 mm BBI 1H/D-
PULPROG	hmbcgplpndqf
TD	4096
SOLVENT	MeOD
NS	64
DS	8
SWH	4496.403 Hz
FIDRES	1.097755 Hz
AQ	0.4556364 sec
RG	23170.5
DW	111.200 usec
DE	6.50 usec
TE	301.5 K
CNST2	145.0000000
CNST13	10.0000000
D0	0.00000300 sec
D1	2.00000000 sec
D2	0.00344828 sec
D6	0.05000000 sec
D16	0.00010000 sec
IN0	0.00001715 sec

======== CHANNEL f1 ========

NUC1	1H
P1	7.60 usec
P2	15.20 usec
PL1	6.00 dB
SFO1	500.1322506 MHz

======== CHANNEL f2 ========

NUC2	13C
P3	13.25 usec
PL2	-2.85 dB
SFO2	125.7723769 MHz

====== GRADIENT CHANNEL =====

GPNAM1	SINE.100
GPNAM2	SINE.100
GPNAM3	SINE.100
GPZ1	50.00 %
GPZ2	30.00 %
GPZ3	40.10 %
P16	1000.00 usec
ND0	2
TD	256
SFO1	125.7724 MHz
FIDRES	113.981216 Hz
SW	232.000 ppm
FnMODE	QF
SI	1024
SF	500.1300158 MHz
WDW	QSINE
SSB	0
LB	0.00 Hz
GB	0
PC	4.00
SI	1024
MC2	QF
SF	125.7576112 MHz
WDW	QSINE
SSB	0
LB	0.00 Hz
GB	0

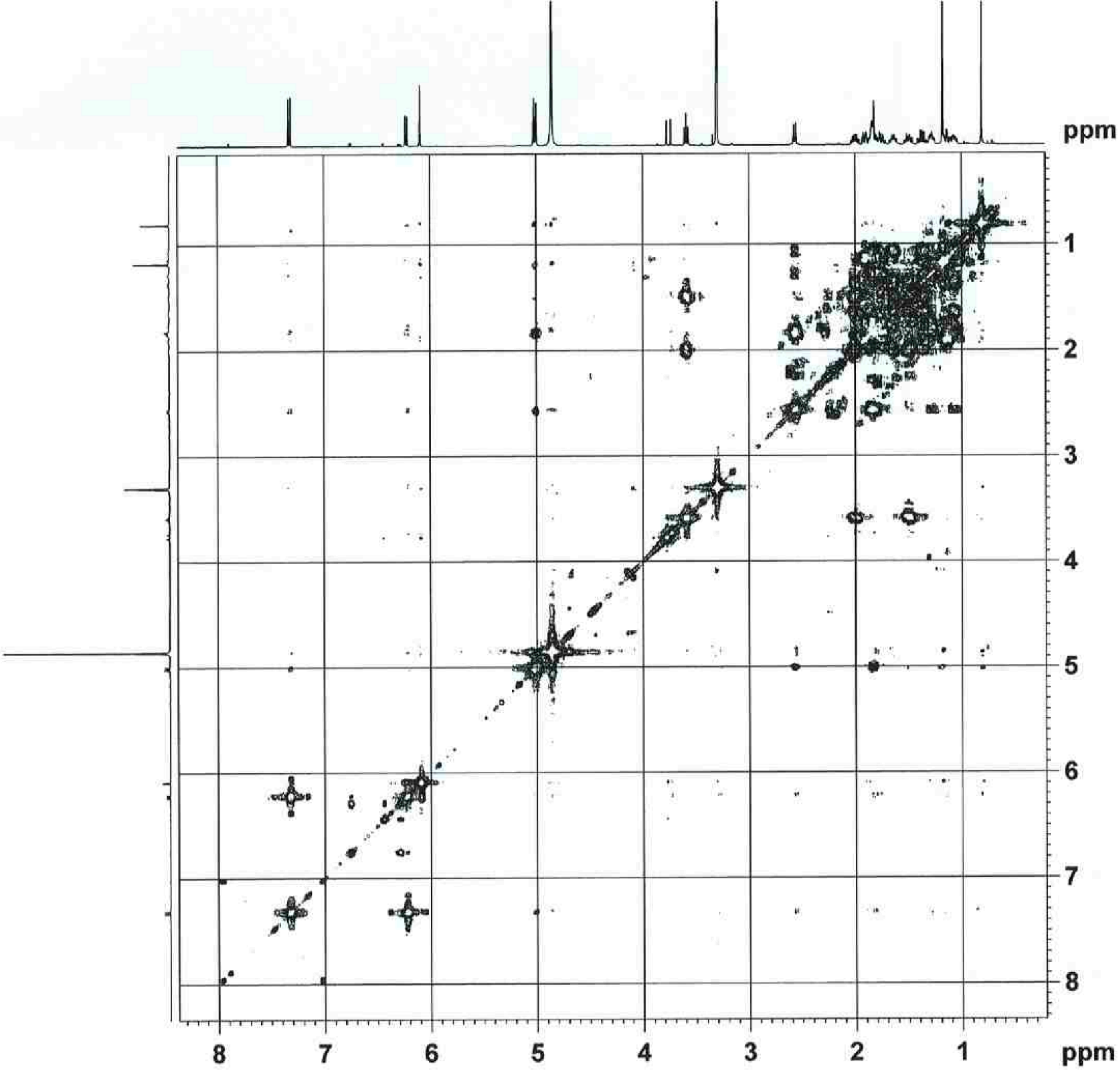

NAME	apr26-12	
EXPNO	7	
PROCNO	1	
Date_	20120426	
Time	14.29	
INSTRUM	spect	
PROBHD	5 mm BBI 1H/D-	
PULPROG	cosygpqf	
TD	2048	
SOLVENT	MeOD	
NS	8	
DS	8	
SWH	4496.403	Hz
FIDRES	2.195509	Hz
AQ	0.2278988	sec
RG	574.7	
DW	111.200	usec
DE	6.50	usec
TE	297.0	K
D0	0.00000300	sec
D1	1.50000000	sec
D13	0.00000400	sec
D16	0.00010000	sec
IN0	0.00022240	sec

======== CHANNEL f1 ========

NUC1	1H	
P0	7.60	usec
P1	7.60	usec
PL1	6.00	dB
SFO1	500.1322506	MHz

====== GRADIENT CHANNEL =====

GPNAM1	SINE.100	
GPZ1	10.00	%
P16	1000.00	usec
ND0	1	
TD	256	
SFO1	500.1323	MHz
FIDRES	17.564074	Hz
SW	8.990	ppm
FnMODE	QF	
SI	1024	
SF	500.1300158	MHz
WDW	SINE	
SSB	0	
LB	0.00	Hz
GB	0	
PC	4.00	
SI	1024	
MC2	QF	
SF	500.1300158	MHz
WDW	SINE	
SSB	0	
LB	0.00	Hz
GB	0	

LAB NO: 1

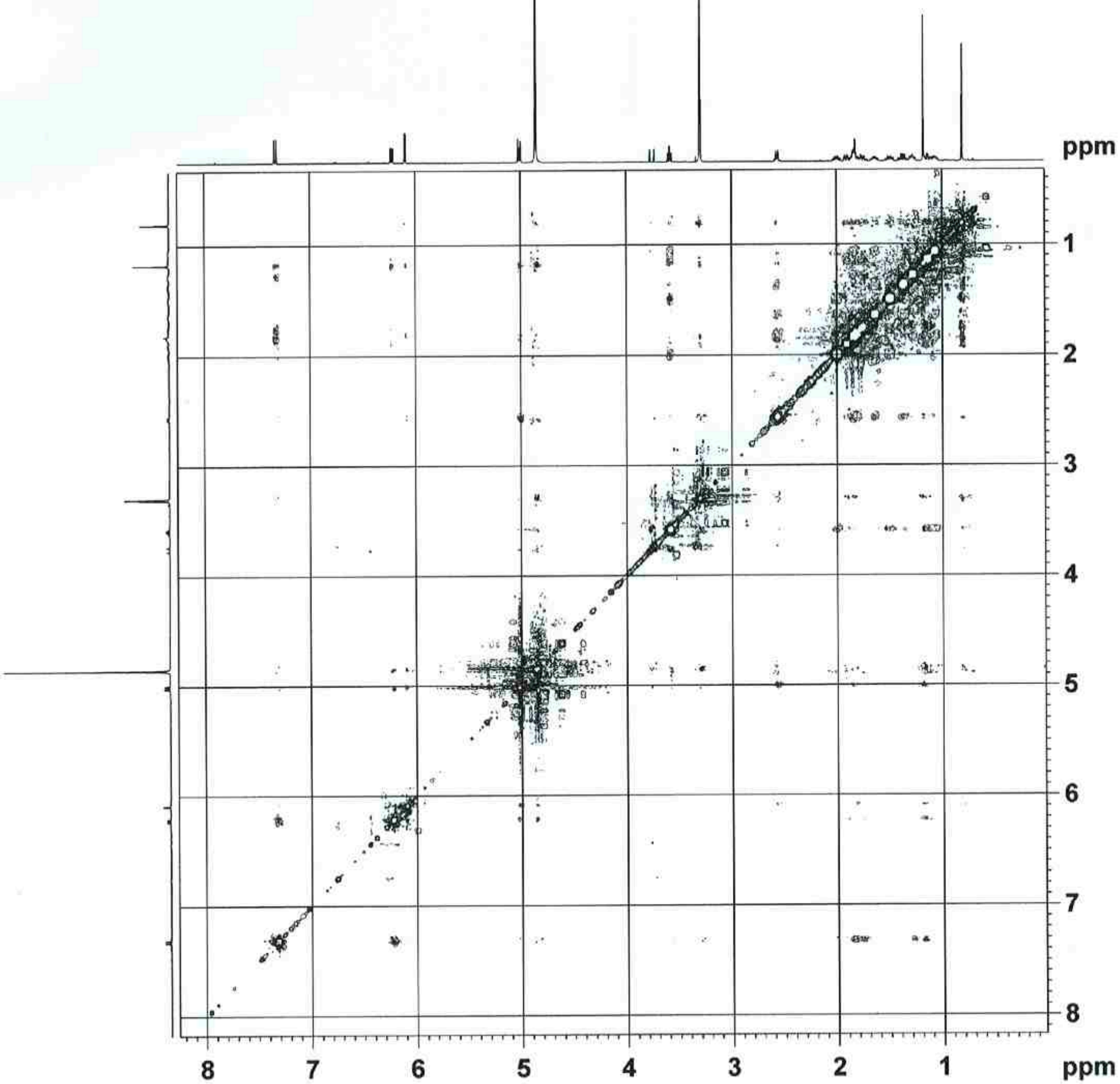

NAME	apr26-12
EXPNO	8
PROCNO	1
Date_	20120426
Time	15.30
INSTRUM	spect
PROBHD	5 mm BBI 1H/D-
PULPROG	noesygpph
TD	2048
SOLVENT	MeOD
NS	16
DS	2
SWH	4496.403 Hz
FIDRES	2.195509 Hz
AQ	0.2278988 sec
RG	574.7
DW	111.200 usec
DE	6.50 usec
TE	296.9 K
D0	0.00010152 sec
D1	1.50000000 sec
D8	0.80000001 sec
D16	0.00010000 sec
IN0	0.00022240 sec

======== CHANNEL f1 ========

NUC1	1H
P1	7.60 usec
P2	15.20 usec
PL1	6.00 dB
SFO1	500.1322506 MHz

====== GRADIENT CHANNEL =====

GPNAM1	SINE.100
GPZ1	40.00 %
P16	1000.00 usec
ND0	1
TD	256
SFO1	500.1323 MHz
FIDRES	17.564074 Hz
SW	8.990 ppm
FnMODE	States-TPPI
SI	1024
SF	500.1300158 MHz
WDW	QSINE
SSB	2
LB	0.00 Hz
GB	0
PC	4.00
SI	1024
MC2	States-TPPI
SF	500.1300158 MHz
WDW	QSINE
SSB	2
LB	0.00 Hz
GB	0
